# Machine learning and phone data can improve targeting of humanitarian aid

**DOI:** 10.1038/s41586-022-04484-9

**Published:** 2022-03-16

**Authors:** Emily Aiken, Suzanne Bellue, Dean Karlan, Chris Udry, Joshua E. Blumenstock

**Affiliations:** 1 School of Information, University of California, Berkeley, CA USA; 2 Department of Economics, University of Mannheim, Mannheim, Germany; 3 Kellogg School of Management, Global Poverty Research Lab, Northwestern University, Evanston, IL USA; 4 Department of Economics, Global Poverty Research Lab, Northwestern University, Evanston, IL USA

**Keywords:** Computer science, Developing world, Policy, Economics

## Abstract

The COVID-19 pandemic has devastated many low- and middle-income countries, causing widespread food insecurity and a sharp decline in living standards^1^. In response to this crisis, governments and humanitarian organizations worldwide have distributed social assistance to more than 1.5 billion people^2^. Targeting is a central challenge in administering these programmes: it remains a difficult task to rapidly identify those with the greatest need given available data^3,4^. Here we show that data from mobile phone networks can improve the targeting of humanitarian assistance. Our approach uses traditional survey data to train machine-learning algorithms to recognize patterns of poverty in mobile phone data; the trained algorithms can then prioritize aid to the poorest mobile subscribers. We evaluate this approach by studying a flagship emergency cash transfer program in Togo, which used these algorithms to disburse millions of US dollars worth of COVID-19 relief aid. Our analysis compares outcomes—including exclusion errors, total social welfare and measures of fairness—under different targeting regimes. Relative to the geographic targeting options considered by the Government of Togo, the machine-learning approach reduces errors of exclusion by 4–21%. Relative to methods requiring a comprehensive social registry (a hypothetical exercise; no such registry exists in Togo), the machine-learning approach increases exclusion errors by 9–35%. These results highlight the potential for new data sources to complement traditional methods for targeting humanitarian assistance, particularly in crisis settings in which traditional data are missing or out of date.

## Abstract (web summary)

Machine-learning algorithms can take advantage of survey and mobile phone data to help to identify people most in need of aid, complementing traditional methods for targeting humanitarian assistance.

## Main

The COVID-19 pandemic has led to a sharp decline in living standards across the world, as policies designed to stop the spread of the disease have disrupted normal economic activity. Economically vulnerable households in low- and middle-income countries have been among the hardest hit, with more than 100 million individuals estimated to have transitioned into extreme poverty since the onset of the pandemic^5^.

To offset the most severe consequences of this sudden decline in income, governments and humanitarian organizations around the world have mobilized relief efforts. It has been estimated that more than 3,300 new social assistance programmes have been launched^2^ since early 2020, providing more than US$800 billion in cash transfer payments to over 1.5 billion people (roughly one fifth of the world’s population).

The overwhelming majority of COVID-19 response efforts—and the majority of cash transfer programmes globally—provide targeted social assistance^3,4^. In other words, specific criteria—typically a proxy for socioeconomic status—are used to determine potential eligibility. In most wealthy nations, governments rely on recent household income data to determine programme eligibility^6^. However, in low- and lower middle-income countries (LMICs), where economic activity is often informal and based on home-produced agriculture, governments typically do not observe income for the vast majority of the population^3^. Other potential sources of targeting data are often incomplete or out of date^7,8^; for example, only half of the poorest countries have completed a census in the past 10 years^9^. In such contexts, data gaps preclude governments from implementing well-targeted social assistance programmes^10,11^.

Here we develop, implement and evaluate an approach to targeting social assistance based on machine-learning algorithms and non-traditional ‘big data’ from satellites and mobile phone networks. This approach leverages recent advances in machine learning that show that such data can help accurately estimate the wealth of small geographic regions^12–16^ and individual mobile subscribers^17–19^. It also builds on a rich economics literature on the design of appropriate mechanisms for targeting social assistance^3,20–29^. See Supplementary Discussion, section 1 for a summary of previous work.

## Humanitarian response to COVID-19 in Togo

Our results are based on the design and evaluation of Novissi, a flagship emergency social assistance programme carried out in Togo. The Government of Togo launched Novissi in April 2020, shortly after the first cases of COVID-19 appeared in the country. As economic lockdown orders forced many Togolese to stop working and led to widespread food insecurity (Supplementary Fig. 1), Novissi aimed to provide subsistence cash relief to those most affected (see https://novissi.gouv.tg/). Eligible beneficiaries received bi-weekly payments of roughly US$10. In an effort to minimize in-person contact, Novissi enrolment and payments were implemented digitally: beneficiaries registered using their mobile phones and transfers were made via mobile money. Full details on the Novissi programme are provided in Methods, ‘The COVID-19 pandemic in Togo’.

When the government first launched Novissi, it did not have a traditional social registry that could be used to assess programme eligibility, and did not have the time or the resources to build such a registry in the middle of the pandemic. The most recent census, which was completed in 2011, did not contain information on household wealth or poverty; more recent national surveys on living standards only contacted a small fraction of all households (Methods, ‘The COVID-19 pandemic in Togo’). Instead, eligibility for Novissi was determined on the basis of data contained in a national voter registry that had been updated in late 2019. Specifically, benefits were initially disbursed to individuals who met three criteria: (1) ‘self-targeted’^20^ by dialling in to the Novissi platform and entering basic information from their mobile phone; (2) registered to vote in specific regions (the programme initially focused on the Greater Lomé region around the capital city); and (3) self-declared to work in an informal occupation in their voter registration. The decision to target informal occupations helped prioritize benefits to people who were forced to stop working at the onset of the crisis. However, this approach does not necessarily target benefits to the poorest households in the country (Supplementary Fig. 2).

**Extended Data Fig. 1:**
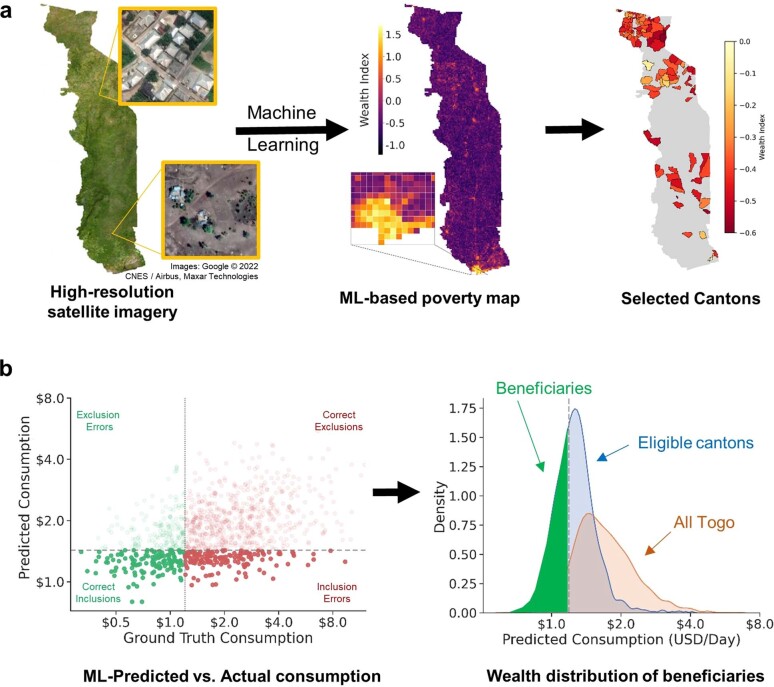
Overview of targeting methodology. **a)** Regional targeting. Satellite imagery of Togo^59^ is used to construct micro-estimates of poverty (middle)^16^, which are overlayed with population data to produce canton-level estimates of wealth. Individuals registered in the 100 poorest cantons (right) are eligible for benefits. Inset images © 2019 Google. **b)** Individual targeting. A machine-learning algorithm is trained using representative survey data to predict consumption from features of phone use (Methods, ‘ Machine-learning methods’). The algorithm constructs poverty scores that are correlated with ground-truth measures of consumption (left). Subscribers who register for the program in targeted cantons with estimated consumption less than USD $1.25/day are eligible for benefits (right). The red distribution shows the predicted wealth distribution of the entire population of Togo; the blue distribution shows the predicted wealth distribution in the 100 poorest cantons; and the green section indicates the predicted wealth distribution of Novissi beneficiaries.

Our research efforts focused on helping the government expand the Novissi programme from informal workers in Greater Lomé to poorer individuals in rural regions of the country, and were designed to meet the government’s two stated policy objectives: first, to direct benefits to the poorest geographic regions of the country; and second, to prioritize benefits to the poorest mobile subscribers in those regions. (Individuals without access to a mobile phone could not receive Novissi payments, which were delivered digitally using mobile money; see Methods, ‘Programme exclusions’ for details.) The approach we developed, which uses machine learning to analyse non-traditional data from satellites and mobile phone networks, has two distinct steps (Extended Data Fig. 1).

## Targeting with mobile phone data

In the first step, we obtained public micro-estimates of the relative wealth of every 2.4 km by 2.4 km region in Togo, which were constructed by applying machine-learning algorithms to high-resolution satellite imagery^16^. These estimates provide an indication of the relative wealth of all the households in each small grid cell; we take the population-weighted average of these grid cells to estimate the average wealth of every canton, Togo’s smallest administrative unit (see Methods, ‘Poverty maps’).

In the second step, we estimated the average daily consumption of each mobile phone subscriber by applying machine-learning algorithms to mobile phone metadata provided by Togo’s two mobile phone operators (see Methods, ‘Data privacy concerns’). Specifically, we conducted surveys with a large and representative sample of mobile phone subscribers, used the surveys to measure the wealth and/or consumption of each subscriber, and then matched the survey-based estimates to detailed metadata on each subscriber’s history of phone use. This sample was used to train supervised machine-learning algorithms that predict wealth and consumption from phone use^17–19^ (Pearson’s *ρ* ranges from 0.41–0.46; Methods, ‘Predicting poverty from phone data’). This second step is similar in spirit to a traditional proxy means test (PMT), with two main differences: we used a high-dimensional vector of mobile phone features instead of a low-dimensional vector of assets to estimate wealth; and we used machine-learning algorithms designed to maximize out-of-sample predictive power instead of the traditional linear regression that maximizes in-sample goodness of fit^30^.

## Evaluation of targeting accuracy

Our main analysis evaluates the performance of this new targeting approach that combines machine learning and mobile phone data—which we refer to more succinctly as the phone-based approach—by comparing targeting errors using this approach to targeting errors under three counterfactual approaches: a geographic targeting approach that the government piloted in summer 2020 (in which all individuals are eligible within the poorest prefectures (Togo’s admin-2 level), or poorest cantons (Togo’s admin-3 level); occupation-based targeting (including Novissi’s original approach to targeting informal workers, as well as an ‘optimal’ approach to targeting the poorest occupation categories in the country); and a parsimonious method based on phone data without machine learning (that uses total expenditures on calling and texting as a proxy for wealth).

We present results that compare the effectiveness of these different targeting mechanisms in two different scenarios. First, we evaluate the actual policy scenario faced by the government of Togo in September of 2020, which involved distributing cash to 60,000 beneficiaries in Togo’s 100 poorest cantons. This first scenario is evaluated using data collected in a large phone survey we designed for this purpose and conducted in September 2020. The ‘ground truth’ measure of poverty in this first scenario is a PMT, as consumption data could not be feasibly collected in the phone survey. The PMT is based on a stepwise regression procedure, described in Supplementary Methods, section 3, which captures roughly 48% of the variation in consumption. Thus, for the first scenario focused on the rural Novissi programme, all targeting methods are evaluated with respect to this PMT. The phone-based machine-learning model is similarly trained using the PMT as ground truth. Second, we simulate and evaluate a more general and hypothetical policy scenario in which the government is interested in targeting the poorest individuals nationwide; this scenario is evaluated using national household survey data collected in person by the government in 2018 and 2019. The second simulation uses consumption as the ground truth measure of poverty. These data are described in the Methods section ‘Data sources’ and details on the evaluation are in the Methods section ‘Targeting evaluations﻿.'

**Fig. 1:**
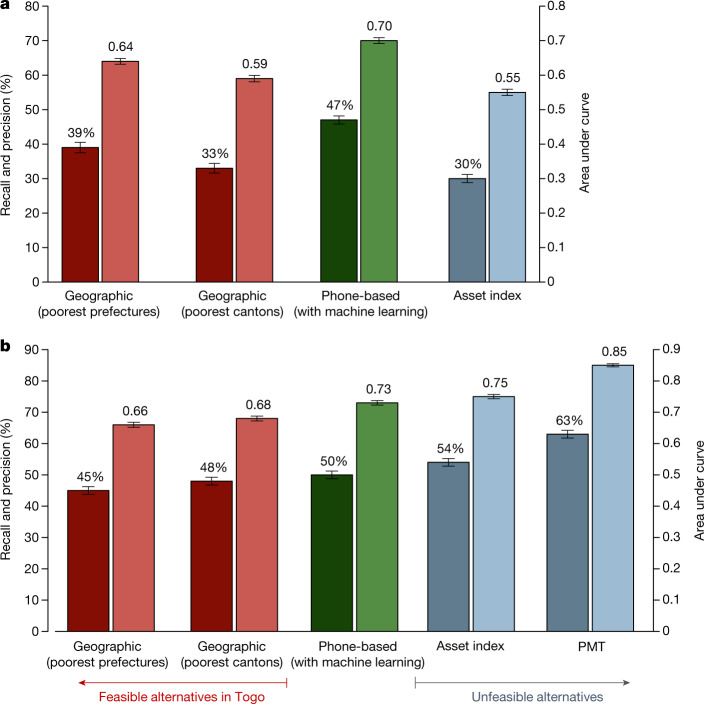
Comparing Novissi targeting to alternatives. **a**, **b**, The performance of phone-based targeting (green) compared with alternative approaches that were feasible (red) and unfeasible (blue) in Togo in 2020. Targeting is evaluated for the actual rural Novissi programme (**a**), which focused on Togo’s 100 poorest cantons (using a 2020 survey representative of mobile subscribers in the 100 cantons, where PMT is a ground truth for poverty since consumption data was not collected in the phone survey); and a hypothetical nationwide anti-poverty programme (using a national field survey conducted in 2018–2019, where consumption is a ground truth for poverty) (**b**). The darker bar in each pair indicates recall and precision (left axis), which is equivalent to 1 – exclusion error; the lighter bar in each pair indicates area under the curve (right axis). The bar height represents the point estimate from the full simulation; whiskers show s.d. produced from *n* = 1,000 bootstrap simulations. The figure highlights a subset of the results contained in Table 1.

In the first scenario focused on reaching the poorest people in the 100 poorest cantons, we find that the phone-based approach to targeting substantially reduces errors of exclusion (true poor who are mistakenly deemed ineligible) and errors of inclusion (non-poor who are mistakenly deemed eligible) relative to the other feasible approaches to targeting available to the government of Togo (Fig. 1a and Table 1, columns 3 to 6). We focus on the ability of each targeting method to reach the poorest 29% in each of the two survey datasets, as the rural expansion of Novissi only had sufficient funding to provide benefits to 29% of individuals in eligible geographies (Extended Data Tables 1, 2 evaluate performance using alternative poverty thresholds). Using a PMT as a measure of ‘true’ poverty status, phone-based targeting (area under the curve (AUC) = 0.70) outperforms the other feasible methods of targeting rural Novissi aid (for example, AUC = 0.59–0.64 for geographic blanket targeting). As a result, errors of exclusion (defined as 1 – Recall) are lower for the phone-based approach (53%) than for feasible alternatives (59%–78%).

**Table 1:** Performance of targeting mechanisms

		Targeting Novissi in rural Togo (2020 phone survey (*n* = 8,915))				Hypothetical nationwide programme (2018–2019 field survey (*n* = 4,171))			
		Spearman correlation	AUC	Accuracy	Precision and recall	Spearman correlation	AUC	Accuracy	Precision and recall
**Targeting methods considered by the Government of Togo in 2020**	Prefecture (admin-2 regions)	0.30 (0.017)	0.64 (0.008)	65% (0.87%)	39% (1.51%)	0.34 (0.017)	0.66 (0.008)	68% (0.74%)	45% (1.27%)
	Canton (admin-3 regions)	0.19 (0.019)	0.59 (0.009)	61% (0.78%)	33% (1.35%)	0.39 (0.016)	0.68 (0.008)	70% (0.71%)	48% (1.23%)
	Phone (expenditures)	0.13 (0.020)	0.57 (0.010)	60% (0.71%)	32% (1.23%)	0.26 (0.017)	0.63 (0.009)	65% (0.81%)	40% (1.40%)
	Phone (machine learning)	0.38 (0.017)	0.70 (0.009)	69% (0.87%)	47% (1.18%)	0.45 (0.015)	0.73 (0.007)	71% (0.74%)	50% (1.28%)
**Common alternative targeting methods that could not be implemented in Togo in 2020**	Asset index	0.10 (0.018)	0.55 (0.009)	60% (0.48%)	30% (0.83%)	0.51 (0.014)	0.75 (0.007)	74% (0.69%)	54% (1.19%)
	PPI	Data not available				0.63 (0.011)	0.81 (0.006)	77% (0.73%)	60% (1.25%)
	PMT	Data not available				0.72 (0.009)	0.85 (0.005)	78% (0.70%)	63% (1.20%)
**Additional counterfactual targeting methods that were feasible in Togo in 2020**	Random	0.00 (0.021)	0.50 (0.082)	59% (0.74%)	30% (0.26%)	0.00 (0.019)	0.50 (0.010)	59% (0.79%)	29% (1.36%)
	Occupation (as implemented)	−0.11 (0.019)	0.45 (0.007)	55% (0.62%)	22% (1.07%)	−0.09 (0.019)	0.46 (0.095)	56% (0.53%)	24% (0.91%)
	Occupation (optimally designed)	0.25 (0.016)	0.61 (0.008)	66% (0.58%)	41% (1.00%)	0.41 (0.016)	0.69 (0.008)	72% (0.72%)	52% (1.25%)

Targeting performance using mobile phone data and machine learning compared with counterfactual targeting strategies. The ‘true poor’ are those who, according to survey data, are in the poorest 29% of the population (the 29% threshold reflects the budget constraint of the rural Novissi expansion). Columns 3 to 6 evaluate targeting with a 2020 phone survey representative of subscribers in Togo’s 100 poorest cantons, using a PMT as ground truth for poverty, as consumption data were not collected. Columns 7 to 10 evaluate targeting using nationally representative household survey data collected in 2018–2019, using consumption as a ground truth. The top set of rows compares the phone-based PMT to alternative targeting methods that the Government of Togo considered prior to expanding Novissi to rural areas. The middle rows show the performance of targeting methods that are commonly implemented but were unfeasible in Togo at the time. The bottom rows indicate the performance of other targeting methods the government could have used. Accuracy, precision and recall are evaluated by the extent to which they reach the poorest 29% (by construction, precision and recall are equal in this simulation and are equal to 1 − exclusion error). Parentheses show s.d. produced from 1,000 bootstrap simulations.

Similarly, phone-based targeting outperforms most feasible methods when we simulate the targeting of a hypothetical national anti-poverty programme (Fig. 1b andTable 1, columns 7 to 10). Here, the phone-based approach is more effective at prioritizing the poor (AUC = 0.73) than geography-based alternatives (AUC = 0.66–0.68), and similarly leads to lower exclusion errors (50%) than most feasible alternatives (52%–76%). One exception in this hypothetical programme is occupation-based targeting: whereas the Novissi programme’s original criteria of targeting informal workers would not scale well to a national programme (76% exclusion errors), an alternative ‘optimal’ occupation-based approach that we develop (Methods, ‘Experimental design’)—which assigns all transfers to the poorest occupational category (agricultural workers)—slightly outperforms phone-based targeting (48% exclusion errors).

Together, the results in Table 1 indicate that the phone-based targeting approach was more effective in the actual rural Novissi programme than it would be in a hypothetical nationwide programme. Our analysis suggests that the benefits of phone-based targeting are greatest when the population under consideration is more homogeneous, and when there is less variation in other factors (such as place of residence) that are used in more traditional approaches to targeting (Methods, ‘Targeting methods and counterfactuals’). For instance, when we restrict the simulation of the hypothetical national programme to households in rural areas, the gains from phone-based targeting increase (Supplementary Table 1).

We also find that the performance benefits of phone-based targeting increase as programmes seek to target the most extreme poor. This increase can be seen by comparing Table 1, where targeting performance is measured by how many of the poorest 29% receive benefits, to Extended Data Table 1, which measures whether households below the extreme poverty line (US$1.43 per capita daily consumption) receive benefits, and Extended Data Table 2, which measures whether households below the poverty line (US$1.90 per capita daily consumption) receive benefits. Although all targeting methods perform better at targeting the extreme poor, the differential between the phone-based approach and other methods is greater when the consumption threshold is lower. (In this analysis, the wealth distribution of the underlying population is important: as more than half of the Togolese population is below the poverty line, the targeting methods are attempting to differentiate between different gradations of poverty. Just as precision increases as the target population grows—that is, from Table 1 to Extended Data Table 1 to Extended Data Table 2—results may differ in contexts where the target population is much smaller.)

The phone-based approach that we develop relies heavily on machine learning to construct a poverty score for each mobile subscriber, where eligibility is a complex function of how the subscriber uses their phone (Extended Data Table 3). We also consider an alternative approach that does not use machine learning, but instead simply targets mobile phone subscribers with the lowest mobile phone expenditures over the preceding months (Methods, ‘Parsimonious phone expenditure method’). We find that this ‘phone expenditure’ method (AUC = 0.57 for rural Novissi and 0.63 in for the hypothetical national programme; Table 1) performs substantially worse than the machine-learning-based model (AUC = 0.70 for rural Novissi and 0.73 for the hypothetical national programme). Although the phone expenditure model requires much less data and may be easier to implement, this parsimony increases targeting errors, and may also introduce scope for strategic ‘gaming’ if used repeatedly over time.

An important factor in the success of the machine-learning model is the fact that it was trained on representative survey data collected immediately before the programme’s expansion. Since an individual’s poverty status can change over time, and since the best phone-based predictors of wealth may also change, a model trained in one year or season may not perform well if applied in a different year or season. In Togo, we find that when the machine-learning model or the mobile phone data are roughly 18 months out of date, predictive accuracy decreases by 4–6% and precision drops by 10–14% (Extended Data Table 4 and Methods, ‘Temporal stability of results’). These losses are nearly as large as the gains that phone-based targeting provides over geographic targeting—a finding that underscores the importance of training the model with current and representative data.

We also compare the phone-based approach to alternative targeting approaches that require a recent and comprehensive social registry. Although the Government of Togo did not have such a registry, this comparison helps situate this method relative to other methods commonly used by development researchers and policymakers. These results, shown in Table 1, can only be simulated using the national in-person survey, since the phone survey did not collect consumption data. The results are more ambiguous: the phone-based approach (AUC = 0.70–0.73) is approximately as accurate as targeting using an asset-based wealth index (AUC = 0.55–0.75), but less accurate than using a poverty probability index (AUC = 0.81) or a perfectly calibrated PMT (AUC = 0.85) (see Methods, ‘Survey data’ for the differences between these indices). We note, however, that the performance of the ‘perfectly calibrated’ PMT may substantially overestimate the performance of a real-world PMT, which declines steadily over time since calibration^27,29^ (Methods, ‘Targeting methods and counterfactuals’).

## Social welfare and fairness

**Fig. 2:**
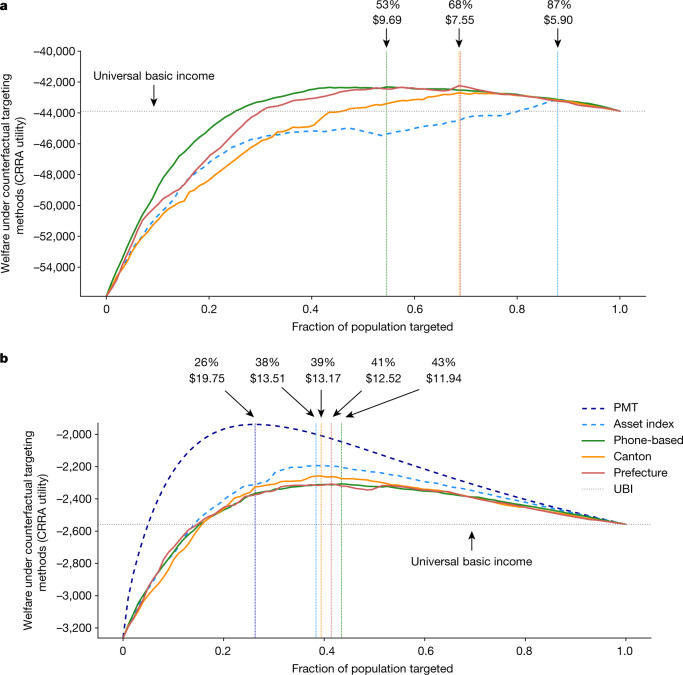
Welfare analysis of different targeting mechanisms. Aggregate social welfare is calculated (assuming CRRA utility) under counterfactual targeting approaches. We assume a fixed budget of US$4 million and a population of 154,238, with an equal transfer size for all beneficiaries. Utility curves for feasible targeting mechanisms are shown in solid lines; infeasible targeting mechanisms are shown in dashed lines. The horizontal dotted line indicates total social welfare for a universal basic income programme that provides (very small) transfers to the entire population; vertical dotted lines indicate the targeting threshold and associated transfer size that maximizes social welfare for each targeting mechanism. **a**, **b**, Targeting is evaluated for the Novissi anti-poverty programme in Togo’s 100 poorest cantons (**a**) and a hypothetical nationwide anti-poverty programme (**b**).

Improvements in targeting performance translate to an increase in social welfare. Using the constant relative risk aversion (CRRA) utility function, we calculate aggregate welfare under the phone-based approach and each of the counterfactual targeting approaches. Under the CRRA assumptions, individual utility is a concave function of consumption. By assuming a fixed budget—which we fix at a size analogous to that of the Novissi rural aid programme, which had a budget of US$4 million to distribute among 154,238 programme registrants—and equal transfer sizes to all beneficiaries, we simulate the distribution of benefits among eligible individuals at counterfactual targeting thresholds to construct social welfare curves for each targeting method. This social welfare analysis also allows us to identify the optimal beneficiary share and corresponding transfer size. Fig. 2 shows the utility curves for each of the targeting methods simulated, separately for the two populations. Note that phone-based targeting, geographic blanketing and an asset-based wealth index all achieve approximately the same maximum utility in the hypothetical national programme, but phone-based targeting dominates in the rural Novissi programme. Also note that all targeting methods outperform a universal basic income scheme if the beneficiary share and transfer size is well-calibrated.

**Fig. 3:**
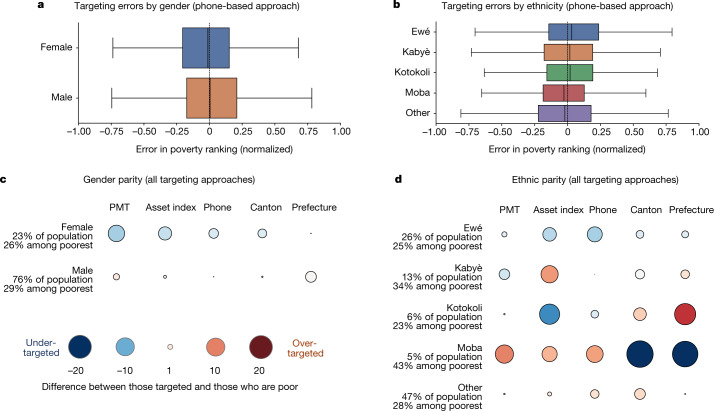
Fairness of targeting for different demographic subgroups. **a**, **b**, Distributions of differences between ranking according to predicted wealth from the phone-based approach and ranking according to true wealth (using the 2018–2019 field survey; *n* = 4,171), dissagregated by gender (**a**) and ethnicity (**b**). Boxes show the 25th and 75th percentiles, whiskers show the minimum and maximum, and the centre line shows the median of the distribution. Left-skewed bars indicate groups that are consistently under-ranked; right-skewed bars indicate groups that are consistently over-ranked. **c**, **d**, Evaluation of demographic parity across subgroups by comparing the proportion of a subgroup targeted under counterfactual approaches to the proportion of the subgroup that falls into the poorest 29% of the population (using the 2018–2019 field survey; *n* = 4,171), disaggregated by gender (**c**) and ethnicity (**d**). Bubbles show the percentage point difference between the proportion of the subgroup that is targeted and the proportion that is poor according to ground-truth data. Large red bubbles indicate groups that are over-targeted; large blue bubbles indicate groups that are under-targeted.

**Extended Data Fig. 2:**
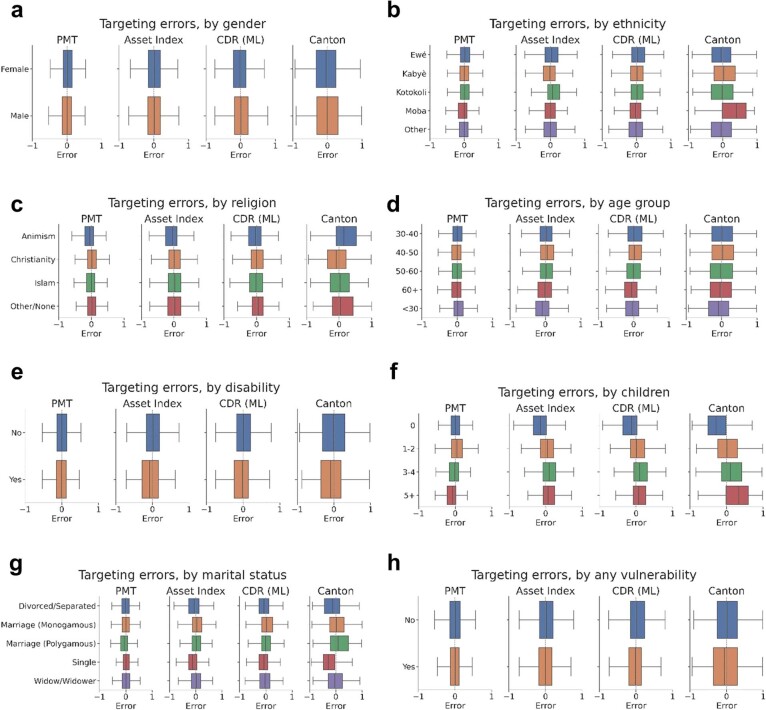
Fairness with normalized rank residuals. Boxplots showing distributions of normalized rank residuals (see Methods, ‘Fairness’) aggregated by subgroup, using the 2018–2019 field survey dataset (*N* = 4,171). Boxes show the 25^th^ and 75^th^ percentiles, and the centre line shows the median of the distribution. Left-shifted boxes indicate groups that are consistently under-ranked by a given targeting mechanism, right-shifted boxes indicate groups that are consistently over-ranked by a given targeting mechanism.

**Extended Data Fig. 3:**
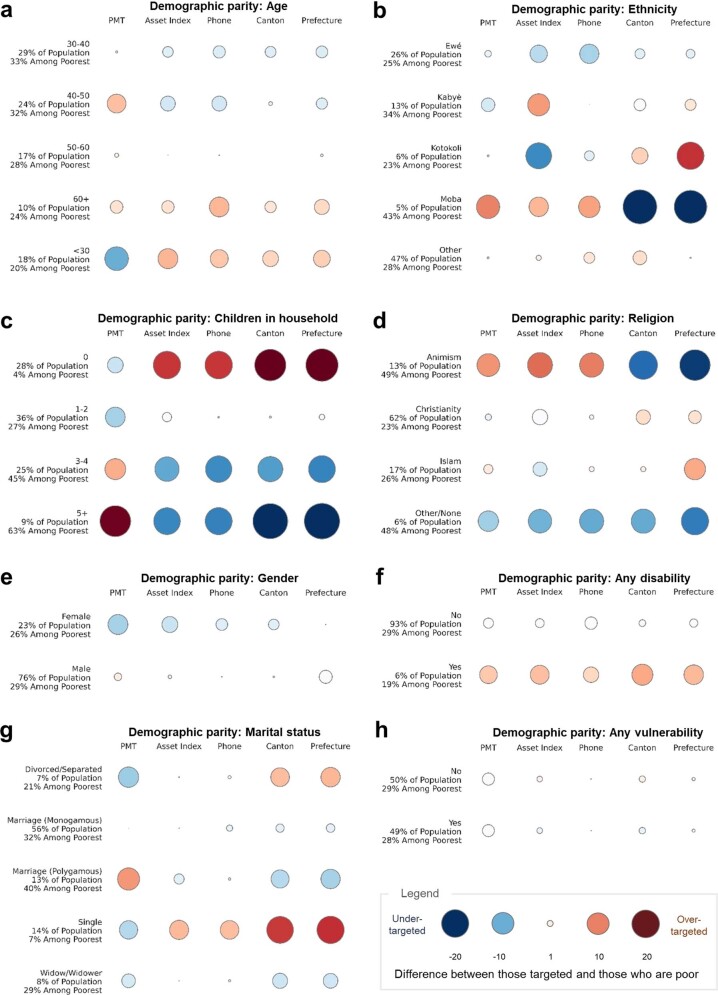
Fairness with demographic parity. We evaluate demographic parity across subgroups by comparing the proportion of a subgroup targeted under counterfactual approaches to the proportion of the subgroup that falls into the poorest 29% of the population (using data from the 2018–2019 field survey matched to CDR, *N* = 4,171). Bubbles show the percentage point difference between the proportion of the subgroup that is targeted and the proportion that is poor according to ground-truth data. Large red bubbles indicate groups that are over-targeted; large blue bubbles indicate groups that are under-targeted.

These utilitarian welfare gains suggest that society as a whole will benefit from improved targeting, but do not imply that all subgroups of the population will benefit equally. Indeed, there is growing concern that algorithmic decision making can unfairly discriminate against vulnerable groups^31–33^. To address these concerns in the context of the Novissi programme, we audit the fairness of each targeting method across a set of potentially sensitive characteristics, while noting that notions of fairness and parity are contested and often in tension^34^. Figure 3a shows, as an example, that the phone-based approach does not cause women to be systematically more likely to be incorrectly excluded by the targeting mechanism from receiving benefits than men (see also Methods, ‘Fairness’). Similarly, the phone-based approach does not create significant exclusion errors for specific ethnic groups (Fig. 3b), religions, age groups or types of household, though there are small differences in targeting accuracy between groups (Extended Data Fig. 2). We also compare the fairness of the phone-based approach to several other targeting approaches by evaluating each method’s demographic parity—that is, the extent to which each method under- or over-targets specific demographic subgroups relative to that group’s true poverty rate (Fig. 3c, d, Extended Data Fig. 3). Overall, we find that none of the targeting methods analysed naively achieves perfect parity across subgroups; a phenomenon referred to as ‘no fairness through unawareness’^35^. The largest parity differences occur with geographic targeting methods.

## Exclusions and limitations

This novel approach to targeting requires careful consideration of the ways in which individuals can be incorrectly excluded from receiving programme benefits (Methods, ‘Programme exclusions’). Our analysis highlights six main sources of exclusion errors for the expansion of Novissi (Table 2): (1) beneficiaries must have a SIM card and access to a mobile phone (field survey data from 2018–2019 indicate that 65% of adults and 85% of households have a phone; see also Supplementary Fig. 3); (2) they must have used their SIM card recently, in order to generate a poverty score (between 72% and 97% of programme registrants); (3) they must be a registered voter (roughly 87% of adults); (4) they must self-target and attempt to register (roughly 40% of eligible individuals attempted); (5) they must succeed in registering, which requires basic reading and digital literacy (72% succeed); and (6) they must be successfully identified as eligible by the machine-learning algorithm (47% recall; Table 1). Many of these sources of possible exclusion overlap; Extended Data Table 5 thus estimates, on the basis of the 2020 phone survey, the extent to which each successive step in registration creates additional exclusions. These results highlight the fact that algorithmic targeting errors are an important source of programme exclusion, but that real-world programmes also face structural and environmental constraints to inclusion.

**Table 2:** Sources of exclusion from rural Novissi benefits

Exclusion source	Proportion included	Data and calculations
Voter ID possession	83–98%	According to administrative data, 3,633,898 individuals are registered to vote in Togo. The electoral commission of Togo reports that this corresponds to 86.6% of eligible adults^44^. The total adult population in Togo is not certain (the last census was in 2011), but Togo’s national statistical agency (https://inseed.tg/) estimates that there are 3,715,318 adults in Togo; the United Nations estimates 4.4 million adults^45^. These imply a voter ID penetration rate of either 82.6% or 97.8%, respectively.
SIM card and mobile phone access	50–85%	65% of individuals interviewed in the 2018–2019 field survey (*n* = 6,171) reported owning a phone; 85% of individuals were in a household with one or more phones. Rural penetration is lower (50% of individuals and 77% of households), as is penetration among women (53% for women vs 79% for men; in rural areas, it is 33% for women and 71% for men) (Supplementary Fig. 3). Phone penetration in Togo probably increased between the field survey (2018–2019) and the Novissi expansion (October 2020); the Togolese government estimates 82% SIM card penetration^44^.
Past mobile phone use	72–97%	Poverty estimates were constructed only for subscribers who placed at least one outgoing transaction between March and September 2020. In a typical month, 2.5% of all phone numbers are newly registered (Supplementary Fig. 6), so with a one-month gap between poverty inference and programme registration we would expect 95–97% of registrations to be associated with a poverty score. However, 27% of all Novissi registrations (November–December 2020) did not match to CDR, probably owing to new SIM purchases or registration on infrequently used SIMs (Methods, ‘Programme exclusions’).
Programme awareness	35–46%	245,454 unique subscribers attempted to register for the rural Novissi programme. The total voting population of eligible areas is 528,562, implying a maximum registration rate of 46.44%. However, not all 245,454 registration attempts were made by people living in eligible areas; examining administrative data on home location from successful registrations, we estimate that 87% of registration attempts came from eligible areas, implying an attempted registration rate of 40.40%. An alternative way to estimate attempted registration rates involves comparing the number of registration attempts made by phones below the poverty threshold (69,753) with our estimate of the number of voters in eligible cantons below the poverty threshold based on inferred home locations from mobile phone data (174,425; see Supplementary Methods section 4 for details), which implies an attempted registration rate of 34.79% after scaling by 87% (to account for registrations that came from outside of eligible areas).
Registration challenges	72%	Registration for the Novissi programme requires entering basic information into a USSD (phone-based) platform. According to programme administrative data, of the 245,454 subscribers who attempted registration, 176,517 (71.95%) eventually succeeded. The average registration required four attempts.
Targeting errors	47%	Based on the estimates from our targeting simulations using the 2020 phone survey (Table 1), the exclusion error rate of the phone-based targeting algorithm is 53%.

We use multiple sources of administrative data, survey data and government sources to estimate the extent to which different elements of the design of the Novissi programme may have led to errors of exclusion. Eligibility requirements for Novissi included: a valid voter ID (as a unique identifier and for home location), access to a mobile phone (to fill the register using the unstructured supplementary service data (USSD) platform), past mobile network transactions (to estimate poverty from mobile network behaviour), programme awareness (to know that the programme exists and to attempt to register), ability to register via the USSD platform (which requires basic digital literacy), as well as targeting errors from the phone-based machine-learning algorithm. This table calculates sources of exclusion as though they were all independent; Extended Data Table 5 uses survey data to calculate overlaps in exclusions.

More broadly, our analysis shows how non-traditional big data and machine learning can improve the targeting of humanitarian assistance. Beyond the gains in targeting performance, a key advantage of this approach is that it can be deployed quickly and responsively. In Togo, the government’s objective was to deliver benefits to the poorest people in the country, so our efforts focused on training a machine-learning model to target the poor. In other settings, such as following natural disasters, the people most impacted by adverse events may not be the poorest^36^. With high-frequency phone data available in near real-time, related techniques might be used to more dynamically prioritize the people with the greatest need. For example, it may be possible to train a machine-learning algorithm to identify people whose consumption fell by the greatest amount, based on changes in patterns of phone use following a crisis. Another possibility would be to simply use location information from mobile phone data to prioritize people who are likely to live in impacted regions (Methods, ‘Location-based targeting’).

It is important to emphasize that our phone-based approach is far from perfect, and may lead to important errors of both exclusion and inclusion. There are also practical limitations to this approach, for instance regarding data access and privacy^37–43^; several such considerations are addressed in Supplementary Discussion, section 2. Moreover, our results do not imply that mobile-phone-based targeting should replace traditional approaches reliant on proxy means tests or community-based targeting. Rather, these methods provide a rapid and cost-effective supplement that may be most useful in crisis settings or in contexts where traditional data sources are incomplete or out of date. We believe that future work should explore how real-time data sources, such as the phone data used by Novissi, can be best combined with more traditional field-based measurements, so that these complementary data sources can be best integrated in the design of inclusive systems for social protection^19^.

## Methods

### The COVID-19 pandemic in Togo

Togo is a small country with a population of roughly 8 million in West Africa. More than 50% of the population lives below the international poverty line. Shortly after the first COVID-19 cases were confirmed in Togo in early March 2020, the government imposed economic lockdown orders to prevent the spread of the disease. These lockdowns forced many Togolese to stop working, raising concerns about the potential for rising food insecurity (Supplementary Fig. 1).

On April 8, 2020, the government launched the Novissi programme (Novissi means solidarity in the Ewé language). According to the Togolese minister C. Lawson, Novissi “was built and designed in order to help those people who are the most vulnerable population and the most impacted by the anti-COVID measures”^46^. Novissi was initially designed to provide benefits to informal workers in Greater Lomé, the large metropolitan area surrounding the capital city where the lockdown orders were initially focused. The rationale for targeting informal workers was that they were more likely to be vulnerable and more likely to be affected by the lockdown orders.

To determine eligibility for Novissi, the government relied upon a national voter registry that was updated in late 2019, in which individuals indicated their home location and occupation. At the time, the voter registry contained 3,633,898 entries, which the electoral commission reports is equivalent to 87% of the total adult population (see Table 2 for details).

Receiving Novissi benefits required that individuals register by dialing in to the Novissi unstructured supplementary service data (USSD) platform from a mobile phone. Thus, registration initially required (1) a valid and unique voter ID linked to an eligible occupation from an eligible location; (2) a valid SIM card, and (3) access to a mobile phone. A smartphone was not required for registration; the USSD platform was accessible from a basic phone. Since phone sharing is common in Togo, multiple SIM cards could be registered through a single phone (so long as each SIM was then linked to a valid voter ID). See ‘Programme exclusions’ for a discussion of the extent to which voter and phone requirement may have led to programme exclusions.

Eligible female beneficiaries were then paid 12,250 FCFA (US$22.50) per month; men received 10,500 FCFA (US$20) per month. The payments were disbursed in two bi-weekly installments, for three months, using existing mobile money infrastructure managed by the country’s two mobile network operators. The system was designed to be 100% digital, so that registration, eligibility determination and payment could all be accomplished without face-to-face contact. Novissi was promoted actively through radio advertisements and community leaders, and 4.4 million registration attempts were reported on the day the programme launched. In this first phase of Novissi, which focused on Greater Lomé, roughly 510,000 beneficiaries received payments.

During the summer of 2020, in response to localized outbreaks of COVID-19, the government piloted an expansion of Novissi based on geographic targeting. In this geographically targeted expansion, all individuals registered to vote in the Soudou canton were made eligible for Novissi benefits. The geographic targeting was determined primarily by public health considerations, and not by poverty rates. In total, roughly 5,800 beneficiaries were paid through this geographically targeted programme.

Our analysis focuses on a second phase of Novissi, which was initiated after the Novissi programme in Greater Lomé had terminated. Specifically, in partnership with the non-governmental oganization GiveDirectly, the government wished to expand Novissi eligibility to the rural poor. The policy mandate from the government was to (1) prioritize benefits to people living in Togo’s 100 poorest cantons (of the 397 cantons nationally), where the number 100 was selected by the government in order to balance the desire to focus on the poorest villages, without focusing excessively on specific regions; and (2) prioritize the poorest individuals in those 100 cantons.

During the second phase of Novissi, registration and enrolment used several of the same steps described above: individuals were required to have a voter ID registered in one of the 100 poorest cantons, and they had to self-register using a mobile phone with a unique SIM card. However, the individual’s occupation was not used to determine eligibility; instead, the estimated wealth of the individual, based on the machine-learning methods described in this paper, was used to limit eligibility to the estimated poorest subscribers in those 100 cantons.

### Data sources

#### Survey data

Our core analysis relies heavily on two surveys conducted by Togo’s Institut National de la Statistique et des Études Economiques et Démographiques (INSEED). The first survey, which is nationally representative, was conducted in the field in 2018 and 2019 (*n* = 6,171). The second survey was conducted over the phone in September 2020, and is representative of mobile network subscribers inferred to be living in rural cantons eligible for Novissi aid (*n* = 8,915). We use these two different survey datasets because neither dataset is sufficient by itself for the analysis we require: the 2020 survey did not collect consumption data, which is important for evaluating certain counterfactuals; the 2018–19 survey is representative only at the prefecture level, and only surveyed a small number of households in the 100 poorest cantons that were eligible for Novissi. (We had planned to conduct a large in-person survey in early 2021 that would provide the single point of focus for this paper, but were forced to postpone the survey indefinitely owing to a resurgence in COVID-19.)

##### 2018–2019 field survey

Our first survey dataset was obtained from a nationally representative household survey. Specifically, 540 enumeration areas (EAs) were drawn at random from Togo’s approximately 6,000 EAs, with weight proportional to the size of the EA in the last national census (conducted in 2011). Twelve households were then drawn at random from each of the selected EAs to be interviewed, for a total of 6,172 households. Surveys, which lasted about 3 h, were conducted in two waves, with the first wave between October and December 2018 and the second wave between April and June 2019. We removed one observation that is missing consumption expenditure and asset data, leaving 6,171 observations. Interviews took place with the head of household when possible, and alternatively with the most knowledgeable adult present. Answers were recorded by enumerators on tablets using SurveyCTO software.

As part of the survey’s recontact protocol, phone numbers were requested from a representative of each household; 4,618 households (75%) of households are matched to a phone number. The data do not include an identifier for which member of the household the phone number belongs to. A total of 4,171 households have phone numbers that contain at least one transaction in our mobile phone transaction logs in the three months prior to their survey date (90% of households with phone numbers), leading to a matched survey–mobile phone dataset with *n* = 4,171. Note that this matched dataset is not nationally representative or necessarily representative of mobile phone subscribers, as there is selection in which households and household members provide phone numbers.

##### 2020 phone survey

Our second survey dataset is obtained from a phone survey conducted over two weeks in September 2020. The survey lasted approximately 40 min, and covered demographics, asset ownership and well-being. Answers were recorded by enumerators on tablets using SurveyCTO software. Phone numbers for the 2020 phone survey were drawn from mobile phone transaction logs and the sample is representative of subscribers inferred based on their mobile phone data to be living in rural cantons eligible for Novissi aid (see Supplementary Methods, section 4). Note that because the sample is drawn based on inferred location, not all interviewees necessarily reside in an aid-eligible canton. The survey includes a question on canton of residence, and 68% of observations report living in a Novissi-eligible canton.

Of the phone numbers drawn, 35% responded, consented to the survey, and completed the entire survey. In total, after removing low-quality surveys and those missing poverty outcomes, the dataset contains 8,915 observations corresponding to individual subscribers. We reweight the survey for nonresponse using the same mobile phone features and machine-learning methods described in ‘Predicting poverty from phone data’. Our sample weights consist of the inverse of the draw probability and the inverse of the predicted probability of response. More details on the content of the 2020 phone survey, the sampling procedure, and the reweighting procedure are available in Supplementary Methods, section 5.

#### Construction of poverty outcomes

We construct four poverty outcomes from the survey data: consumption expenditure (captured in the 2018–2019 field survey only), an asset-based wealth index, a poverty probability index (PPI), and a PMT.

#### Consumption expenditure

The consumption expenditure outcome is only available in the dataset from the 2018–2019 field survey. Disaggregated expenditures for more than 200 food and non-food items are elicited in each household interview. The consumption aggregate is then adjusted for a price index calculated at the prefecture level. The final outcome measure is per capita adult equivalent household consumption expenditure, which we transform to US$ per day.

#### Asset index

We calculate a principal component analysis (PCA) asset index for households in the 2018–2019 field survey and for the households associated with individuals interviewed in the 2020 phone survey. Asset indices are constructed with a PCA. The asset index is constructed from 24 underlying binary asset variables in the 2018–2019 field survey and 10 underlying binary asset variables in the 2020 phone survey. The asset indices for the two surveys are constructed independently, from different sets of assets, and therefore do not share a basis vector. The basis vector for each index is shown in Supplementary Table 2. The asset index explains 31.50% of the variance in asset ownership in the 2018–2019 field survey, and 53.45% of the variance in asset ownership in the 2020 phone survey. However, the variance explained in the two indices should not be directly compared since there are far fewer assets recorded in the 2020 phone survey than in the 2018–2019 field survey. We also note that the asset index for the 2020 phone survey dataset is dominated by variation in ownership of three assets (toilet, radio and motorcycle; see Supplementary Table 2) and is therefore considerably less smooth than the asset index in the 2018–2019 phone survey dataset.

#### PPI

We use the scorecard for the current PPI used by Innovations for Poverty Action (https://www.povertyindex.org/country/togo). The index is calibrated based on a nationally representative survey conducted by INSEED in 2015 (*n* = 2,335). ‘Poverty probability’ is scored based on ten household questions, including region of residence, education of adults and children, asset ownership, and consumption of sugar. We calculate the PPI only for households in the 2018–2019 field survey, as the data necessary for all components were not collected in the 2020 phone survey.

#### PMT

Using the data from the 2018–2019 field survey, we follow a stepwise forward selection process to select the 12 asset and demographic variables that are jointly most predictive of per capita household consumption (see Supplementary Fig. 4, Supplementary Methods, section 3 for details). We use these variables to construct a consistent PMT for the 2018–2019 field survey and the 2020 phone survey. Following recent literature, we use a regularized linear model (Ridge regression) rather than a simple linear regression to maximize out-of-sample accuracy^30,33^. For the 2018–2019 field survey, PMT consumption estimates are produced out-of-sample over tenfold cross validation. For the 2020 phone survey, we train the Ridge regression on the entire 2018–2019 field survey sample and use the fitted model to produce PMT consumption estimates for each phone survey observation. Over tenfold cross validation, the PMT explains 48.35% of the variance in log-transformed consumption expenditure in the 2018–2019 field survey. This explanatory power is similar to that of other national-scale PMTs reported in Indonesia, Peru and Jamaica^3,22,26^ (41%–66%). The weights for the PMT are included in Supplementary Table 3. As they are trained to predict consumption, PMT consumption estimates can be interpreted as estimated US$ per day.

#### Rural-specific PMT

We follow another stepwise forward selection process using the 2018–2019 field survey restricted to households in rural areas (*n* = 3,895) to create a PMT specific to rural areas with 12 components. The weights for the rural-specific PMT are shown in Supplementary Table 4. Over tenfold cross-validation the rural-specific PMT explains 17% of the variation in log-transformed consumption expenditure in the 2018–2019 field survey restricted to rural areas. We note that this explanatory power is substantially lower than that of other rural-specific PMTs evaluated in past work in Jamaica and Burkina Faso^47,48^ (36%–45%). We produce out-of-sample values for the rural-specific PMT over cross validation for the 2018–2019 field survey, and use the fitted model to produce values for the 2020 phone survey. We mean-impute the rural-specific PMT for observations that do not have all necessary components in the 2020 phone survey dataset (*n* = 18). The correlation between the rural-specific PMT and general PMT is 0.75 in the 2018–2019 survey dataset restricted to rural areas, and 0.76 in the 2020 phone survey dataset.

##### Construction of occupation categories

We use self-reported occupation (of the household head for the 2018–2019 field survey, and of the respondent for the 2020 phone survey) to categorize occupations and later simulate occupation-based targeting. We first classify each of the self-reported occupations according to the occupation categories in the Novissi registry. We identify which of these categories are informal (in the Novissi registry, more than 2,000 unique occupations are considered informal—some of the most common ones are vendors, hairdressers, taxi drivers, tailors, construction workers and the unemployed). We further classify occupations in 10 broad categories according to the Afrostat system (https://www.afristat.org/nomenclatures/). Supplementary Table 5 records these categories, along with the proportion in each category in each of the two surveys and associated average consumption.

##### Summary statistics

Supplementary Table 6 presents summary statistics on each of the two surveys; for the 2018–2019 household survey, results are presented separately for households who provide phone numbers (further broken down into those with phones numbers that match to the mobile phone metadata and those whose phone numbers do not match), and those without phone numbers. Note that since phone numbers for the 2018–2019 household survey were collected for a recontact protocol, a household without a phone number could represent a household without a phone or one that refused to be contacted for further surveys. We find that households providing phone numbers (average consumption = US$2.56 per day) are less poor than households not providing them (average consumption = US$1.75 per day); among those associated with a phone number, households that do not match to mobile phone metadata (average consumption = US$2.21 per day) are poorer than those that do (average consumption = US$2.59 per day). These patterns are consistent with related work in Afghanistan in which phone numbers were collected for the purpose of matching to mobile phone metadata. That study found that households with phones were wealthier than those without, and households associated with a matched phone number were wealthier than those that did not match^19^.

Comparing summary statistics from the 2020 phone survey and 2018–2019 household survey, respondents to the 2020 survey tend to be poorer (average PMT = 1.62 verrss 2.10), younger (average age = 33 versus 44), and more predominantly male (23% women vs 28% women). These differences are not surprising given that the 2020 survey was conducted in rural areas whereas the 2018–2019 household survey was designed to be nationally representative.

#### Poverty maps

To simulate geographic targeting, we rely on poverty maps of Togo’s prefectures (admin-2 level, 40 prefectures) and cantons (admin-3 level, 397 cantons). In the 2018–2019 field survey, the latitude and longitude of each household were recorded by enumerators as part of the interview, so we map each observation to a prefecture and canton using the geographic coordinates. For the 2020 phone survey, we ask each respondent to report their prefecture and canton of residence.

##### Prefecture poverty map

INSEED completed a survey-based poverty mapping exercise in 2017. Specifically, a PMT was calibrated on a small consumption sample survey conducted in 2015 (*N* = 2,335). 26,902 households were then surveyed in the field over three weeks in 530 EAs, sampled to be representative at the prefecture level. The interview included questions on demographics, education, asset ownership, and household characteristics that made up the PMT. The calibrated PMT was then used to infer the ‘consumption’ of each household, and observations were aggregated to estimate the percentage of the population living under the Togo-specific poverty line of US$1.79 per day in each prefecture. Supplementary Fig. 5 shows the resulting poverty map. For validation, we evaluate the correlation between prefecture-level poverty rates from the poverty mapping exercise and average consumption in the 2018–2019 field survey. The Pearson correlation coefficient is −0.78, and the Spearman correlation coefficient is −0.70.

##### Canton poverty map

When COVID-19 first appeared in Togo in early 2020, it had been at least ten years since a household survey had been conducted in Togo that was representative at the canton level. Togo’s last census was conducted in 2011, but did not include information on income, consumption, or asset ownership. We therefore rely on recently-produced publicly available satellite-based estimates of poverty which use deep learning models trained on Demographic and Health Surveys (DHS) data from neighbouring countries to estimate the average relative wealth of each 2.4km tile in Togo^16^. We overlay the resulting tile-level wealth estimates with high-resolution estimates of population density inferred from satellite imagery^49^ to obtain population-weighted average wealth estimates for each canton, shown in Supplementary Fig. 5. As noted in ref. ^16^, the relative wealth measures are estimated with uncertainty. Thus, for validation, we evaluate the canton-level correlation between average wealth from the satellite-based poverty map and average consumption in the 2018–2019 field survey (though note that the latter survey is not representative at the canton level). The Pearson correlation coefficient is 0.57, and the Spearman correlation coefficient is 0.52.

#### Mobile phone metadata

We obtain mobile phone metadata (call detail records (CDR)) from Togo’s two mobile network operators for certain time periods in 2018–2021. We focus on three slices of mobile network data: October–December 2018, April–June 2019 and March–September 2020. The three-month periods in 2018 and 2019 are matched to households interviewed in the first and second wave of the field survey, respectively. The seven-month period in 2020 is matched to outcomes for individuals interviewed in the phone survey in September 2020. Summary statistics on network activity in these periods are shown in Supplementary Fig. 6.

Our CDR data contain the following information. Calls: caller phone number, recipient phone number, date and time of call, duration of call, ID of the cell tower through which the call is placed; SMS messages: sender phone number, recipient phone number, date and time of the message, ID of the antenna through which the message is sent; mobile data usage: phone number, date and time of transaction, amount of data consumed (upload and download combined); mobile money transactions: Sender phone number, recipient phone number (if peer-to-peer), date and time of the transaction, amount of transaction, and broad category of transaction type (cash in, cash out, peer-to-peer or bill pay).

##### October–December 2018 and April–June 2019 CDR

Between 1 October and 30 December 2018, there were a total of 4.84 million unique mobile network subscribers between the two mobile phone networks (where a subscriber is any phone number that places at least one call or SMS on a network). Between 1 April and 30 June 2019, there were a total of 4.89 million mobile network subscribers. We identify spammers on the network as any phone number that placed an average of over 100 calls or 100 SMS messages per day, and remove any transactions associated with these numbers from our dataset. We remove 232 spammers in the 2018 time period and 162 spammers in the 2019 time period. In the 2018–2019 CDR, we observe only calls, SMS messages, and mobile money transactions (we do not observe mobile data usage).

##### March–September 2020 CDR

For data between March 1 and September 30, 2020, we observe a total of 5.83 million mobile network subscribers (note that this subscriber population does not necessarily reflect a 19% increase in subscribers from 2018–2019, since the slice is seven months rather than three months and there is significant month-to-month churn in subscribers; during the 3-month period from July–September 2020 we observe 5.20 million unique subscribers, a 6% increase from the 2019 period). We identify spammers as described above, resulting in the removal of transactions associated with 107 spammers from the 2020 CDR dataset. In the 2020 CDR, we observe calls, SMS messages, mobile data usage, and mobile money transactions.

##### Featurization

For each subscriber observed on the network in each of the three time periods, we calculate a set of 857–1,042 ‘CDR features’ that describe aspects of the subscriber’s mobile phone behaviour. These include:

Call and SMS features. We use open-source library bandicoot^50^ to produce around 700 features relating to the calls and SMS messages each subscriber places and receives. These range from general statistics (for example, number of calls or SMS messages, or balance of incoming versus outgoing transactions), to social network characteristics (for example, number and diversity of contacts), to measures of mobility based on cell tower locations (for example, number of unique towers and radius of gyration).

Location features. Based on the locations of each of the cell towers in Togo, we calculate information about where each subscriber places their transactions. Specifically, we calculate the number and percentage of calls placed in each of Togo’s 40 prefectures, and the number of unique antennas, cantons, prefectures, and regions that each subscriber visits.

International transaction features. Using country codes associated with phone numbers, we calculate the number of outgoing international transactions, separately for calls and SMS messages. We also calculate the total time spent on outgoing international calls.

Mobile money features. For each of four variables relating to transaction size–transaction amount, percent of balance, balance before transaction, and balance after transaction–we calculate the mean, median, minimum, and maximum, separately for incoming and outgoing mobile money transactions. We also calculate the total transaction count for each subscriber (separately for incoming and outgoing) and the total number of unique mobile money contacts (separately for incoming and outgoing). We perform these calculations for all transactions together, as well as separately by transaction type (cash in, cash out, peer-to-peer, bill payments and other transactions).

Mobile data features. We calculate the total, mean, median, minimum, and maximum mobile data transaction for each subscriber, as well as the standard deviation in transaction size. We also calculate the total number of mobile data transactions and the number of unique days on which data is consumed. Note that mobile data features are only calculated for the 2020 CDR period, as our 2018–2019 CDR does not include mobile data records.

Operator. In our feature dataset we include a dummy variable for which of the two mobile network operators each subscriber is associated with.

##### Matching survey and CDR datasets

Using phone numbers collected in surveys, we match survey observations to CDR features. As noted in ‘Survey data’, there are 4,618 households in the 2018–2019 field survey that provide a phone number, of which 4,171 match to CDR (90% of households with phone numbers, and 68% of households overall). We match households surveyed in the first survey wave to features generated in the October–December 2018 CDR period, and households surveyed in the second survey wave to features generated in the April–June 2019 CDR period. To build intuition on the relationships between phone-related features and poverty, Supplementary Fig. 7 compares four CDR features for those above and below the poverty line in the 2018–2019 household survey. As the 2020 survey was sampled based on the CDR dataset, all 8,915 observations in the 2020 survey dataset are matched to CDR.

#### Data privacy concerns

The CDR data we obtained for each subscriber contain personally identifying information (PII) in the form of the subscriber’s phone number (it does not contain the individual’s name, address or other PII), as well as other potentially sensitive information such as data about the subscriber’s network and cell tower locations. To protect the confidentiality of these data, we pseudonymized the CDR prior to analysis by hash-encoding each phone number into a unique ID. The data are stored on secure university servers to which access is limited based on a data management plan approved by UC Berkeley’s Committee for the Protection of Human Subjects.

We obtained informed consent from all research subjects in the phone survey prior to matching CDR records to survey responses. However, there are still open concerns around the use of CDR by bad actors, particularly as even pseudonymized datasets can frequently be de-anonymized for a subset of observations^37,51^. Active research on applying the guarantees of differential privacy to CDR datasets and associated machine-learning models holds promise for balancing the utility of CDR data with privacy concerns^52,53^. For additional discussion of these considerations, see Supplementary Discussion, section 2.

### Predicting poverty from phone data

#### Machine-learning methods

We follow the machine-learning methods described in prior work^17–19^ to train models that predict poverty from CDR features. Specifically, we train a gradient boosting regressor with Microsoft’s LightGBM for the two matched survey-CDR datasets separately. We tune hyperparameters for the model over threefold cross validation, with parameters chosen from the following grid:

Winsorization of features: {No winsorization, 1% limit}

Minimum data in leaf: {10, 20, 50}

Number of leaves: {5, 10, 20}

Number of estimators: {20, 50, 100}

Learning rate: {0.05, 0.075, 0.1}

We train and evaluate the model over fivefold cross validation, with hyperparameters tuned independently on each fold, to obtain out-of-sample estimates of accuracy and out-of-sample predictions of poverty for each observation in our matched survey datasets. We then re-train the model on all survey data (for each of the two datasets separately), record feature importances (the total number of times a feature is split on over the entire forest), and use the final model to generate wealth predictions for every subscriber on the mobile phone network during the relevant time period.

We experiment with training models in this way for each of the relevant poverty outcomes: consumption expenditure, PMT, and asset index for the 2018–2019 field survey dataset and PMT and asset index for the 2020 phone survey dataset. Evaluations of model accuracy are found in Extended Data Table 6. The correlation between the phone-based poverty predictions and a traditional PMT is 0.41, as trained and evaluated on the 2020 phone survey dataset (Extended Data Table 6, panel c). When trained and evaluated using the national 2018–2019 household survey with consumption data, the correlation between the phone-based poverty predictions and consumption is 0.46 (Extended Data Table 6, panel a).

##### Feature importances

Feature importances for each model are presented in Extended Data Table 3. We note that in examining the feature importances, location-related features (number and percent of calls placed in each prefecture of the country) are very important. The correlation between phone-based poverty predictions using only these location features and a standard PMT is 0.35 when trained and evaluated with the 2020 phone survey (versus 0.41 using all features). When trained and evaluated with the 2018–2019 field survey, the correlation between location-only phone-based poverty predictions and consumption is 0.42 (versus 0.46 when using all features). Given the relative importance of location features, we provide more in-depth analysis of the role of geography in phone-based targeting approaches in ‘Location-based targeting’. Other important features in the full phone-based poverty scores relate to nighttime calling behaviour, mobile data usage and mobile money usage.

##### Aggregate validation of CDR-based poverty estimates

Our machine-learning models use cross-validation to help limit the potential that the predictions are overfit to the specific surveys on which they are trained (and on which they are later evaluated in the targeting simulations). To provide a more independent test of the validity of the CDR-based estimates, we compare regional aggregates of wealth based on the CDR model to regional estimates of wealth based on household survey data. In this exercise, we predict the consumption of roughly 5 million subscribers in Togo using the machine-learning model trained to predict consumption using the 2018–2019 national household survey, then calculate the average consumption of each prefecture and canton (where each subscribers’ home location is inferred from CDR using standard methods described in Supplementary Methods, section 4).

Results, shown in Supplementary Fig. 8, indicate that the CDR-based estimates of regional poverty correlate with survey-based estimates of regional poverty. At the prefecture level, the Pearson and Spearman correlations of CDR-based consumption with survey-based consumption are 0.92 and 0.83, respectively; the correlations with the proportion of each prefecture living in poverty are −0.76 and −0.74. At the canton level, comparing the CDR-based estimates to the satellite-inferred canton poverty map from Supplementary Fig. 5, we find Pearson correlation = 0.84 and Spearman correlation = 0.68; compared to the average canton consumption in the 2018–19 field survey, Pearson correlation = 0.57 and Spearman correlation = 0.59. These correlations are toward the lower end of the range of correlations observed in prior efforts to estimate regional poverty with CDR^14,15,17^.

#### Parsimonious phone expenditure method

In addition to the machine-learning method for wealth prediction described above, we are interested in the performance of an intuitive, parsimonious method for approximating poverty with CDR. We focus on a measure of ‘phone expenditure’ on the basis of costs of all calls placed and SMS messages sent by each subscriber. We apply standard rates for calls and SMS messages in Togo: 30 CFA (US$0.06) to send an SMS message and 50 CFA (US$0.09) per minute of call time. (These prices represent a typical Togolese phone plan, though there is considerable diversity in special promotions and friends-and-family plans available from Togo’s two mobile phone operators, Moov and Togocom.) We use these prices to infer the (approximate) amount spent by each subscriber from their outgoing mobile phone transaction logs. We find that the phone expenditures method is ssubstantially less accurate than the machine-learning-based method, with a correlation of 0.13 with both the 2020 phone survey PMT and the 2018–2019 household survey’s consumption measure (Extended Data Table 6a, c).

### Targeting evaluations

#### Experimental design

We simulate phone-based and counterfactual targeting methods for reaching the poorest individuals in Togo, using the two survey datasets described in ‘Survey Data.’ Specifically, for each dataset, we simulate providing benefits to the poorest 29% of observations in the dataset based on a suite of counterfactual targeting options (with sample weights applied), and compare the population targeted to the population that is ‘truly poor’, where ground truth poverty is determined using two different measurements. With the 2018–2019 in-person survey dataset, our main ground-truth wealth measure is based on consumption expenditure: we evaluate how well proxy measures of poverty reach those with the lowest consumption. For the 2020 phone survey dataset, our main ground-truth wealth measure is based on the PMT described in the section ‘Survey data’ (this is necessary because consumption information was not collected in the phone survey).

Our main targeting evaluations simulate targeting 29% of individuals because the Novissi programme had sufficient funds to target 29% of registrants in eligible cantons. The 29th percentile corresponds to a consumption threshold of US$1.17 per day in the 2018–2019 field survey dataset, and a PMT threshold of US$1.18 per day in the 2020 phone survey dataset. Our analysis shows how accurately each targeting method reaches the 29% truly poorest (Table 1), those below the extreme poverty line, defined as three-quarters of the poverty line, or US$1.43 per day (Extended Data Table 1), and those below the international poverty line of US$1.90 per day (Extended Data Table 2).

Our evaluations are designed to measure how effectively several different targeting methods, described below, are at reaching the poorest individual mobile phone owners in each of the two survey populations. We focus on individuals rather than households because the Novissi programme was designed and paid as an individual benefit. While social assistance programmes in other countries typically consider the household to be the unit of analysis that determines programme eligibility, there is no notion of a household unit in the Novissi programme (in part because the government does not possess data that links individuals to households). See Supplementary Discussion section 2 for additional discussion of the implications of individual versus household-level analysis.

Likewise, our focus on mobile phone owners reflects the fact that the Novissi system in Togo distributed payments via mobile money; as such, anyone without access to a phone could not receive benefits irrespective of the targeting method—see ‘Programme exclusions’ for a discussion of exclusion errors resulting from this constraint. In practice, this constraint only affects the analysis using the 2018–2019 in-person survey, where 4,171 of 6,171 respondents provided an active phone number. For analysis using the 2020 phone survey, we include all respondents, as every respondent had access to a phone. Future work could compare phone-based targeting to counterfactual targeting methods that could be implemented in-person, and thus account for exclusion errors resulting from phone ownership.

#### Targeting methods and counterfactuals

Our evaluations use the two survey datasets to measure the performance of three targeting methods that were feasible when implementing the Novissi programme: geographic blanketing (targeting everyone in certain geographies), occupation-based targeting (targeting everyone in certain occupation categories), and phone-based targeting. The location of subscribers targeted by each of these methods, in both the rural Novissi programme and the hypothetical national programme, are shown in Supplementary Fig. 9. Note that in the 2020 phone survey the unit of observation is the individual, while in the 2018–2019 field survey the unit of observation is the household: in practice, this means that our simulations with the 2018–2019 field survey dataset reflect a programme that would provide benefits only to heads of household, and we do not account for household size in considering exclusion errors or social welfare. Future work could model phone-based targeting on a household basis by collecting phone numbers for all household members and calculating aggregate benefits assigned to each household; given survey data limitations we cannot perform this analysis.

With geographic targeting, the primary counterfactual approach considered by the government of Togo in implementing its rural assistance programme, we assume that the programme would target geographic units in order from poorest to wealthiest, and that all individuals in targeted units would be eligible for benefits. We report results from two different approaches to geographic targeting: (1) a programme that targets the poorest prefectures (admin-2 region), defined as those prefectures with the lowest average predicted consumption based on a 2017 INSEED survey PMT; and (2) a programme that targets the poorest cantons (admin-3 region), defined as those cantons with the lowest average wealth based on high-resolution micro-estimates of wealth inferred from satellite imagery. When targeting the *n* poorest geographic regions would result in more than 29% of individual receiving benefits, then *n* − 1 regions are targeted fully, and individuals from the *n*th poorest region are selected randomly until the 29% threshold is reached. See Supplementary Fig. 5 and ‘Poverty maps’ for the poverty maps used for geographic targeting. (While this purely geographic approach was considered carefully by the Government of Togo, it is less common in non-emergency settings, when other data can inform targeting decisions. For instance, it is common to combine some degree of geographic targeting with community-based targeting and/or proxy means tests.)

In occupation-based targeting, we first evaluate the effectiveness of targeting informal workers, which is the eligibility criteria used by Novissi when it was first launched in April 2020, and which served as the basis for paying roughly 500,000 urban residents. In practice, this process involves categorizing the occupation of every individual respondent in both surveys as either formal or informal (including unemployed), applying the same definition of informality that was used by the Novissi programme. In the simulations, informal workers are targeted first (in random order if there are more informal workers than can receive benefits) and formal workers are targeted last (also in random order, if the available benefits exceed the number of informal workers).

We also develop and test a hypothetical occupation-based approach, which we refer to as ‘optimal occupation-based targeting’, which assumes that the policymaker had high-quality consumption data on the consumption of workers in each occupation and used that information to target the poorest occupations first. Although this approach was not considered in Togo’s pandemic response, it was feasible with the data sources available in Togo at the time, and represents an upper-bound on the performance of a hypothetical occupation-based targeting system. We simulate this optimal occupation-based approach by calculating the average consumption of each occupation in the 2018–2019 field survey; occupations are then targeted in order of increasing average consumption. The average consumption of each occupation category is shown in Supplementary Table 5. Note that because agricultural workers are the poorest category and make up 29% of the observations in the 2018–2019 field survey dataset and 41% of the observations in the 2020 phone survey dataset, in practice the precision and recall metrics reported in our targeting simulations reflect systems of occupation-based targeting that would prioritize agricultural workers only.

Of primary interest in the targeting evaluation is the performance of the targeting approaches based on mobile phone data. The phone-based (machine-learning) approach is the one described in the main text, which uses machine learning to construct a poverty score from rich data on mobile phone use and prioritizes the individuals with the lowest poverty scores (‘Machine-learning methods’). For reference, we also calculate the performance of a more parsimonious ‘phone (expenditures)’ model, which prioritizes the individuals with the smallest total phone expenditures (‘Parsimonious phone expenditure method’).

For completeness, our simulations also include results from targeting methods that were not feasible for the Novissi programme, as the data required to implement those methods were not available when Novissi was launched (though Togo plans to create a foundational unique ID system and comprehensive social registry in 2022)^54^. In particular, we simulate targeting using an asset-based wealth index, constructed as described in ‘Survey data.’ For the hypothetical national simulations using the 2018–2019 field survey dataset, we also simulate targeting using a PPI and PMT. Finally, when simulating targeting the hypothetical national programme restricted to rural areas (Supplementary Table 1), we also simulate targeting on a rural-specific PMT (see Differences in rural and national evaluations’). We cannot simulate PPI or PMT-based targeting using the 2020 phone survey since the necessary data were not collected.

An important caveat is that the PMT that we use in the 2018–2019 survey is ‘perfectly calibrated’ in the sense that it is both trained and evaluated on the same sample. In real-world settings, the predictive accuracy of a PMT declines as the time increases between the time of calibration and the time of application^27,29^. As such, the performance of the PMT we report is likely an upper bound of the performance of a real-world PMT.

For the PMT in the 2018–2019 field survey dataset, as well as for CDR-based wealth estimates in both datasets, predictions are produced out-of-sample over cross validation so that they can be fairly evaluated in targeting simulations. Specifically, in each case, the training dataset is divided into ten cross validation folds; the machine-learning model is trained on nine of the ten folds and used to produce predictions for the final fold. The training-and-prediction regime is repeated for all ten folds.

#### Measures of targeting quality

For each targeting method, we calculate two ‘threshold-agnostic’ metrics of targeting accuracy—metrics that capture relationships between continuous measures of poverty rather than focusing on accuracy for targeting a specific portion of the population. These are:

##### Spearman correlation coefficient

Spearman’s rank correlation coefficient is the Pearson correlation between the rank values of the true and proxy measures of poverty. We focus on the Spearman correlation rather than standard Pearson correlation as a measure of targeting quality because targeting concerns itself only with the ordering of observations according to poverty. Spearman’s correlation coefficient is calculated as follows:

$$\rho =1-(\frac{6\mathop{\sum }\limits_{i=1}^{N}{({r}_{i}-{\hat{r}}_{i})}^{2}}{N({N}^{2}-1)}),$$

where *N* is the total number of observations, *r*_*i*_ is the rank of observation *i* according to the ground truth poverty measure, and ${\hat{r}}_{i}$ is the rank of observation *i* according to the proxy poverty measure.

##### ROC curves and area under the curve

**Extended Data Fig. 4:**
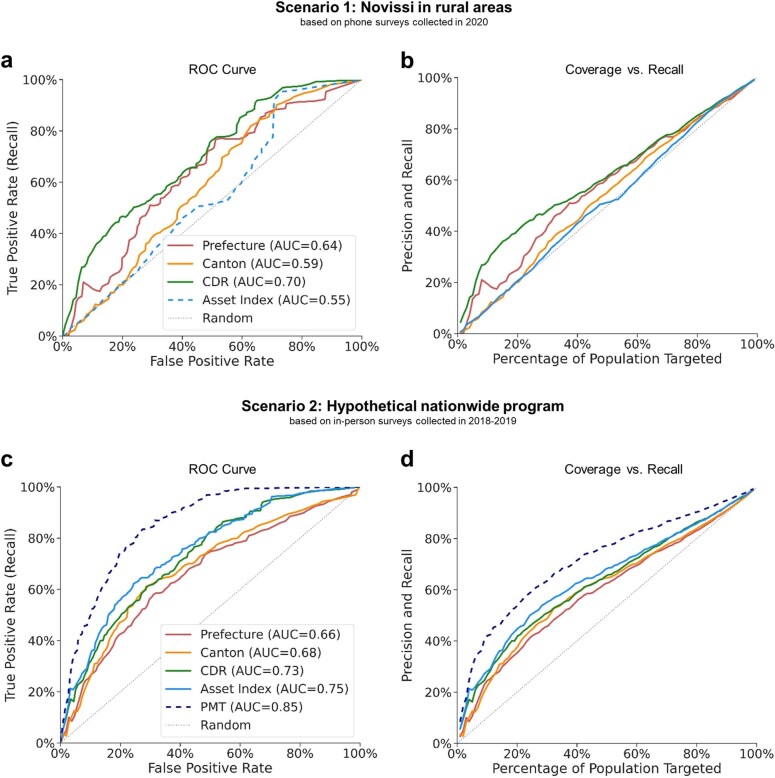
Targeting performance at different levels of program coverage. Top figures (**a** and **b**) show performance for the rural Novissi program, evaluated using 2020 phone survey. Bottom figures (**c** and **d**) correspond to the hypothetical national program, evaluated using the 2018–2019 field survey. ROC curves on left (**a** and **c**) indicate the true positive and false positive rates at different targeting thresholds. Coverage vs. Recall figures on right (**b** and **d**) show how precision and recall vary as the percentage of the population receiving benefits increases, i.e., they indicate the precision and recall for reaching the poorest *k%* of the population in programs that target the poorest *k%*. (Precision and recall are thus the same for each value of *k* by construction; see Methods, ‘Measures of targeting quality’).

Following ref. ^3^, we trace receiver operator characteristic (ROC) curves that describe the quality of a targeting method at counterfactual targeting thresholds (Extended Data Fig. 4, left figures). At each counterfactual targeting threshold *T* we simulate targeting *T*% of observations according to the proxy poverty measure in question and calculate the true positive rate (TPR) and false positive rate (FPR) of the classifier with respect to reaching the *T*% poorest according to the ground-truth poverty measure. By varying *T* from 0% to 100%, we construct the ROC curves shown in Extended Data Fig. 4. The area under the curve (AUC) is used to summarize the targeting quality, with a random targeting method achieving an AUC of 0.5 and perfect targeting an AUC of 1. For convenience, we also include ‘coverage vs recall’ figures (right figures of Extended Data Fig. 4) that show how programme recall varies as the eligible percentage of the population increases. Note that since recall is another name for the true positive rate, Extended Data Fig. 4b, d represent a rescaling of the ROC curves in Extended Data Fig. 4a, c.

##### Targeting accuracy

Our analysis focuses on analysing the performance of a quota-based approach that ranks individuals from predicted poorest to predicted wealthiest, then targets the poorest 29% of individuals. We use the quota of 29% since the rural Novissi programme had sufficient funding to provide benefits to the poorest 29% of registrants in eligible cantons. (This quota-based approach is not the only way that poverty scores could be used in targeting, though it is the only approach that we evaluate: for instance, a threshold-based approach might target everyone below a threshold poverty score; alternative approaches might provide cash transfers of different sizes depending on the poverty score of the beneficiary^4^.) The 29th percentile corresponds to a consumption threshold of US$1.17 per day in the 2018–2019 field survey dataset, and a PMT threshold of US$1.18 per day in the 2020 phone survey dataset. We calculate the following metrics to describe how accurately targeting the poorest 29% according to each targeting method reaches (1) the 29% truly poorest, (2) those below the international poverty line of US$1.90 per day (57% of observations in the 2018–2019 field survey, and 76% of observations in the 2020 phone survey), and (3) those below the extreme poverty line, which was defined as three-quarters of the poverty line, or US$1.43 per day (41% of observations in the 2018–2019 field survey, and 53% of observations in the 2020 phone survey):

- Accuracy: Classification accuracy measures the proportion of observations that are identified correctly (targeted observations that are poor according to the ground-truth poverty measure, and non-targeted observations that are not poor according to the ground-truth wealth measure). ${\rm{Accuracy}}=\,\frac{{\rm{TP}}+{\rm{TN}}}{{\rm{TP}}+{\rm{FP}}+{\rm{TN}}+{\rm{FN}}}$.
- Recall: Recall measures the proportion of all poor observations that are reached by a given targeting method. ${\rm{Recall}}=\frac{{\rm{TP}}}{{\rm{TP}}+{\rm{FN}}}$. Recall is closely related to the concept of exclusion errors (that is, the fraction of true poor who do not receive benefits, $\frac{{\rm{FN}}}{{\rm{TP}}+{\rm{FN}}}$), since ${\rm{Recall}}=1-{\rm{Exclusion\; error}}$.
- Precision: Precision measures the proportion of targeted observations that are poor according to the ground-truth poverty measure. ${\rm{Precision}}=\frac{{\rm{TP}}}{{\rm{TP}}+{\rm{FP}}}$. Precision is closely related to the concept of inclusion errors (that is, the fraction beneficiaries who are non-poor, $\frac{{\rm{FP}}}{{\rm{TP}}+{\rm{FN}}}$), since ${\rm{Precision}}=1-{\rm{Inclusion\; error}}$.
- Exclusion error: The proportion of true poor excluded from benefits. Defined as $\frac{{\rm{FN}}}{{\rm{TP}}+{\rm{FN}}}$.
- Inclusion error: The proportion of beneficiaries who are not poor, that is, $\frac{{\rm{FP}}}{{\rm{TP}}+{\rm{FP}}}$.

Note that the poverty lines are applied to consumption expenditure in the 2018–2019 field survey dataset, and to the PMT estimates in the 2020 phone survey dataset.

#### Differences in rural and national evaluations

The results in Table 1 indicate that the phone-based targeting approach—as well as the counterfactual targeting approaches—was more effective in the actual rural Novissi programme (columns 3 to 6 of Table 1) than it would have been in a hypothetical nationwide programme (columns 7 to 10 of Table 1). There are several factors that may account for these differences. Some of these factors are difficult for us to test empirically, for instance the fact that the surveys were conducted at different points in time, used different teams of enumerators, and different data collection modalities (phone versus in person). We investigate two factors that we can explore empirically: the geographic concentration of each survey and the ground truth measure of poverty (consumption versus PMT). We additionally explore whether targeting results are sensitive to the use of a nationwide PMT versus a rural-specific PMT.

##### Geographic concentration

Whereas the rural Novissi evaluation focuses on Togo’s 100 poorest cantons, the hypothetical national programme is evaluated nationwide (397 cantons). We therefore present results in Supplementary Table 1 that restrict the simulation of the hypothetical national programme to the 2,306 households in rural areas (out of 4,171 total). Comparing the results in Supplementary Table 1 to the last four columns of Table 1, we find that the performance of all methods drops, as would be expected when the beneficiary population is more homogeneous. The relative difficulty of estimating poverty among rural populations is also evident in Extended Data Table 6: the CDR-based method’s performance at predicting both consumption and the PMT is lower when the analysis of the 2018–2019 survey is restricted to the rural population (panel A vs panel B). Importantly, we also observe that the relative performance of phone-based targeting increases: whereas the CDR-based method performed worse than the asset index and only slightly better than canton-based targeting in the full nationwide evaluation (last four columns of Table 1), the CDR-based method is on par with the asset index and substantially better than canton-based targeting when the nationwide survey is limited to rural areas (Supplementary Table 1).

##### Consumption versus PMT

Whereas the national evaluation uses a measure of consumption as ground truth, the rural Novissi evaluation uses a PMT as ground truth. Supplementary Table 7 therefore simulates the hypothetical national programme using a PMT as ground truth. Comparing the results in Supplementary Table 7 to the last four columns in Table 1, we find that using a PMT rather than consumption as ground truth increases targeting accuracy across all of the targeting methods. However, switching from consumption to the PMT does not substantially improve the performance of the phone-based method relative to the counterfactual approaches. This latter finding suggests that the use of the PMT is likely not a major source of the difference between the relative performance of the CDR-based method in the rural Novissi programme (columns 3 to 6 of Table 1) and the hypothetical nationwide programme (columns 7 to 10 of Table 1).

##### National PMT versus rural PMT

As the best predictors of welfare differ for rural and urban populations, we explore whether targeting results change when the PMT is calibrated using a rural rather than national population. Specifically, we construct a rural-specific PMT using the same methodology described in ‘Survey data’, but restricting the training data to observations in the 2018–2019 field survey that are in rural areas. This rural PMT explains 17% of the variation in log-transformed consumption in rural areas, and is highly correlated (Pearson correlation = 0.75) with the general PMT. We then produce rural PMT estimates for respondents to the 2020 phone survey, and retrain the phone-based poverty prediction model to predict the rural-specific PMT in that population. Supplementary Table 8 then presents results from simulating with the rural PMT as ground truth. Comparing Supplementary Table 8 to columns 3 to 6 of Table 1, we observe a noticeable improvement in the performance of the asset index, but other results are largely unchanged.

Relatedly, Extended Data Table 3 shows the feature importances for different phone-based prediction models. Panels A and B show the top-10 features for the main models presented in Table 1, that is, for predicting a PMT in the 2020 rural phone survey, and predicting consumption in the 2018–19 nationwide household survey. Panels C and D show the top-10 features for predicting a PMT in the 2018–19 survey, and predicting a PMT in the 2018–2019 household survey, restricted to rural areas. The feature importances for the two national-scale models are similar, suggesting the role of the ground truth poverty measure may not be as important as the role of geography in creating the poverty prediction models. The feature importances for the two rural-focused models are less similar, which may be due to the fact that the 2020 phone survey is concentrated in the 100 poorest cantons, while in panel D we restrict to rural areas, but these rural areas still cover the entire country.

Taken together, the results in this subsection suggest that the benefits of phone-based targeting are likely to be greatest when the population under consideration is more homogeneous, and when there is less variation in other factors (such as place of residence) that are used in more traditional approaches to targeting.

#### Location-based targeting

Several results emphasize the importance of geographic information in effective targeting. In particular, we observe that basic geographic targeting performs nearly as well as phone-based targeting in specific simulations—in particular, in simulations of a nationwide programme that can afford to target a large proportion of the total population (for example, Extended Data Table 2). We also found that location-related features from the CDR are important in the phone-based prediction model (‘Machine-learning methods’).

For these reasons, Supplementary Table 9 explores the extent to which targeting could be based on a CDR–location model that only uses the CDR to infer an individual’s home location (see Supplementary Methods section 4). As with the phone (expenditures) model, the CDR-location model may be attractive to implementers since the data and technical requirements are reduced^55^. In Supplementary Table 9, we observe that geographic targeting using phone-inferred home location is of slightly lower quality than geographic targeting using survey-recorded home location, and substantially worse than targeting using the machine-learning approach.

We also investigate the correlation between different sources of information on an individual’s location. Supplementary Table 10 compares three different methods for identifying an individual’s location, using roughly 4,500 respondents to the 2020 phone survey. At the prefecture (admin-2) level, most people (90%) self-declare living in the same canton in which they are registered to vote; there is also strong overlap between the individual’s CDR-inferred location and self-declared location (70%). The accuracy is substantially lower at the canton level, which is likely due to error in the CDR-inference algorithm when spatial units are small, as well as to confusion among respondents as to which canton they live in (for example, most respondents were confident in naming their village, but did not always know their canton).

Supplementary Table 11 presents additional analysis to compare the mobile phone activity of each subscriber with their home location, as recorded in the survey and as inferred from their CDR. We find that 62–85% of the average subscriber’s activity occurs in their home prefecture, and that all of the modal subscriber’s activity occurs in their home prefecture. These results are consistent with the importance of location-related features in the prediction algorithm (and the relatively low mobility of the rural Togolese population).

This analysis may also provide some context for the difference in the accuracy of the geographic targeting methods between the rural evaluation and the national evaluation in Table 1. While canton-based targeting performs better in the national evaluation, which is consistent with past work showing that finer-resolution geographic targeting is preferred to lower-resolution geographic targeting^21,56^, prefecture-based targeting counter-intuitively performs better in the rural evaluation. We suspect this discrepancy is caused by three main factors. First, we expect that the estimates of average canton wealth are likely to be noisier than the estimates of average prefecture wealth, because the prefecture estimates aggregate over a larger population and the canton estimates rely on satellite-based inferences. Second, in the rural evaluation the prefecture is an important component of the PMT that is used as the ground truth measure of poverty (see Supplementary Table 3), so prefecture targeting relies on information that is structurally incorporated into the ground truth outcome (unlike in the national evaluation, where the ground truth outcome is consumption). The results in Supplementary Table 7 are consistent with this second hypothesis: the gap between prefecture and canton targeting in the national evaluation in Table 1 is smaller when switching the ground-truth poverty outcome from consumption to the PMT. Third, locations in the rural phone survey were self-reported, whereas locations were recorded on GPS devices by enumerators in the national survey; as noted, many respondents expressed confusion about their home canton. (The results in Supplementary Table 9, however, are not consistent with this third hypothesis: they indicate that targeting on canton inferred from mobile phone data is weaker than targeting on prefecture inferred from mobile phone data, suggesting that a difference in response quality between prefecture and canton in the survey is not a major factor in the difference in outcomes in the targeting simulations.)

#### Temporal stability of results

When simulating the performance of phone-based targeting, our main analysis uses each survey dataset to both train the machine-learning model and, via cross-validation, to evaluate its performance. These measures of targeting performance thus indicate what should be expected when training data (that is, the ground truth measures of poverty and the matched CDR) are collected immediately prior to a programme’s deployment. This best-case scenario is what occurred in Togo in 2020: the phone survey was completed in October 2020 and Novissi was expanded beginning in November 2020. In other settings, however, it may not be possible to conduct a survey before launching a new programme; it may likewise not be possible to access up-to-date mobile phone data.

To provide an indication of how long phone-based models and predictions remain accurate, Extended Data Table 4 compares (1) the best-case scenario to alternative regimes where (2) the training data are old but the CDR are current, and (3) the training data are old and the CDR are also old. In these simulations, the ‘old’ data are from the 2018–2019 national household survey and corresponding 2019 phone dataset; the ‘current’ data are the subset of 2020 phone survey respondents for whom CDR are available in 2019 and 2020 (*N* = 7,064). In all simulations, the 2020 PMT is used as the ground truth measure of poverty. Predictions for (1) are generated over tenfold cross validation; predictions for (2) and (3) are out-of-sample with respect to the training data, since the models are trained on the 2018–2019 field survey. (An additional issue with (3) is turnover on the mobile phone network: 1,851 (21%) of phone numbers collected in the 2020 survey were not on the mobile phone network in 2019, and therefore cannot be associated with a wealth prediction in (3). See also Supplementary Fig. 6 for detailed information on rates of turnover on the mobile phone network.)

The results in Extended Data Table 4 indicate that predictive performance decreases when the model is out of date, and decreases even further when the CDR are out of date. This is to be expected, since roughly two years elapsed between the old and current periods: in addition to changes in how people use their phones (which would disrupt the accuracy of the predictive model), the actual economic status of some individuals may have changed—for instance, owing to the COVID-19 pandemic. There are also other important differences between the 2018–19 national household survey and the 2020 phone survey that could affect the extent to which a model trained on the former could accurately predict outcomes in the latter (such as the mode of data collection, the geographic concentration of the sample, and so forth; see ‘Differences in rural and national evaluations’).

For the main simulations focused on reaching the poorest 29%, Extended Data Table 4 suggests that accuracy decreases by 3–4 percentage points (4–6%) and precision decreases by 5–7 percentage points (10–14%) when out of date models and CDR are used for targeting. These losses are nearly as large as the gains of phone-based targeting over geographic targeting observed in Table 1, which emphasizes the importance of having current and representative training data for real-world deployment of phone-based targeting. However, in absolute levels, the phone-based predictions remain reasonably accurate despite the two-year gap between the training and test environments (that is, the Spearman correlation (*ρ*) with ground truth is 0.35–0.36.

#### Social welfare

Using the two matched survey-CDR datasets, we calculate aggregate utility under each of the targeting methods using a social welfare function. Following ref. ^3^ we rely on CRRA utility, which models individual utility as a function of pre-transfer consumption and transfer size:

$$U=\frac{\mathop{\sum }\limits_{i=0}^{N}{({y}_{i}+{b}_{i})}^{1-\rho }}{1-\rho }$$

Where *N* is the population size, *y*_*i*_ is the consumption of individual *i*, and *b*_*i*_ are the benefits assigned to the individual. Following ref. ^3^, we use a coefficient of relative risk-aversion $\rho =3$. To reflect the policy design of the Novissi programme, we assume that all beneficiaries who receive a benefit receive the same value *b*_*i*_ *= b*. (In principle, the benefit *b*_*i*_ paid to *i* could depend on characteristics of *i*, such as *i*’s level of poverty. Although such an approach would substantially increase total welfare, in practice it is much more difficult to implement). To construct the social welfare curves, we:

- Calculate a total budget available for each of the two datasets. We focus on programmes that have a budget size analogous to that of rural Novissi, which aimed to distributed approximately US$4 million among the 154,238 programme registrants, or US$25.93 per registrant. We therefore assign each dataset a total budget of US$25.93*N*, where *N* is the total size of the dataset.
- Simulate targeting *T*% of observations on the basis of each of our counterfactual targeting approaches.
- Assign equal benefits to each of the targeted observations, with the budget divided evenly among targeted observations (so lower targeting thresholds *T* correspond to more benefits for targeted individuals).
- Calculate aggregate utility by summing over benefits and consumption for each individual with the CRRA utility function. Note that non-targeted individuals are included in the welfare calculation; they are merely assigned 0 benefits. For the 2018–2019 field survey dataset we use consumption expenditure for *y*_*i*_; for the 2020 phone survey dataset we use the PMT estimates.
- By varying *T* between 0% and 100% of observations targeted, we trace out the social welfare curves shown in Fig. 2.

#### Fairness

We are interested in auditing our targeting methods for fairness across sensitive subgroups. Note that that notions of parity and fairness are debated in machine learning and policy communities: ref. ^57^ describes how the three most popular parity criteria—demographic parity (benefits assigned to subgroups proportionally to their size), threshold parity (use of the same classification threshold for all subgroups), and error rate parity (equal classification error across subgroups)—are in tension with one another. Moreover, ref. ^33^ describe how tensions over parity criteria, prioritized subgroups, and positive discrimination lead to complicated prioritization compromises in the administration of targeted social protection programmes.

Here we focus on two targeting-specific parity criteria:

Demographic parity. A targeting method satisfying demographic parity will assign benefits to a subgroup proportionally to the subgroup’s presence in the population of interest. We evaluate demographic parity among the poor: that is, we compare the proportion of each subgroup living in poverty (below the 29th percentile in terms of consumption) to the proportion of each subgroup that is targeted (below the 29th percentile in terms of the proxy poverty measure used for targeting).

$${\rm{DP}}=\frac{{\rm{TruePositives}}+{\rm{FalsePositives}}}{{\rm{N}}}-\frac{{\rm{TruePositives}}+{\rm{FalseNegatives}}}{{\rm{N}}}$$

Normalized rank residual. We are interested in whether certain subgroups are consistently ranked higher or consistently ranked lower than they ‘should’ be by the counterfactual targeting approaches. We therefore compare the distributions of rank residuals across subgroups and targeting methods:

$${{\rm{RR}}}_{i}=\frac{{\hat{r}}_{i}-{r}_{i}}{N}$$

where ${\hat{r}}_{i}$ is the poverty rank of individual *i* according to the proxy poverty measure and *r*_*i*_ is the poverty rank of individual *i* according to the ground-truth poverty measure.

We focus on seven dimensions for parity: gender, ethnicity, religion, age group, disability status, number of children, and marital status. We also evaluate parity across whether an individual is ‘vulnerable’, where vulnerability is defined as one of the following traits: {female, over age 60, has a disability, has more than five children, is single}. We conduct this analysis using demographic information about the head of the household in the 2018–2019 field survey dataset, as these demographic variables were not all collected in the 2020 phone survey.

### Programme exclusions

In Table 2, we present information on sources of exclusion from the Novissi programme that are not inherently related to targeting. These estimates are drawn from diverse sources of administrative and survey data, specifically:

Voter ID penetration. According to government administrative datasets, 3,633,898 individuals were registered to vote in Togo by late 2019. The electoral commission of Togo reports that this corresponds to 86.6% of eligible adults. Although the total adult population in Togo is hard to pin down (the last census was in 2011), Togo’s national statistical agency (https://inseed.tg/) estimates that there are 3,715,318 adults in Togo, whereas the United Nations estimates 4.4 million adults in Togo^45^, implying a voter ID penetration rates of 82.6% or 97.8%.

Phone penetration. In the 2018–2019 field survey, 65% of individuals reported owning a mobile phone (Supplementary Fig. 3a) and 85% of households included at least one individual who owns a phone (Supplementary Fig. 3b). In rural areas, these rates drop to 50% of individuals and 77% of households. Rates of phone ownership are substantially lower among women (53%) than among men (79%), especially in rural areas (33% for women and 71% for men). These household survey-based estimates likely represent a lower bound, given the steady increase in phone penetration between 2018 and 2020. The Togolese government estimates 82% SIM card penetration in the country (though some people may have multiple SIM cards)^58^. On the basis of data from the mobile phone companies, we observe 5.83 million unique active SIMs in Togo between March and September 2020.

Past phone use. In order to construct a phone-based poverty estimate for a subscriber, they had to place at least one outgoing call or text on the mobile phone network in the period of mobile network observation prior to the programme’s launch (March – September 2020, with programme registrations in November-December 2020). In Togo, a lower bound on this source of exclusion is the typical monthly rate of mobile phone turnover, which we estimate to be roughly 2.5% (see Supplementary Fig. 6). An upper bound is closer to 27%, which is the number of SIM cards that registered for Novissi November-December 2020 who did not make an outgoing transaction in the March-September. This discrepancy may be due to (1) individuals buying new SIM cards specifically to register for Novissi; or (2) individuals registering for Novissi using existing SIM cards that were not in active use, for instance the SIM cards in multi-SIM phones. Based on qualitative observation, multi-SIM phones are very common in Togo, and secondary or tertiary SIMs are infrequently used (or not used at all). It is possible that families registered one household member on a primary SIM and others on secondary or tertiary SIMs that may have had no previous network activity.

Programme awareness. Since individuals had to register for the Novissi programme to receive benefits, programme advertising and population awareness was a key goal. The programme was advertised via radio, SMS, field teams, and direct communication with community leaders at the prefecture and canton level. In total, 245,454 subscribers attempted to register for the programme. Although we do not observe the prefecture and canton of subscribers who attempt but do not succeed in registering in our administrative data, we know that 87% of successful registrants are in cantons eligible for benefits. Assuming the rate is approximately the same for attempters, we expect that around 213,545 of the attempters are in eligible cantons. The total voting population in eligible cantons is 528,562, for an estimated attempted registration rate of 40.40%.

Registration challenges. Registration for the Novissi programme required the completion of a short (5 question) USSD survey. Of the 245,454 subscribers that attempted to register for the programme, 176,517 succeed, for a 71.91% rate of registration success.

#### Overlaps among sources of exclusion

The above sources of exclusion are not independent and are therefore not cumulative. For instance, individuals who are not registered to vote may also be systematically less likely to have a mobile phone. For this reason, Extended Data Table 5 uses the 2020 phone survey dataset—restricted to respondents who report living in an eligible canton—to calculate overlaps in sources of exclusion to the poor, including voter ID possession, programme awareness, registration challenges, and targeting errors using the phone-based targeting method. We cannot account for mobile phone ownership in this analysis since the 2020 survey was conducted over the phone, and sampled based on past CDR (see Supplementary Methods, section 5).

The final three columns of Extended Data Table 5 show, based on the 2020 phone survey dataset, average characteristics of the population ‘succeeding’ at each step: average PMT, per cent women and average age. The first panel shows successive exclusions for the entire population; the second panel focuses on just the poorest 29% (that is, those who ‘should’ be receiving aid, were everyone to register for the programme and were the targeting algorithm perfect). In panel A, we observe that to a certain extent the ‘right’ types of people are dropping out at each step, which would be consistent with self-targeting observed in other contexts^26^: in particular, those who attempt to register are poorer than the overall population (average PMT = 1.45 vs 1.62). There are little differences in the share of the successful population who are women or average age, except in the targeting stage.

Comparing panels A and B of Extended Data Table 5, we observe that the recall of the targeting algorithm is substantially higher among the population that owns a voter ID and succeeds in registration for the programme (61%, as shown in Extended Data Table 5, last row) than the overall population surveyed in the 2020 phone survey (47%, as shown in Table 1, row 4). This may be due to self-selection (that is, the type of poor people who register for Novissi tend to also have low phone-based poverty scores). However, it could alternatively suggest that the phone-based targeting algorithm is best at identifying the poor among the types of subscribers who are aware of and register to the Novissi programme.

### Reporting summary

Further information on research design is available in the Nature Research Reporting Summary linked to this paper.

## Online content

Any methods, additional references, Nature Research reporting summaries, source data, extended data, supplementary information, acknowledgements, peer review information; details of author contributions and competing interests; and statements of data and code availability are available at 10.1038/s41586-022-04484-9.

### Supplementary information

Supplementary Information This file contains Supplementary Discussion, Methods, Supplementary Figs. 1–11, Tables 1–13 and references.

Supplementary Information Reporting Summary.

## Figures and Tables

**Extended Data Table 1:**
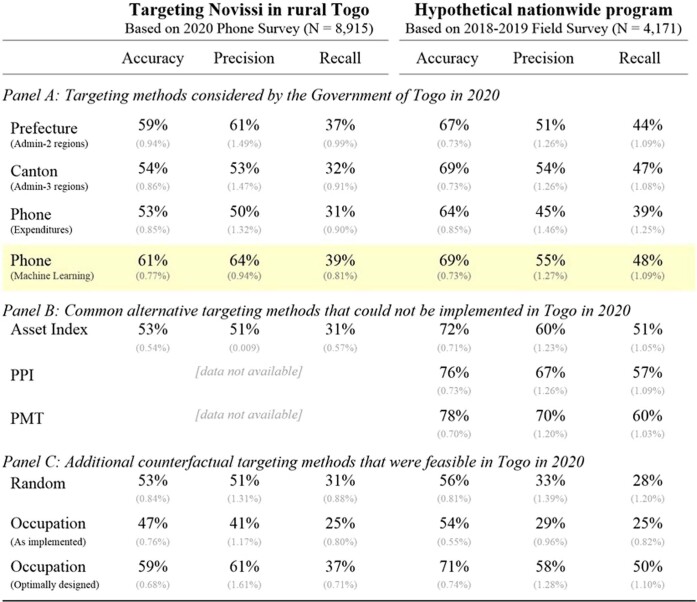
Performance of targeting households below the extreme poverty line

Analysis is similar to that presented in Table 1, but targeting is evaluated on the extent to which each method (still targeting the poorest 29%) provides benefits to individuals consuming less than the international extreme poverty line, set at 75% of the international poverty line or USD $1.43 per person per day (53% of observations in the 2020 phone survey dataset and 41% of observations in the 2018-2019 field survey). Spearman correlation and AUC are not reported here as they do not depend on the classification threshold, and are thus identical to the values reported in Table 1.

**Extended Data Table 2:**
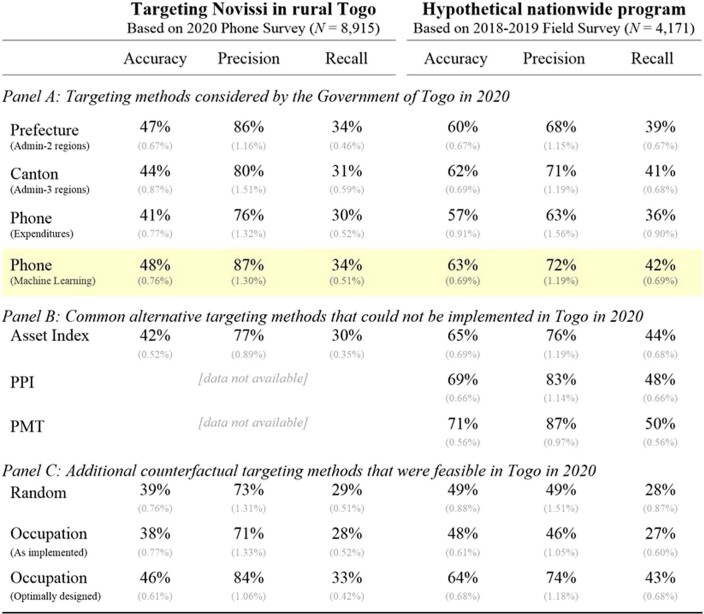
Performance of targeting households below the poverty line

Analysis is similar to that presented in Table 1, but targeting is evaluated on the extent to which each method (still targeting the poorest 29%) provides benefits to individuals consuming less than the international poverty line of USD $1.90 per person per day (76% of observations in the 2020 phone survey dataset and 57% of observations in the 2018–2019 field survey). Spearman correlation and AUC are not reported here as they do not depend on the classification threshold, and are thus identical to the values reported in Table 1.

**Extended Data Table 3:**
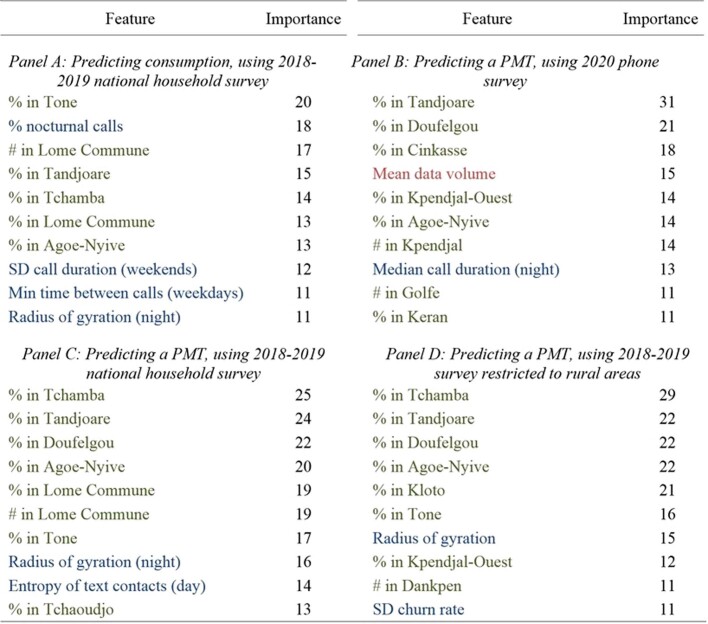
Feature importances

Feature importances for the 10 most important features selected by machine-learning models trained to predict (a) Proxy Means Test from CDR, using a 2020 phone survey of mobile subscribers in Togo’s 100 poorest cantons (*N* = 8,915); (b) consumption from CDR in the 2018-2019 field survey dataset (*N *= 4,171); (c) PMT from CDR in the 2018-2019 field survey dataset (N = 4,171), and (d) PMT from CDR in the 2018-2019 field survey dataset restricted to rural areas (N = 2,306). Feature importance is calculated based on the total number of times a feature is split upon in the prediction ensemble. Features are color-coded as follows: CDR features are shown in blue, location features in green, mobile money features in purple, and mobile data features in red.

**Extended Data Table 4:**
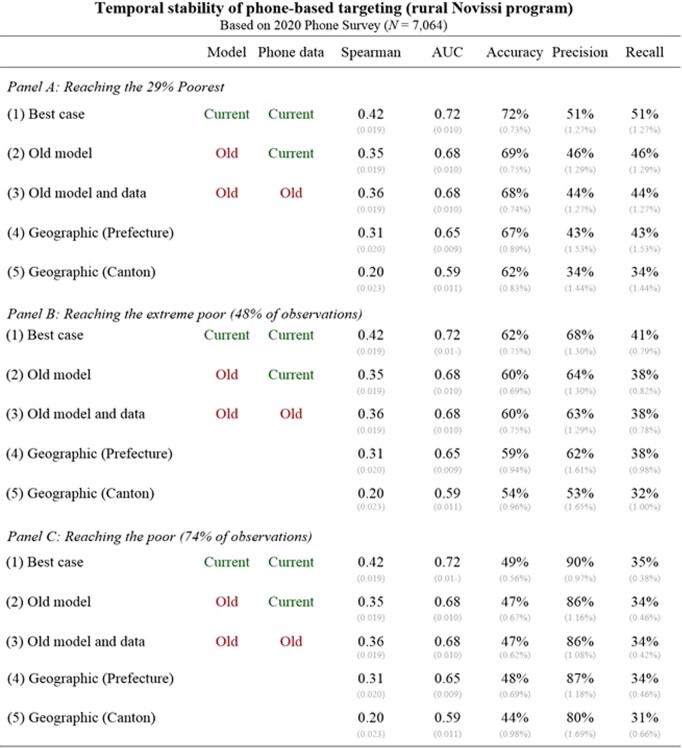
How quickly does the accuracy of a phone-based targeting model degrade?

Table compares three scenarios: (1) “Best case”: when the model is calibrated using survey data and phone data gathered just before deployment; these results are comparable to the paper’s main analysis (slight differences are due to the sample restrictions described below); (2) “Old model”: when the model is trained using a survey conducted two years before deployment, but the phone data are collected just before deployment; and (3) “Old model and data”: when the phone-based wealth estimates are generated using survey and phone data from two years prior. Rows (4) and (5) show geographic targeting results using the same sample as in rows (1) – (3). In the simulations, the “old” data are from the 2018-19 national household survey and corresponding 2019 phone dataset; the 2020 phone survey PMT is used as the ground truth measure of poverty (restricted to respondents for whom CDR are available in 2019 and 2020, *N* = 7,064).

**Extended Data Table 5:**
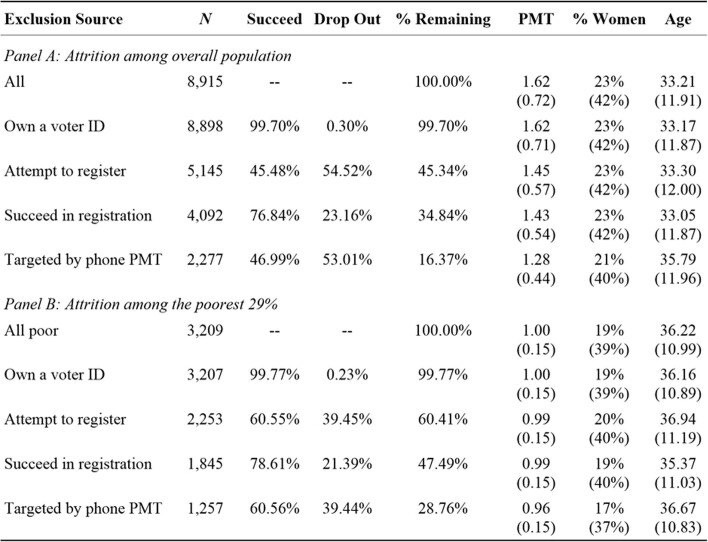
Overlapping sources of exclusion from rural Novissi

Progressive sources of attrition from the rural Novissi program, where each row shows exclusion conditional on exclusions from preceding rows. The final three columns show characteristics of the population “succeeding” at each step. Panel A: Results estimated using the 2020 phone survey (*N* = 8,915). Panel B: Results estimated for just the poorest 29% from the 2020 survey (*N* = 3,209). There is no attrition based on mobile phone ownership or past phone use in this sample (in contrast to Table 2) since only active phone users were sampled for the phone survey. Values reweighted using sample weights. (In some cases, sample weights create large differences in the weighted and raw percentages. For instance, 5,145 out of 8,898 voters (57.8%) attempt to register (Panel A), but the weighted percentage is 45.5%. The importance of sample weights is consistent with the wide distribution of sample weights shown in Supplementary Fig. 10).

**Extended Data Table 6:**
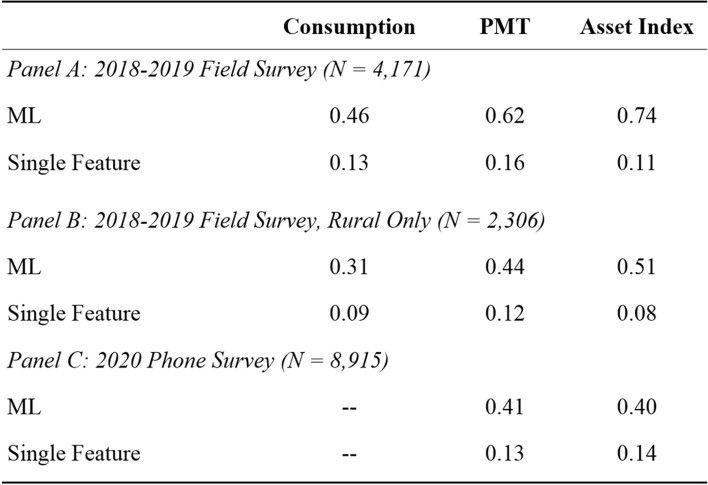
Performance of phone-based approach to predicting wealth and consumption

Accuracy (Pearson correlation coefficients) for predicting poverty measures from CDR. ML predictions are produced over 5-fold cross validation and evaluated for pooled correlation. The “single feature” model estimates wealth and consumption based on the individual’s total expenditures on calling and texting.

## Data Availability

The data used in this analysis include data that are available from public online repositories, data that are available upon request of the data provider, and data that are not publicly available because of restrictions by the data provider. The micro-estimates of wealth and population density used to derive satellite-based poverty maps are available from the Humanitarian Data Exchange (https://data.humdata.org/dataset/relative-wealth-index and https://data.humdata.org/dataset/highresolutionpopulationdensitymaps-tgo). The survey datasets are available upon request from the Institut National de la Statistique et des Études Economiques et Démographiques (https://inseed.tg/ and inseed@inseed.tg). The mobile phone data and administrative data from the Novissi programme contain proprietary and sensitive information, and cannot be publicly released. Upon reasonable request, we can provide information to academic researchers on how to contact mobile network operators and the Togolese government to request these datasets.

## Code availability

The code used for these analyses is publicly available at the GitHub repository located at https://github.com/emilylaiken/togo-targeting-replication/.
