# Supplementary material for: Machine learning and phone data can improve targeting of humanitarian aid
Source: Nature. 2022 Mar 16;603(7903):864–70. doi: 10.1038/s41586-022-04484-9 (PMC8967719; doi:10.1038/s41586-022-04484-9)
Supplement: Supplementary file 1 — This file contains Supplementary Discussion, Methods, Supplementary Figs. 1–11, Tables 1–13 and references. [file 41586_2022_4484_MOESM1_ESM.pdf]

nature portfolio

https://doi.org/10.1038/s41586-022-04484-9

Supplementary information

Machine learning and phone data can improve targeting of humanitarian aid

In the format provided by the authors and unedited

Supplementary Materials for

Machine Learning and Phone Data Can Improve Targeting of Humanitarian Aid

Emily Aiken, Suzanne Bellue, Dean Karlan, Chris Udry, Joshua Blumenstock

Correspondence to: jblumenstock@berkeley.edu

Contents

Supplementary Discussion 2
1. Related work 2
2. Limitations and Concerns 2
Supplementary Methods 6
3. Selection of Variables for Proxy-Means Test 6
4. Home Location Inference from Mobile Phone Data 7
5. Design of the 2020 Phone Survey 9
References 13
Supplementary Figures 16
Supplementary Tables 27

Supplementary Discussion

1. Related work

There is a rich history of theoretical and empirical work that compares and evaluates methods for targeting social transfer programs. While there is increasing interest in "universal basic income", in which everyone is eligible for transfers, most countries use one or more targeting mechanisms to determine eligibility[1]. Typically, the goal of targeting is to ensure that the poorest individuals receive transfers.[i]

Many programs include some degree of *self-targeting*, in which beneficiaries are required to take some action in order to receive benefits[3–5]. If the benefits of the program, relative to the costs associated with that action, are higher for poorer people, self-targeting can direct a greater share of benefits to the poor. *Geographic targeting* is also common, whereby benefits are restricted to individuals who live in specific regions[6,7]. Empirical evidence on geographic targeting indicates that more granularly targeted programs can be more effective at prioritizing the poor, but the implementation of such programs requires fine-grained poverty maps and distribution mechanisms that can be deployed in small regions[8–10]. With *proxy means tests (PMT)*, a number of variables are collected for each household, which are then used to impute an approximate measure of consumption or wealth for that household.[11,12] Likewise, a simple poverty scorecard or *poverty probability index (PPI)* uses a small number of variables to impute a poverty score.[13,14] PMTs and PPIs are frequently used in LMICs, but do require that the government collect and maintain a comprehensive social registry that records the information of each household. Finally, *community-based targeting (CBT)* approaches rely on members of the community to identify the poorest households in the area[15,16]. CBT-based approaches do not always target the lowest-consumption households, but allow the community to define their own notion of poverty, which can lead to higher satisfaction among community members[4] but may also lower perceptions of program legitimacy[17].

2. Limitations and Concerns

While mobile phone data can create new options for the accurate targeting of humanitarian aid, there are several important limitations. A full discussion of the social, political, and ethical implications of these issues has been the focus of prior work and is beyond the scope of this article[18–22]; we nonetheless highlight a few key issues that we believe require careful consideration before these methods can be implemented in a policy environment:

Phone ownership and access: As discussed in Methods, 'Program Exclusions', many individuals in LMICs do not own mobile phones. Thus, any targeting system based on mobile phone data may exclude those without phones from receiving program benefits. In the case of the Novissi program, the government used the mobile money system to disburse the cash transfers as a way to minimize human contact during the pandemic. Thus, in Togo, the use of phone data for

[i] How a program defines "poverty" is also a source of considerable debate[2]. In this paper, we use the term "socioeconomic status" somewhat loosely to refer to an individual's access to resources. By contrast, we use "consumption" to refer to how much an individual spends or consumes, and "wealth" to refer to an individual's assets. "Poverty" is a condition in which an individual's access to resources falls below a minimal level, based on consumption or wealth, as described in Methods, 'Survey Data'.

targeting only created additional exclusions by requiring that program registrants had made at least one transaction on their SIM card in the months prior to registration. In general, incomplete mobile phone access highlights the need to allow for alternative pathways for individuals to register and receive benefits, and to create additional mechanisms for appeals, grievance redress mechanisms, and manual enrollment.

Data privacy: Mobile phone metadata, even when pseudonymized, contains sensitive information. Methods, 'Data Privacy Concerns' describes several steps taken to protect the confidentiality of the data used in this project. More generally, special considerations arise when using personal data from vulnerable populations[23], and human rights doctrine emphasizes that any form of communications surveillance should be "necessary and proportionate"[24].

In implementing the approach described in this paper, we developed an IRB protocol, as well as a data management plan, that was approved by U.C. Berkeley's Committee for the Protection of Human Subjects. We followed principles of data minimization to limit the data collected and stored, and implemented organizational safeguards to restrict access to data. As an example, only IRB-approved researchers ever received access to CDR; data from the phone companies were shared with neither the Government of Togo nor GiveDirectly. Even the poverty scores derived from the phone data were restricted to IRB-approved researchers; the only data the government received was the list of SIM cards belonging to eligible beneficiaries below the targeted poverty threshold.

Future projects using mobile phone data for targeting should ensure that principles of data minimization and data sunsetting restrict the use of sensitive data to social protection objectives and limit the potential for "function creep."[25] Further research on applying the guarantees of differential privacy to mobile phone metadata[26,27] or implementing federated learning systems[28] could reduce the risk of data misuse or central data breaches.

Data access and consent: The fact that our approach requires access to mobile phone data owned by private companies poses an obstacle to the immediate and widespread use of such data for targeting humanitarian aid. There now exist several general frameworks and recommendations to facilitate the use of CDR in humanitarian applications[19,29]. Yet such frameworks are still nascent, and without careful consideration may exclude important stakeholders and perspectives[22]; they also widen the scope for private companies to influence humanitarian and development decisions[30]. There also exist many ethical frameworks that rely on informed consent from participants for the use of personal data, including digital data such as CDR[31,32]. Future programs should consider how consent pathways can be integrated with phone-based targeting, including opt-in (calculating poverty scores only after consent is provided) and opt-out (scrubbing data if consent is not provided at the time of registration) options.

Data representativity: To train the machine learning models, ground truth measures of consumption and wealth were collected using in-person and phone surveys. Since response rates were imperfect in the phone survey, we reweighted survey observations to make the training data more representative of all mobile subscribers (Methods, 'Survey Data'). However, there are limits to the representativity of our training data, as dynamics of phone ownership and phone sharing vary across population subgroups (Supplementary Figure 3), and reweighting is an imperfect proxy.

To test for systematic bias based on data representativity, we perform ex-post audits to limit the likelihood that the trained models systematically disadvantage specific subgroups of the population (Methods, 'Fairness'), and find that the phone-based targeting method is no more biased than counterfactual targeting approaches. We believe such audits are essential to future work on wealth prediction and targeting based on nontraditional data. Audits could be improved with additional context-specific research about which sub-populations are at the greatest risk for systematic exclusion (for example, in this paper we test for bias across age groups, genders, ethnicities, and more), and on considering alternative definitions for bias and fairness.[33,34]

Unit of analysis: As noted in Methods, 'Experimental Design', our analysis focuses on *individuals* rather than *households* as the unit of analysis, partly reflecting the design of the Novissi program, and partly because there are no data in Togo that associate individuals with households. This limitation is important, since many real-world programs are targeted at the household level, but CDR are more naturally linked to individual subscribers. An important area for future work will thus be to explore the extent to which CDR can facilitate household-level targeting. Such work must account for the fact that a single SIM card is often shared across multiple members of the same household (and occasionally between households), and that some individuals use multiple SIM cards. Ideally, such an analysis would leverage authoritative data that uniquely identifies and links households, individuals, SIM cards, and phones.

Method of evaluation: Our main results are based on simulations of targeting methodologies using survey data collected prior to expansion of Novissi. An alternative approach to evaluating targeting performance would rely on survey data after program implementation, which would make it possible to more directly verify who did and didn't receive program benefits, address issues related to the unit of analysis described above, and better attribute exclusion errors to different aspects of program design. While public health considerations in Togo prevented us from conducting a post-program survey, we hope future implementations of phone-based targeting can use post-program surveys to provide complementary evidence to what is described in this paper.

Poverty dynamics: The phone-based approach we describe uses machine learning algorithms to predict which individuals are "poor", based on ground-truth assessment of poverty collected in surveys prior to program implementation. In the actual rural Novissi program, the ground truth measure of poverty was based on a proxy means test; in the hypothetical national program, ground truth is based on consumption (Methods, 'Survey Data'). However, particularly in the context of a crisis, an individual's poverty status can change; in such settings, pre-program poverty assessments may not accurately capture the population with the greatest need for support. Our data do not permit us to test whether phone data and machine learning can be used to determine if an individual has experienced a sudden fall in income or consumption, but we believe this is a promising area for future work.

Manipulation and gaming: When mobile phone data are used to determine eligibility for social benefits, individuals have incentives to strategically alter their behavior in order to "game" the system. This dilemma is not unique to phone-based targeting; it is a key consideration in the design of any targeting mechanism[35,36], and one that affects traditional proxy means tests and poverty scorecards[37,38]. However, recent evidence suggests that such distortionary effects may be limited[39], and complex eligibility criteria (such as the gradient boosting procedure described in Methods, 'Machine Learning Methods') should limit the scope for such gaming[40]. With Novissi

in Togo, the one-off nature of the program likely eliminated most scope for strategic behavior; however, if such an approach were used continuously over time, alternative "manipulation-proof" approaches to machine learning may be more appropriate[41].

General equilibrium considerations: Our analysis of targeting effectiveness assumes there are no general equilibrium effects of the program on prices, wages, or interactions with informal transfers or insurance. For example, geographic targeting of transfers might lead to localized inflows of cash transfers that are large relative to the local economy, leading to changes in local demand for goods or supply of labor and therefore prices, wages or profits of local businesses[42,43]. Similarly, since individuals are embedded in family and broader networks of informal transfers for redistribution, patronage and insurance and different targeting choices could have different effects on these existing informal arrangements[44,45]. Equilibrium effects such as these may have important implications for the eventual distribution of impacts from the transfers. However, to cause a reversal of the policy implication of our analysis, general equilibrium effects would need to be more nuanced than merely present – for example, it would need to be that the false negatives under one method are more likely to share resources than the false negatives on another method.

Supplementary Methods

3. Selection of Variables for Proxy-Means Test

Our proxy-means test is used in analysis for both the 2018-2019 field survey (where we evaluate the PMT's accuracy as a targeting mechanism) and the 2020 phone survey (where we use the PMT as a measure of ground-truth poverty in the absence of a consumption measure). We construct the PMT using all observations from the 2018-2019 field survey (N = 6,171). We begin by identifying all information on demographics and asset ownership collected in the field survey that may correlated with poverty. In total, we identify 56 variables, including information on household assets and housing quality, education, marital status, age, ethnicity, health, location, and more.

Our goal is to identify a small subset of variables that are most predictive of household consumption. We use stepwise forward selection to identify the most predictive feature subsets of size K, for K ranging from 1 to 30. Specifically, we randomly divide our survey observations into a training set (75%) and test set (25%). For K=1, we train a machine learning model to predict household consumption[ii] from each feature individually, and select the feature associated with the best model. For $K=2$, we test adding each remaining feature to our model, and select the feature that adds the most predictive power. We continue the process for all K up to 30.

We perform the stepwise forward selection process first for a Ridge regression (where the optimal L2 penalty is selected via a wide grid) and second for a random forest (where the optimal ensemble size is chosen from {50, 100} via 3-fold cross validation and the optimal tree depth is chosen from {2, 4, 6, 8}). Supplementary Figure 4 plots the predictive accuracy (measured with R^2) for each value of K for the two models.

We observe that the random forest is not significantly more accurate than the regression, and note a greater degree of overfitting with the random forest. We therefore select the Ridge regression, as the resulting coefficients are easier to interpret. We identify an "elbow" in the accuracy progression at K=12 features, so we use the feature subset of size $K=12$ in our PMT. These features and the weights associated with them are recorded in Supplementary Table 3.

[ii] While in the rest of this paper we use price-index adjusted per capita household consumption, in this exercise our outcome variable is raw household consumption (because the data necessary to construct price index adjusted consumption was not available to us prior to the 2020 phone survey when this analysis was performed).

4. Home Location Inference from Mobile Phone Data

We use home locations for mobile network subscribers inferred from mobile network data for a set of supplementary analyses (Supplementary Fig. 8, Supplementary Tables 9-11) and for sampling the 2020 phone survey. For the supplementary analyses, which require assigning a home prefecture and canton to each mobile network subscriber, we use standard frequency-based approaches to home location inference using the locations of cell phone towers through which subscribers place calls. These frequency-based methods have been developed in past work[46–48] and are described in more detail in Section (i) below. For sampling the 2020 phone survey, which required identifying which subscribers were likely to live in rural Novissi-eligible cantons, we developed a new approach to home location inference from mobile phone metadata using supervised learning, which is described in Section (ii) below.

i. Frequency-based home location inference

"Frequency-based" methods of home location inference, based on the locations of cell towers used by subscribers, are used widely in the literature.[46–48] Chi et al. (2020)[48] validate a set of different approaches to home location inference in comparison to ground truth location data, including the location (in our case, prefecture or canton) with the maximum phone transactions, the location with the maximum number of phone transactions in a given time frame (for example, daily between 8pm and 6am), and the location with the maximum number of unique days with phone transactions. Chi et al. (2020) find that the third method -- the maximum number of unique days with phone transactions -- is most accurate on their validation set of mobile phone metadata from Rwanda; we therefore select this approach to frequency-based home location inference. As displayed in Supplementary Table 10, this method is highly correlated with both the home prefecture and home canton recorded in voter data and with the home prefecture and home canton reported in surveys.

ii. Home location inference using machine learning

For sampling the 2020 phone survey, we were not interested in identifying the canton or prefecture each subscriber lived in; rather, we were interested in identifying which of the 5.83 million mobile network subscribers active between March and September 2020 lived in any of the 100 poorest cantons that were eligible for rural Novissi aid. This binary classification task is better suited to machine learning than the multiclass classification task of assigning subscribers to home locations; we therefore adopted a new approach to home location inference using machine learning for identifying subscribers likely to be living in the 100 poorest cantons for survey sampling.

Specifically, we trained our machine learning model on the dataset of all subscribers that registered for Novissi when it was first available in the Greater Lomé region (while only residents of Greater Lomé were eligible for this program, any registered voter in Togo could sign up for the platform for immediate eligibility in future programs). In total, this dataset includes 1.1 million subscribers with Novissi registration data matched to CDR. These registration data includes the canton in which each subscriber is registered to vote (we refer to this as the 'ground-truth' home canton).

The raw training dataset is not representative of all mobile network subscribers in Togo, as a nonrandom subset of subscribers registered for Novissi (for example, more than half of the registered subscribers are in the Greater Lomé region). To make the training data more representative, we calculated the expected share of subscribers in each canton based on the total number of voters registered in each canton and the mobile phone penetration rate in the prefecture (based on the 2018-2019 field survey). We “balanced” the training dataset by sampling observations at random from cantons with a disproportionately high number of registrants until the proportions in the training dataset reflected the expected proportion of mobile network subscribers in each canton.

Finally, we trained a machine learning model to predict whether each subscriber lived within the 100 eligible cantons. As in poverty prediction, we use a gradient boosting model with optimal hyperparameters chosen via cross-validation. The model uses the same “features” that we use for statistical home location inference – specifically the (normalized) number of unique days on which each subscriber places a transaction in each canton of Togo. The model obtains an AUC score of 0.90 and cross-validated accuracy of 93%. We then use the trained machine learning model to produce estimates of the likelihood that all 5.83 million mobile network subscribers live in an eligible canton.

5. Design of the 2020 Phone Survey

This section describes the design and implementation of the 2020 phone survey, which took place in the last week of September and the first week of October 2020.

i. Sampling

The 2020 phone survey was designed to be representative of active mobile phone subscribers living in Togo's 100 poorest cantons. The sample frame for the survey was all mobile phone subscribers active on one of the two mobile networks in Togo between March 1 and September 30, 2020 (N = 5.83 million). Sampling was based on four metrics associated with each mobile phone subscriber: inferred probability of living in a rural Novissi-eligible area, registration to a previous Novissi program, inferred wealth based on phone data, and total mobile phone expenditure.

- *Inferred probability of living in a rural Novissi-eligible canton*: We used the machine learning model described in Appendix B section (ii) to assign each subscriber a probability of living in a rural Novissi-eligible canton.

- *Registration to a previous Novissi program:* At the time of the survey, 22% of mobile network subscribers in Togo were already registered in the Novissi system, and therefore were associated with a ground-truth home canton based on the canton in which they are registered to vote. In our dataset of inferred home location likelihoods, we assigned any subscriber registered to vote in one of the 100 targeted cantons a 100% likelihood of geographical eligibility (N = 86,856). We assigned any subscriber registered to vote outside of these cantons a 0% likelihood of geographical eligibility (N = 1,046,905).

- *Inferred poverty based on mobile phone data:* We used ground-truth poverty data collected in a previous nationally-representative phone survey conducted in June 2020 to train a machine learning model to predict poverty from CDR. We followed the methods described in Methods, 'Machine Learning Methods' using the PMT as ground truth and CDR features from March 1 to September 30, 2020. We used the machine learning model to predict the poverty of each of the 5.83 million mobile phone subscribers in Togo.

- *Mobile phone expenditure:* We constructed the measure of total phone expenditure for each subscriber described in Methods, 'Parsimonious Phone Expenditure Method'.

Based on the total number of voters registered in targeted cantons and individual mobile phone penetration in each canton (based on the 2018-2019 field survey, measured at the prefecture level), we estimated that around 240,000 subscribers live in eligible cantons. We identified the 240,000 subscribers most likely to be living in a targeted canton (including all 86,856 subscribers registered in targeted cantons). Only these 240,000 subscribers were eligible to be surveyed.

We oversampled survey respondents based on two counterfactual targeting methods that we simulated pre-survey: predicted poverty based on phone data, and mobile phone expenditures, as described in Methods, 'Predicting Poverty from Phone Data'. We divided the 240,000 subscribers into four quartiles based on phone-inferred poverty and mobile phone expenditures. We overlapped the quartiles to form eight "cells", based on the combination of the two targeting

methods (for example, cell AA represents being in the lowest quartile by both targeting methods, while cell AD represents being in the lowest quartile by one method and the lowest quartile by the other, and cell BC represents being in the second-lowest quartile by one method and the second-highest quartile by the other). We assigned a cell weight of 0.20 to cells AD and BC (where the two methods disagree the most), a cell weight of 0.15 to cells AC and BD, a cell weight of 0.10 to cells AB and BC, and a cell weight of 0.05 to cells CD and DD (where the two methods disagree least).

Our sampling probabilities for the 240,000 survey-eligible subscribers were constructed as the product of a subscriber's cell weight and their probability of residing in a targeted canton (so subscribers likely to be living in targeted cantons are oversampled within each cell). The distributions of these draw probabilities are shown in Supplementary Figure 10 Panel A. We use the inverse of these draw probabilities as sample weights in our downstream analysis, in combination with response weights - see Section (iv) below. We drew 40,000 phone numbers at random from the 240,000 survey-eligible subscribers, with assigned draw probabilities. We provided these 40,000 phone numbers in a random order to enumerators with the expectation that not all of them would be called in order to reach a goal interview quota of 10,000; indeed, only 30,244 phone numbers were called before the quota was reached – see Section (ii) below.

ii. Response Rates

In total, enumerators conducted 10,701 interviews out of 30,244 phone numbers that were called (overall response rate of 35.38%). Phone numbers were called in a random order, and were assigned to enumerators by language (with random assignment with groups of enumerators speaking the same language). While we have little information on subscribers pre-survey, we can examine differential nonresponse by (1) inferred geography based on CDR, (2) registration to a previous Novissi program, and (3) pre-survey mobile phone use (we focus on the phone-predicted measure of poverty and measure of daily expenditures on calls and texts that are used in the rest of the paper). Supplementary Table 12 displays response rates disaggregated along these dimensions. We find that response rates are higher for those registered to a prior Novissi program, those inferred to be living in the regions of Lomé Commune, Maritime, or Savanes, and those with a high daily phone expenditure. Section (iv) describes how we reweight survey observations to account for differential nonresponse.

iii. Removing Low-Quality Surveys

We identified unreliable enumerators by comparing the data collected in the survey with the information contained in the Novissi registry for the subset of survey respondents who had registered to a previous Novissi program. We begin our analysis by constructing "value-added" (VA) estimates for the enumerators in our data. We predict the VA of each enumerator on the basis of the correct answers to three questions for which we obtained ground-truth information from the Novissi database (canton, age and sex), and on the frequency of surveys with a single head of household (which avoids the roster part of the survey and simplifies the enumerator's work). We control for interviewee characteristics such as region and interview language to

separate the enumerator's impact from observable interviewee selection.[iii] Our approach to estimating enumerators' VA parallels the parametric empirical Bayes estimator of teacher's VA in past work.[49–51] We then normalize the VAs for each of the four dimensions (canton, age, gender, and number of surveys with only one adult), and take the average for enumerators who conducted more than twenty interviews. The bottom ten percent of enumerators have an average VA one standard deviation below the mean VA across all enumerators; we classify their surveys as "poor quality." The interviews of the three interviewers with an average VA lower two standard deviations below the total average VA are classified as "very poor quality."

1,180 surveys associated with enumerators who are ranked "poor quality" or "very poor quality" are removed from the dataset. We drop a further 606 surveys with missing data for the PMT or one or more of the counterfactual targeting methods, for a final survey dataset size of 8,915.

iv. Reweighting for Nonresponse

As noted in section (ii), certain groups are more likely to respond to the survey than others. To make the final analysis representative of the initial sample frame (i.e., active mobile subscribers in the 100 poorest cantons) rather than just survey respondents, we reweight survey observations by likelihood of response based on pre-survey covariates.[52,53] In our case, we train a machine learning model (using an LGBM and the same set of hyperparameters used for wealth prediction from phone data) to predict response from our usual set of CDR features, along with whether a subscriber registered to a previous Novissi program. This model is trained on all 30,244 numbers that were called, with "response" defined as responding to the survey, including all questions necessary to construct the PMT and counterfactual targeting outcomes, consenting to matching between survey responses and mobile phone data, and that survey passing the quality assessment step (see Section iii), for a total "responded" population of 8,915 (29%). As in other machine learning models described in this paper, we tune hyperparameters over 5-fold cross validation and produce predictions for each observation over 10-fold cross validation. The model achieves a cross-validated AUC score of 0.71; feature importances for the model are shown in Supplementary Table 13. To assess the model's accuracy, Supplementary Figure 11 compares binned estimates of response probability with true rates of response, and indicates that the response prediction model is well-calibrated. Supplementary Figure 10 Panel B displays the distribution of response probabilities for observations included in the final survey dataset.

The final survey weights used in the paper are the product of the inverse of the response probability and the inverse of the sampling probability described in Section (i); the distribution of survey weights are shown in Supplementary Figure 10 Panel C.

v. Survey Content

Surveys lasted 30 minutes on average, and included questions on the demographics of the respondent and household members, assets owned by the household, subjective wellbeing of the

[iii] As the phone number list was randomized and then distributed to the enumerators, we believe there is little room for sorting.

respondent, the social services available to the household, and the impacts of COVID-19 on the household. The full survey instrument is publicly available online.[iv]

[iv] https://jblumenstock.com/files/papers/TogoInstrument2020.pdf

References

1. Hanna, R. & Olken, B. A. Universal Basic Incomes versus Targeted Transfers: Anti-Poverty Programs in Developing Countries. *Journal of Economic Perspectives* **32**, 201–226 (2018).
2. Ravallion, M. *The Economics of Poverty: History, Measurement, and Policy*. (Oxford University Press, 2016).
3. Nichols, A. L. & Zeckhauser, R. J. Targeting Transfers through Restrictions on Recipients. *The American Economic Review* **72**, 372–377 (1982).
4. Alatas, V. *et al.* Self-Targeting: Evidence from a Field Experiment in Indonesia. *Journal of Political Economy* **124**, 371–427 (2016).
5. Kleven, H. J. & Kopczuk, W. Transfer Program Complexity and the Take-Up of Social Benefits. *American Economic Journal: Economic Policy* **3**, 54–90 (2011).
6. Baker, J. L. & Grosh, M. E. Poverty reduction through geographic targeting: How well does it work? *World Development* **22**, 983–995 (1994).
7. Schady, N. R. Picking the Poor: Indicators for Geographic Targeting in Peru. *Review of Income and Wealth* **48**, 417–433 (2002).
8. Coady, D. P. The Welfare Returns to Finer Targeting: The Case of The Progresa Program in Mexico. *Int Tax Public Finan* **13**, 217–239 (2006).
9. Elbers, C., Fujii, T., Lanjouw, P., Özler, B. & Yin, W. Poverty alleviation through geographic targeting: How much does disaggregation help? *Journal of Development Economics* **83**, 198–213 (2007).
10. Smythe, I. & Blumenstock, J. E. Geographic Micro-Targeting of Social Assistance with High-Resolution Poverty Maps. in *In Submission (KDD)* (2021).
11. Grosh, M. E. & Baker, J. L. *Proxy means tests for targeting social programs*. (The World Bank, 1995). doi:10.1596/0-8213-3313-5.
12. Filmer, D. & Pritchett, L. H. Estimating Wealth Effects Without Expenditure Data—Or Tears: An Application To Educational Enrollments In States Of India*. *Demography* **38**, 115–132 (2001).
13. Desiere, S., Vellema, W. & D'Haese, M. A validity assessment of the Progress out of Poverty Index (PPI)™. *Evaluation and Program Planning* **49**, 10–18 (2015).
14. Brown, C., Ravallion, M. & van de Walle, D. A poor means test? Econometric targeting in Africa. *Journal of Development Economics* **134**, 109–124 (2018).
15. Alderman, H. Do local officials know something we don't? Decentralization of targeted transfers in Albania. *Journal of Public Economics* **83**, 375–404 (2002).
16. Galasso, E. & Ravallion, M. Decentralized targeting of an antipoverty program. *Journal of Public Economics* **89**, 705–727 (2005).
17. Premand, P. & Schnitzer, P. Efficiency, Legitimacy, and Impacts of Targeting Methods: Evidence from an Experiment in Niger. *The World Bank Economic Review* (2020) doi:10.1093/wber/lhaa019.
18. Mann, L. Left to Other Peoples' Devices? A Political Economy Perspective on the Big Data Revolution in Development. *Development and Change* **49**, 3–36 (2018).
19. Kerry, C. F., Kendall, J. & de Montjoye, Y.-A. Enabling Humanitarian Use of Mobile Phone Data. *Brookings Issues in Technology Innovation* (2014).
20. Blumenstock, J. E. Don't forget people in the use of big data for development. *Nature* **561**, 170–172 (2018).

21. Ohlenberg, T. *AI in Social Protection – Exploring Opportunities and Mitigating Risks*. (Deutsche Gesellschaft für Internationale Zusammenarbeit (GIZ), 2020).
22. Abebe, R. *et al.* Narratives and Counternarratives on Data Sharing in Africa. in *Proceedings of the 2021 ACM Conference on Fairness, Accountability, and Transparency* 329–341 (Association for Computing Machinery, 2021). doi:10.1145/3442188.3445897.
23. Taylor, L. No place to hide? The ethics and analytics of tracking mobility using mobile phone data. *Environ Plan D* **34**, 319–336 (2016).
24. Necessary and Proportionate. International Principles on the Application of Human Rights to Communications Surveillance. (2013).
25. Taylor, L. No place to hide? The ethics and analytics of tracking mobility using mobile phone data. *Environ Plan D* **34**, 319–336 (2016).
26. Alaggan, M., Gambs, S., Matwin, S. & Tuhin, M. Sanitization of Call Detail Records via Differentially-Private Bloom Filters. in *Data and Applications Security and Privacy XXIX* (ed. Samarati, P.) vol. 9149 223–230 (Springer International Publishing, 2015).
27. Mir, D., Isaacman, S., Caceres, R., Martonosi, M. & Wright, R. DP-WHERE: Differentially private modeling of human mobility. in 580–588 (2013). doi:10.1109/BigData.2013.6691626.
28. Li, T., Sahu, A. K., Talwalkar, A. & Smith, V. Federated Learning: Challenges, Methods, and Future Directions. *IEEE Signal Processing Magazine* **37**, 50–60 (2020).
29. Oliver, N. *et al.* Mobile phone data for informing public health actions across the COVID-19 pandemic life cycle. *Science Advances* eabc0764 (2020) doi:10.1126/sciadv.abc0764.
30. Taylor, L. & Broeders, D. In the name of Development: Power, profit and the datafication of the global South. *Geoforum* **64**, 229–237 (2015).
31. Cate, F. H. & Mayer-Schönberger, V. Notice and consent in a world of Big Data. *International Data Privacy Law* **3**, 67–73 (2013).
32. Ioannidis, J. P. A. Informed Consent, Big Data, and the Oxymoron of Research That Is Not Research. *The American Journal of Bioethics* **13**, 40–42 (2013).
33. Noriega-Campero, A. *et al.* Algorithmic targeting of social policies: fairness, accuracy, and distributed governance. in *Proceedings of the 2020 Conference on Fairness, Accountability, and Transparency* 241–251 (ACM, 2020). doi:10.1145/3351095.3375784.
34. Kleinberg, J., Mullainathan, S. & Raghavan, M. Inherent Trade-Offs in the Fair Determination of Risk Scores. *arXiv:1609.05807 [cs, stat]* (2016).
35. Goodhart, C. *Monetary Relationships: A View from Threadneedle Street*. (University of Warwick, 1975).
36. Akerlof, G. A. The economics of 'tagging' as applied to the optimal income tax, welfare programs, and manpower planning. *The American economic review* **68**, 8–19 (1978).
37. Martinelli, C. & Parker, S. W. Deception and Misreporting in a Social Program. *Journal of the European Economic Association* **7**, 886–908 (2009).
38. Camacho, A. & Conover, E. Manipulation of Social Program Eligibility. *American Economic Journal: Economic Policy* **3**, 41–65 (2011).
39. Banerjee, A., Hanna, R., Olken, B. A. & Sumarto, S. *The (lack of) Distortionary Effects of Proxy-Means Tests: Results from a Nationwide Experiment in Indonesia*. http://www.nber.org/papers/w25362 (2018) doi:10.3386/w25362.
40. Finkelstein, A. E-Z Tax: Tax Salience and Tax Rates. *The Quarterly Journal of Economics* **124**, 969–1010 (2009).

41. Björkegren, D., Blumenstock, J. E. & Knight, S. Manipulation-Proof Machine Learning. *arXiv:2004.03865 [cs, econ]* (2020).
42. Egger, D., Haushofer, J., Miguel, E., Niehaus, P. & Walker, M. W. *General Equilibrium Effects of Cash Transfers: Experimental Evidence from Kenya*. https://www.nber.org/papers/w26600 (2019) doi:10.3386/w26600.
43. Cunha, J. M., De Giorgi, G. & Jayachandran, S. The Price Effects of Cash Versus In-Kind Transfers. *The Review of Economic Studies* **86**, 240–281 (2019).
44. Geng, X., Janssens, W., Kramer, B. & List, M. Health insurance, a friend in need? Impacts of formal insurance and crowding out of informal insurance. *World Development* **111**, 196–210 (2018).
45. Mobarak, A. M. & Rosenzweig, M. *Selling formal insurance to the informally insured*. https://www.econstor.eu/handle/10419/59144 (2012).
46. Blumenstock, J. E., Cadamuro, G. & On, R. Predicting poverty and wealth from mobile phone metadata. *Science* **350**, 1073–1076 (2015).
47. Vanhoof, M., Reis, F., Ploetz, T. & Smoreda, Z. Assessing the Quality of Home Detection from Mobile Phone Data for Official Statistics. *Journal of Official Statistics* **34**, 935–960 (2018).
48. Chi, G., Lin, F., Chi, G. & Blumenstock, J. A general approach to detecting migration events in digital trace data. *PLoS One* **15**, e0239408 (2020).
49. Kane, T. J. & Staiger, D. O. *Estimating Teacher Impacts on Student Achievement: An Experimental Evaluation*. https://www.nber.org/papers/w14607 (2008) doi:10.3386/w14607.
50. Chetty, R., Friedman, J. N. & Rockoff, J. E. Measuring the Impacts of Teachers I: Evaluating Bias in Teacher Value-Added Estimates. *American Economic Review* **104**, 2593–2632 (2014).
51. Gilraine, M., Gu, J. & McMillan, R. *A New Method for Estimating Teacher Value-Added*. https://www.nber.org/papers/w27094 (2020) doi:10.3386/w27094.
52. Kalton, G. & Flores Cervantes, I. Weighting Methods. *http://lst-iiep.iiep-unesco.org/cgi-bin/wwwi32.exe/[in=epidoc1.in]/?t2000=019622/(100)* **19**, (2003).
53. Buskirk, T. D. & Kolenikov, S. Finding Respondents in the Forest: A Comparison of Logistic Regression and Random Forest Models for Response Propensity Weighting and Stratification. *Survey Methods: Insights from the Field (SMIF)* (2015) doi:10.13094/SMIF-2015-00003.
54. Tiecke, T. G. *et al.* Mapping the world population one building at a time. *arXiv:1712.05839 [cs]* (2017).

Supplementary Figures

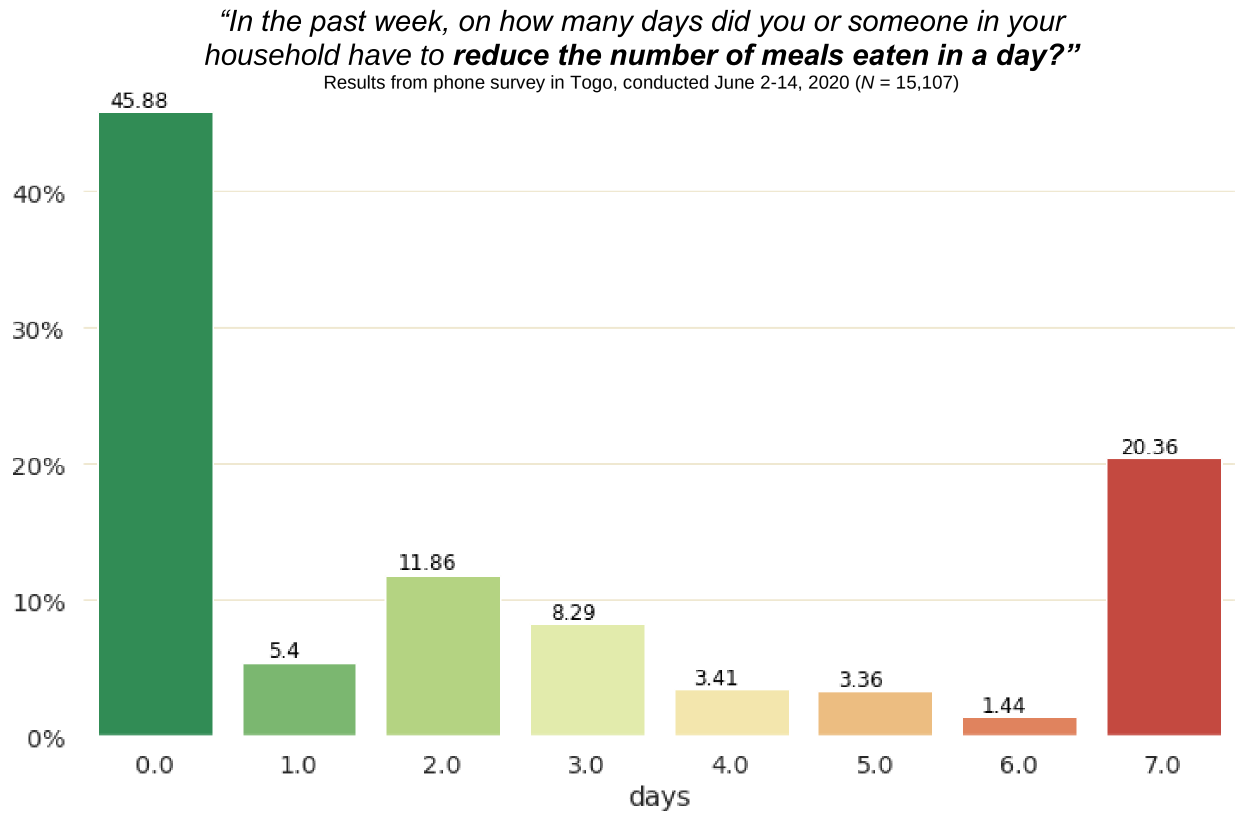

Supplementary Figure 1 | Food insecurity in Togo. In June 2020, we conducted a phone survey of 15,107 mobile phone owners in Togo. Survey weights are used to make responses representative of the population of mobile phone owners in Togo.

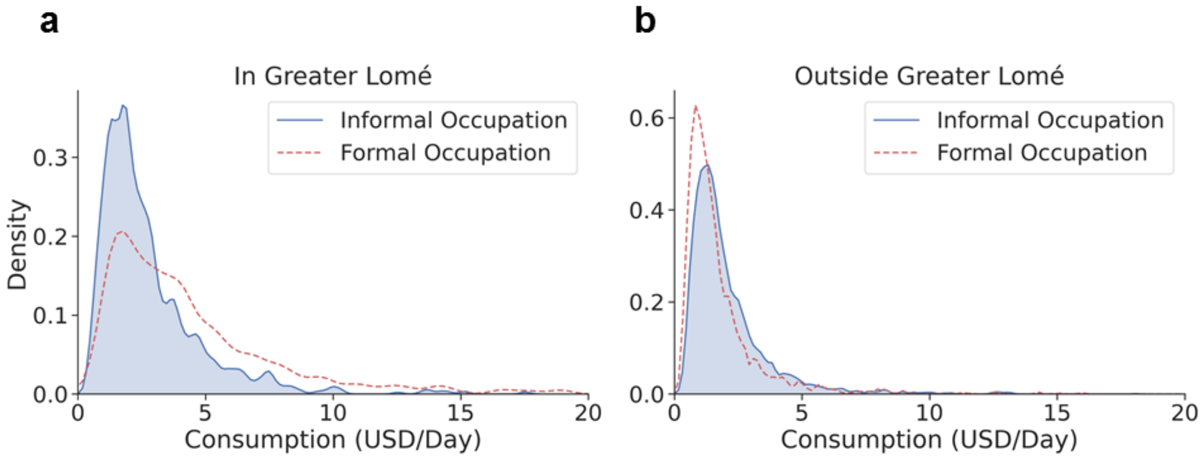

Supplementary Figure 2 | Wealth of formal vs. informal workers. Results based on analysis of nationally-representative household survey data collected by the Government of Togo in 2018-2019 (*N* = 6,171). Data is collected at the household-level, we assign a household-level informal occupation indicator if at least one of the adult household members is unemployed or employed in an informal occupation. See Methods, 'Data Sources'.

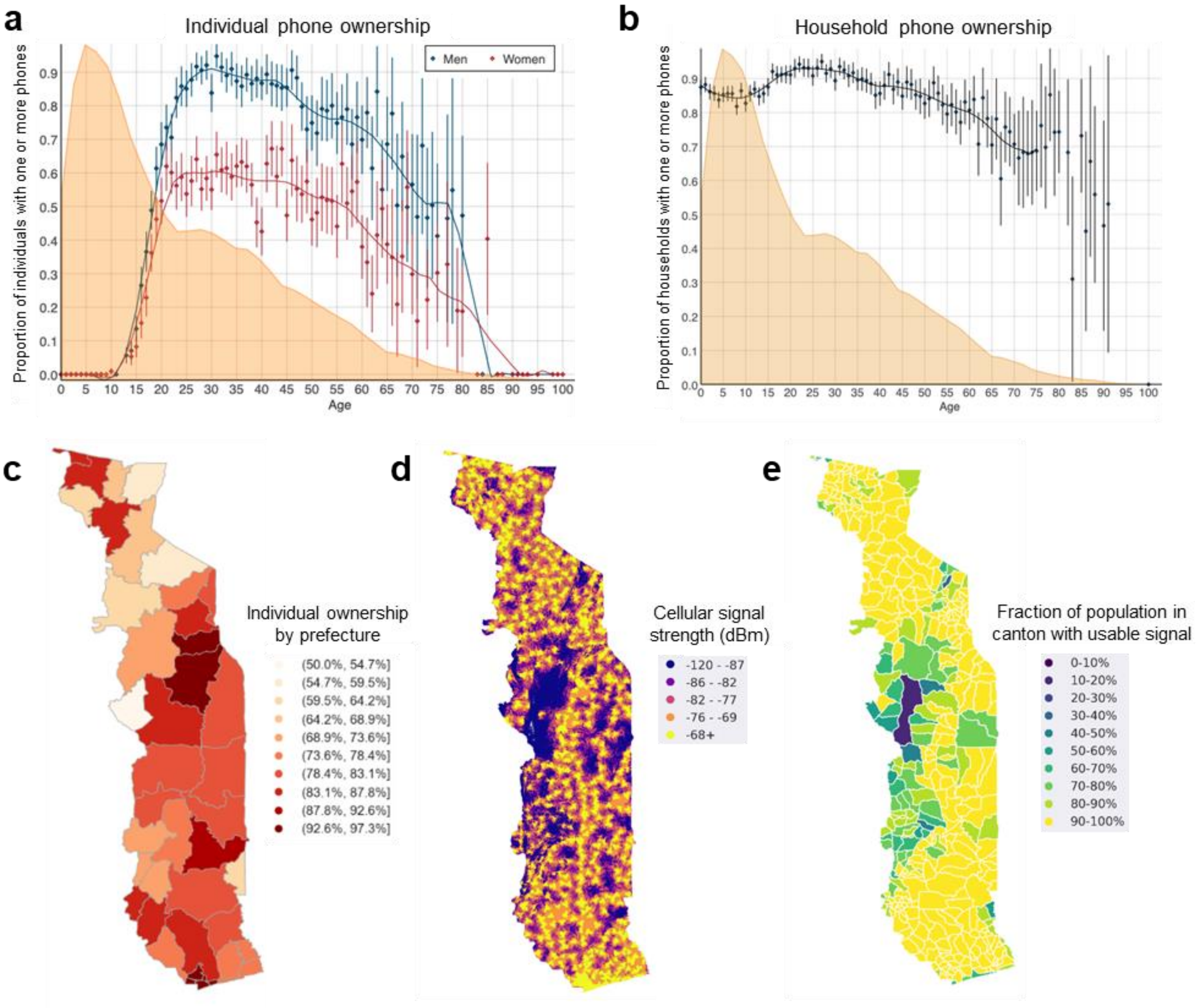

Supplementary Figure 3 | Mobile phone penetration and coverage in Togo. Based on nationally-representative household survey data collected in 2018-2019, we estimate **a)** the percentage of adults in Togo with one or more mobile phone, disaggregated by age and gender (the dots indicate the sample mean, while vertical bands indicate 95% confidence intervals derived from N=27,483 total individual survey responses); **b)** the percentage of households in Togo with one or more mobile phones, disaggregated by the age of the head of household (the dots indicate the sample mean, while vertical bands indicate 95% confidence intervals derived from N=27,483 total individual survey responses); and **c)** the percentage of individuals in each prefecture with one or more mobile phones. Using data on the location and signal strength of all cell towers in Togo, made available by Togocel (one of the two phone companies in Togo), we calculate **d)** the signal strength across Togo; and **e)** the fraction of the population in each canton with access to a usable signal, where signal greater than -86 dBm is generally considered usable, and sub-canton estimates of population density are derived from satellite imagery and downloaded from the Humanitarian Data Exchange[54].

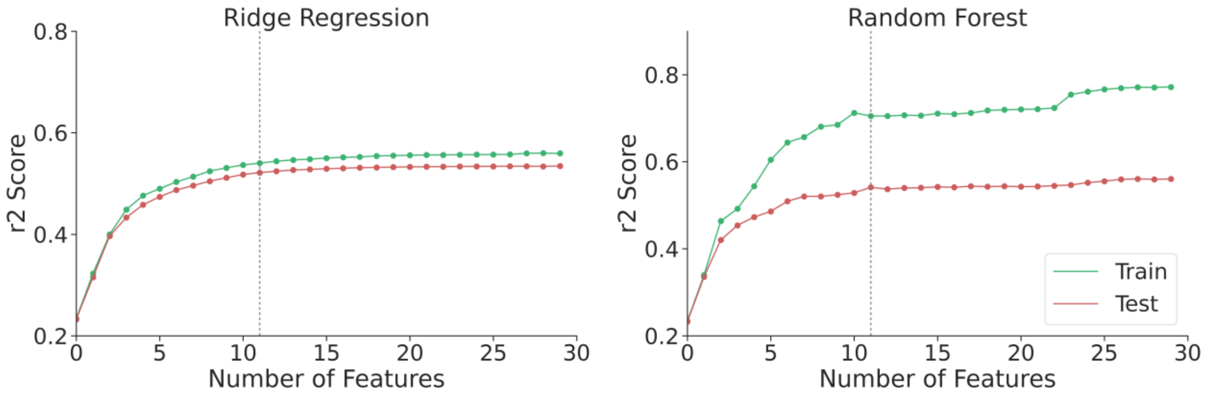

Supplementary Figure 4 | Selection of variables for proxy-means test. Each plot shows the accuracy (measured by r^2 score) of a proxy-means test using the most predictive feature subset of size *K*, where *K* is plotted on the x-axis. The left plot shows the accuracy obtained by a Ridge regression; the right plot shows the accuracy obtained by a random forest. Feature subsets are selected via stepwise forward selection.

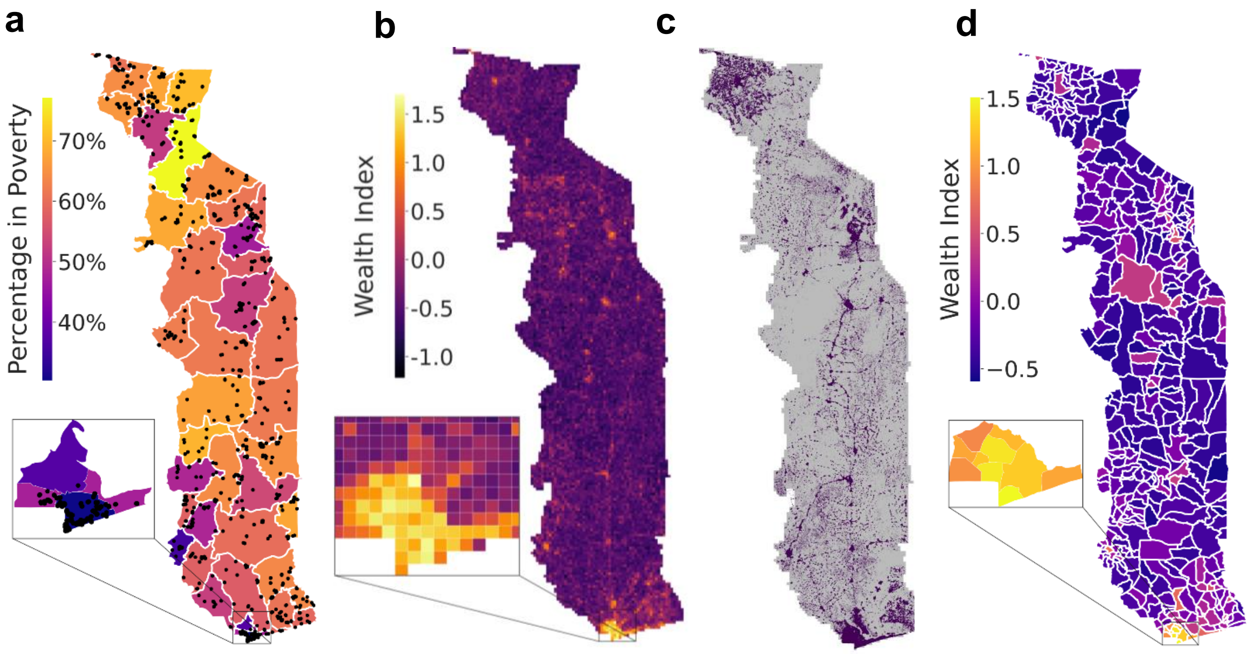

Supplementary Figure 5 | Poverty maps. (a) Prefecture (admin-2) poverty map inferred from 2017 field survey (N = 26,902), showing the percent of the population living under the poverty line by prefecture. Overlayed with locations of survey observations in black points. **(b)** High-resolution estimates of consumption derived from satellite imagery. **(c)** High-resolution estimates of population density derived from satellite imagery. **(d)** Canton (admin-3) poverty map inferred from satellite imagery by combining high-resolution consumption estimates and population density estimates to calculate weighted average consumption per canton.

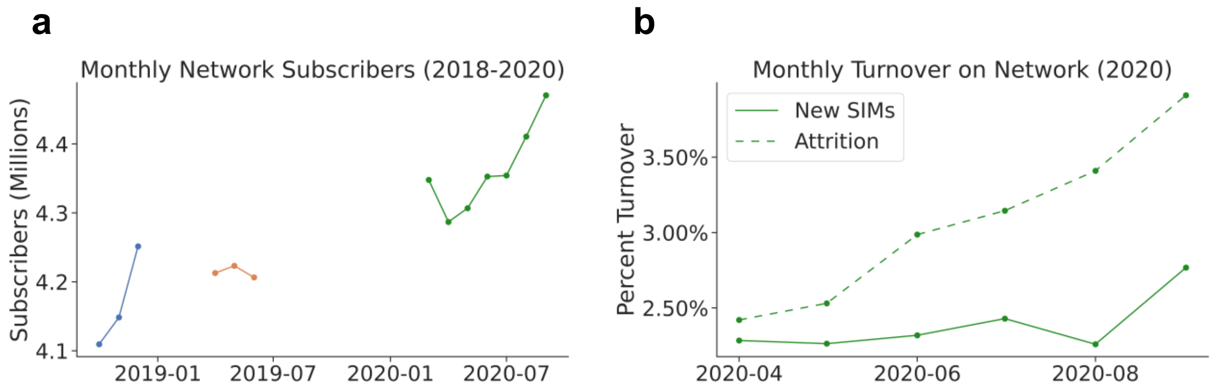

Supplementary Figure 6 | Mobile phone network activity. **(a)** Count of unique subscribers making at least one outgoing transaction (call or text) on the mobile network in each month. October-December 2018 shown in blue, April-June 2019 in orange, and March-September 2020 in green. **(b)** Monthly turnover from the network in April-September 2020. New SIMs are quantified as the proportion of subscribers in each month whose first observed transaction is in that month. Attrition is quantified as the proportion of subscribers in each month who make no further outgoing transactions after that month. Note that we do not observe CDR in the months prior to March 2020, so we show results starting in April 2020 in Panel B; nonetheless a small proportion of the new SIMs in Panel B are inevitably due to sparsity in the CDR (that is, subscribers who placed a transaction prior to March 2020 that is not recorded in our dataset). Likewise, we do not observe CDR past December 2020, so a small part of the attrition measured in Panel B is due to sparsity in CDR transactions.

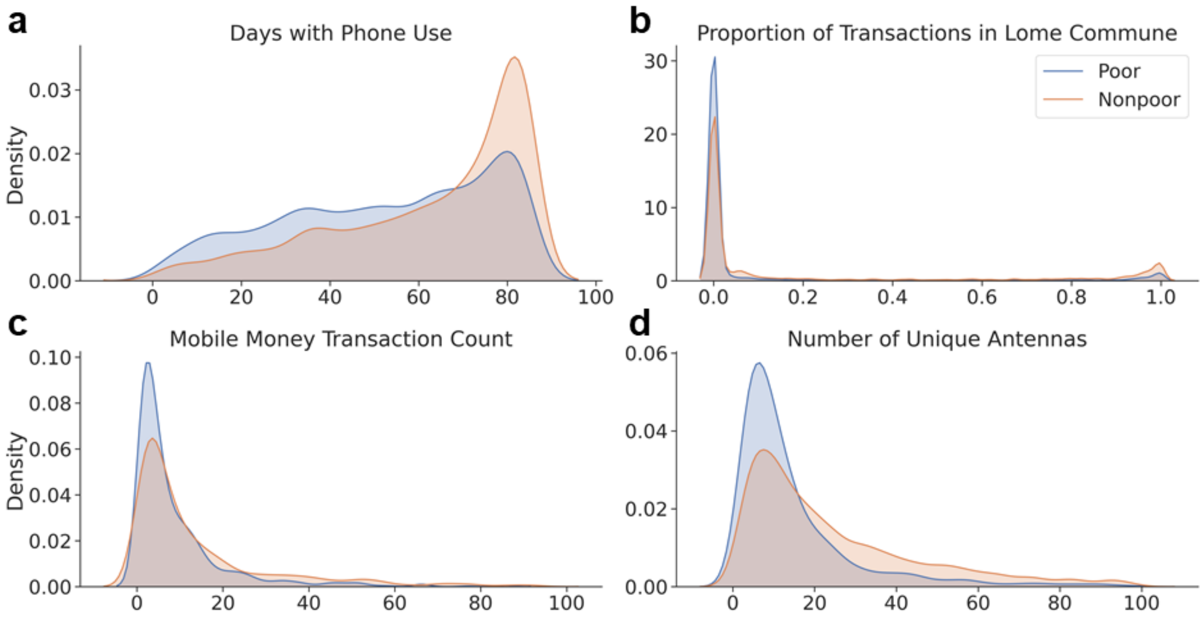

Supplementary Figure 7 | CDR features. Comparing the distribution for CDR features for those above and below the international poverty line (USD 1.90/day) in the 2018-2019 field survey dataset.

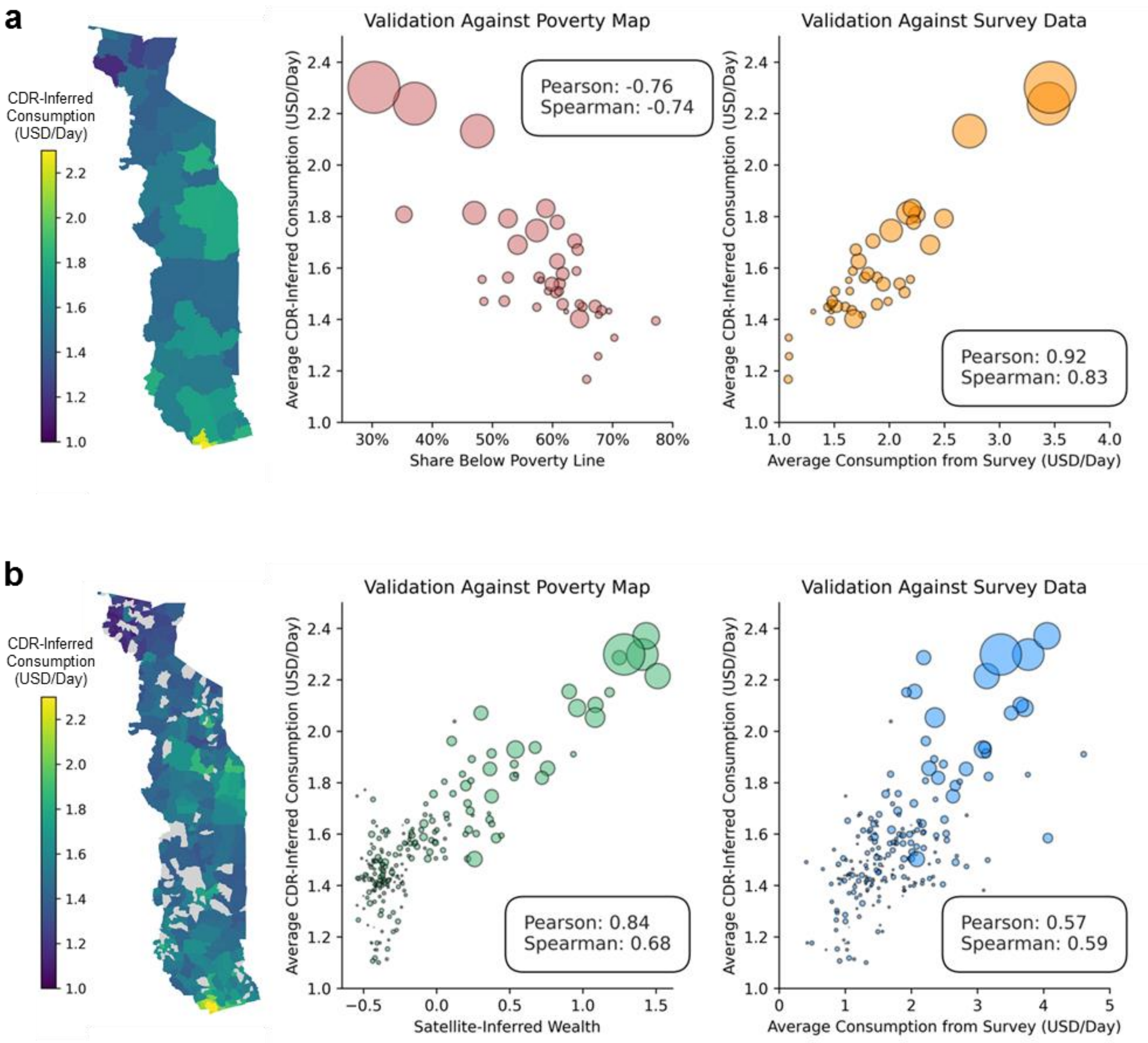

Supplementary Figure 8 | Spatial validation of phone-based poverty predictions. a) Map shows average phone-inferred consumption of subscribers in each *prefecture* (using CDR-based predictions trained on the 2018-19 in-person survey). Scatter plots compare average prefecture consumption, as derived from CDR (shown on y-axis), against two measures of poverty derived from the 2018-19 in-person survey (shown on x-axis): the share of people in the prefecture below the poverty line (middle plot), and the average consumption of households in the prefecture (right plot). **b)** Map shows average phone-inferred consumption of subscribers in each *canton* (cantons with no associated subscribers are shown in grey). Scatter plots compare average consumption per canton from the 2018-19 phone survey (evaluated across the 75% of all cantons in which there are observations in the 2018-19 field survey). Bubbles are sized by the number of subscribers assigned to each prefecture/canton.

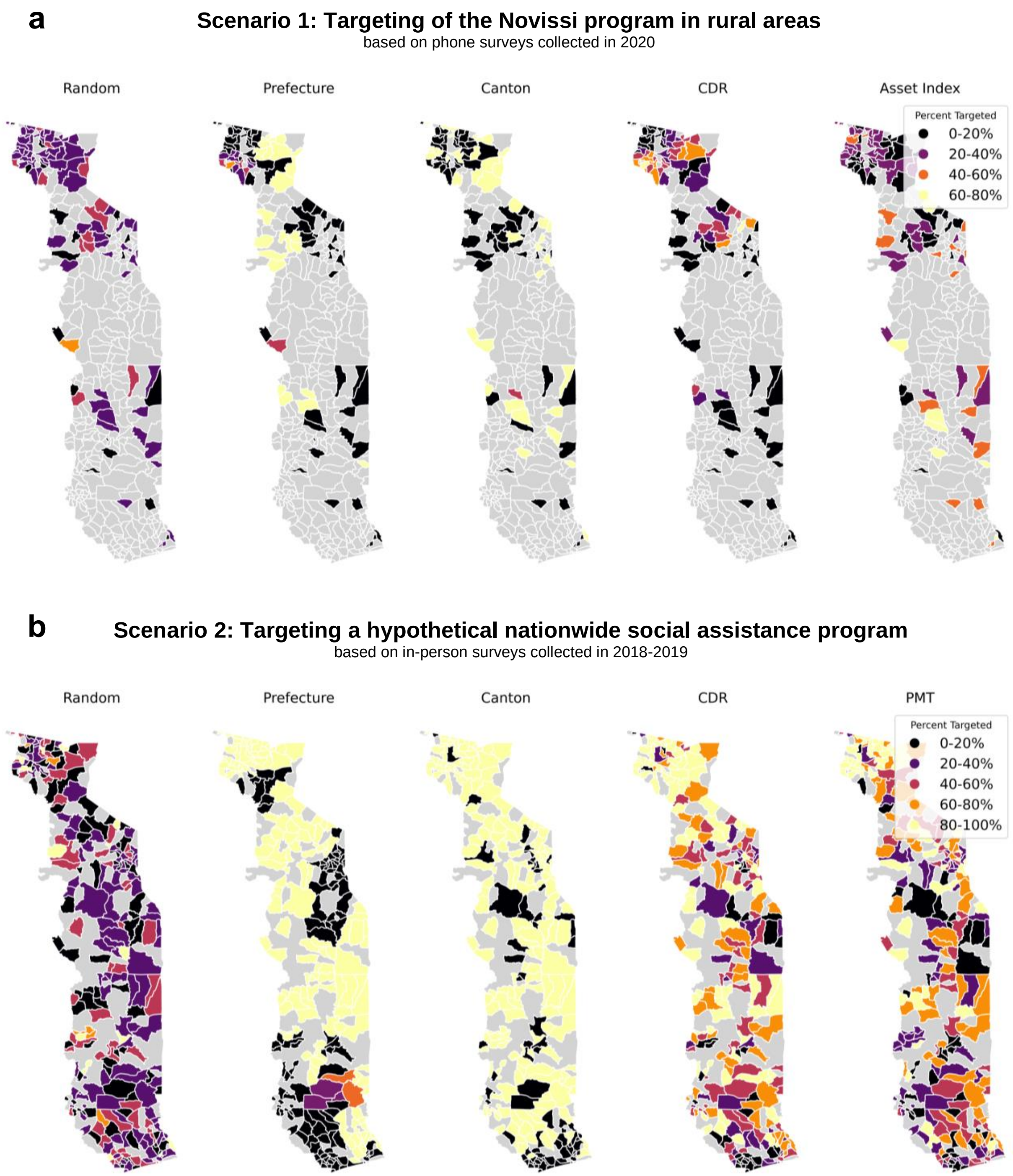

Supplementary Figure 9 | Share targeted by canton by different targeting methods. Panel A: Targeting share for the Novissi program in rural Togo, evaluated using individuals from the 2020 phone survey who report living in one of the 100 eligible cantons (N = 6,745). The respondent's self-reported canton and prefecture are used to color the map. Panel B: Targeting share for the hypothetical nationwide program, using data from the 2018-19 national household survey. Note that certain cantons have no observations in the 2018-2019 survey; these are shown in grey in Panel B. Cantons outside of the 100 poorest are shown in grey in Panel A.

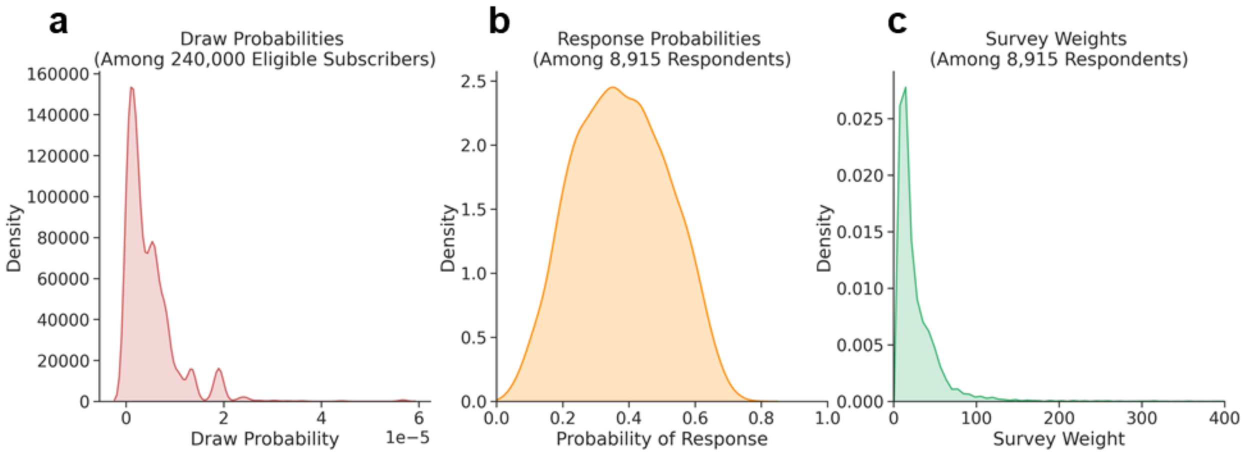

Supplementary Figure 10 | Distribution of sample weights for 2020 phone survey. Panel A: Distribution of draw probabilities among subscribers eligible for the survey. Panel B: Distribution of response probabilities for observations included in the final survey dataset, based on the response prediction model. Panel C: Distribution of sample weights (product of the inverse of the draw probability and the inverse of the response probability) for observations included in the final survey dataset.

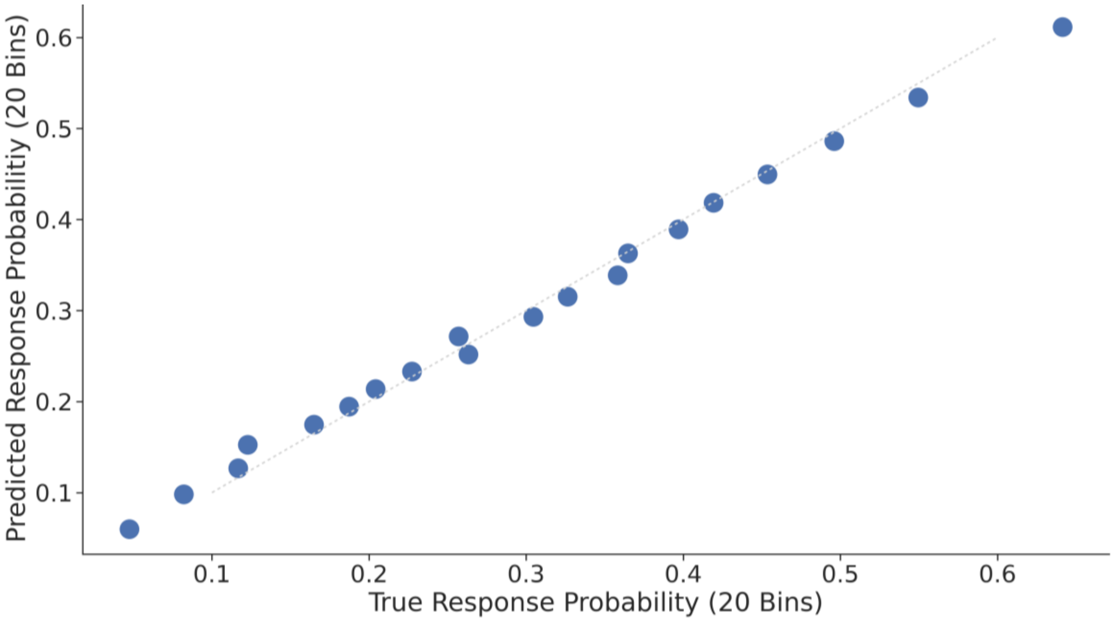

Supplementary Figure 11 | Calibration of response probabilities for 2020 phone survey. We compare the predicted probability of response (y-axis, binned into 20 quantiles) to the realized probability of response (x-axis, again binned into 20 quantiles) to confirm that the response prediction model is well-calibrated.

Supplementary Tables

Targeting a hypothetical nationwide program – but only in rural areas
Based on 2018 Phone Survey Restricted to Rural Areas (*N* = 2,306)

	Spearman	AUC	Accuracy	Precision & Recall
Panel A: Targeting methods considered by the Government of Togo in 2020				
Prefecture (Admin-2 regions)	0.16 (0.023)	0.57 (0.011)	64% (0.97%)	37% (1.67%)
Canton (Admin-3 regions)	0.19 (0.025)	0.59 (0.013)	63% (0.98%)	36% (1.69%)
Phone (Expenditures)	0.15 (0.024)	0.59 (0.012)	63% (1.05%)	36% (1.81%)
Phone (Machine Learning)	0.30 (0.023)	0.65 (0.012)	67% (1.00%)	43% (1.73%)
Panel B: Common alternative targeting methods that could not be implemented in Togo in 2020				
Asset Index	0.36 (0.023)	0.68 (0.011)	67% (1.01%)	44% (1.74%)
PPI	0.55 (0.017)	0.77 (0.009)	72% (1.07%)	52% (1.84%)
PMT	0.61 (0.016)	0.80 (0.007)	73% (1.06%)	54% (1.84%)
Rural PMT	0.52 (0.018)	0.75 (0.008)	72% (1.02%)	51% (1.75%)
Panel C: Additional counterfactual targeting methods that were feasible in Togo in 2020				
Random	0.00 (0.024)	0.50 (0.012)	59% (1.04%)	29% (1.79%%)
Occupation (Novissi)	-0.13 (0.024)	0.44 (0.011)	54% (0.89%)	21% (1.53%)
Occupation (Optimal)	0.31 (0.023)	0.63 (0.010)	63% (0.62%)	37% (1.06%)

Supplementary Table 1 | Performance of targeting the hypothetical national program, when restricted to rural areas. Analysis is similar to that presented in the last four columns of Table 1, but analysis is restricted to the 2,306 survey respondents (of the 4,171 total) who live in rural areas.

Asset	Magnitude (2018-2019 Field Survey)	Magnitude (2020 Phone Survey)
Electricity access	0.38	
Toilet	0.37	0.41
TV	0.35	
Electricity grid	0.35	
Garbage disposal	0.33	
Waste disposal	0.33	
Iron	0.26	0.06
Radio	0.20	0.23
Clean water (wet season)	0.16	
Clean water (dry season)	0.16	
Refrigerator	0.12	0.02
Walls	0.12	
Floor	0.11	
Mobile phone	0.11	
Water disposal	010	
Motorcycle	0.10	0.88
Computer	0.09	0.02
Roof	0.08	
Stove	0.07	0.06
Car	0.06	0.00
Tablet	0.01	0.00
Air conditioner	0.01	0.00
House	0.00	
Electricity (offgrid)	0.00	

Supplementary Table 2 | Asset-based wealth index. Magnitude of first principal component for 2018-2019 field survey and 2020 phone survey.

Feature	β	Feature (continued)	β
Car	2.77	HHW Education 4	-0.18
Stove	1.77	Pref. Lacs	-0.18
Refrigerator	1.32	Pref. Sotouboua	-0.18
HHH Education 8	1.12	Pref. Kloto	-0.21
HHH Education 9	0.91	HHW Education 6	-0.21
HHH Hospitalization	0.81	Pref. Kpele	-0.21
Iron	0.63	Pref. Bas-Mono	-0.23
HHH Education 3	0.55	Pref. Lome Commune	-0.23
TV	0.50	Pref. Danyi	-0.24
All children in school	0.48	Pref. Yoto	-0.26
Pref. Cinkasse	0.39	Pref. Agoe-Nyive	-0.27
Pref. Tchamba	0.33	HHH Education 5	-0.27
Toilet	0.26	No children in school	-0.31
HHH Education 7	0.17	Pref. Assoli	-0.32
Pref. Est-Mono	0.14	Pref. Kpendjal-Ouest	-0.33
HHW Education 0	0.12	Pref. Zio	-0.33
Pref. Tchaoudjo	0.09	Pref. Amou	-0.34
Pref. Bassar	0.09	HHW Education 3	-0.34
Pref. Haho	0.07	Pref. Plaine du Mo	-0.34
Pref. Dankpen	0.04	Pref. Anie	-0.34
Pref. Moyen-Mono	-0.03	Pref. Tandjoare	-0.35
Pref. Oti-Sud	-0.06	Pref. Binah	-0.37
Pref. Oti	-0.08	Pref. Ave	-0.39
Pref. Wawa	-0.11	Pref. Keran	-0.41
Pref. Vo	-0.11	Pref. Kpendjal	-0.46
Pref. Ogou	-0.12	HHW Education 2	-0.50
Pref. Tone	-0.14	Pref. Kozah	-0.51
Pref. Agou	-0.15	HHH Education 2	-0.57
Pref. Akebou	-0.17	Pref. Blitta	-0.61
HHW Education 1	-0.17	HHH Education 1	-0.63
Some children in school	-0.17	Pref. Golfe	-0.68
Number of children	-0.17	Pref. Doufelgou	-0.75

Supplementary Table 3 | Proxy means test. Weights for linear model, trained on 2018-2019 field survey (N = 6,171).

Feature	β	Feature (continued)	β
Refrigerator	0.38	Pref. Akebou	0.03
HHH Hospitalization	0.32	Pref. Ogou	0.02
Motorcycle	0.31	Pref. Ave	-0.01
TV	0.28	Pref. Moyen-Mono	-0.03
Pref. Vo	0.26	Number of children	-0.08
Computer	0.24	Pref. Plaine du Mo	-0.08
Pref. Tchamba	0.21	Pref. Est-Mono	-0.10
Garbage removal	0.17	Pref. Dankpen	-0.12
Pref. Wawa	0.17	Pref. Binah	-0.13
Toilet	0.16	Pref. Tchaoudjo	-0.13
Pref. Kloto	0.16	Pref. Cinkasse	-0.14
Pref. Haho	0.16	Pref. Oti-Sud	-0.15
Pref. Yoto	0.14	Pref. Anie	-0.15
Pref. Bas-Mono	0.14	Pref. Oti	-0.18
Pref. Golfe	0.14	Pref. Kozah	-0.19
Pref. Kpele	0.14	Pref. Tone	-0.22
Pref. Lacs	0.13	Pref. Assoli	-0.22
Floor of solid materials	0.10	Pref. Blitta	-0.23
Pref. Zio	0.10	No children in school	-0.24
Pref. Lome Commune	0.09	Some children in school	-0.28
Pref. Agou	0.09	Pref. Doufelgou	-0.29
Roof of solid materials	0.08	Pref. Kpendjal-Ouest	-0.32
Pref. Bassar	0.08	Pref. Keran	-0.33
Pref. Amou	0.06	Pref. Kpendjal	-0.35
Pref. Danyi	0.06	Pref. Tandjoare	-0.40
Pref. Soutoubua	0.05		

Supplementary Table 4 | Rural-specific proxy means test. Weights for linear model, trained on 2018-2019 phone survey restricted to rural areas (N = 3,895).

	2018-2019 Field Survey (N=6,171)			*2020 Phone Survey (N=8,915)*	
	Consumption	Proportion	N	Proportion	N
Intellectual Professions	$4.11 (3.55)	7%	277	7%	577
Intermediate Professions	$3.95 (3.56)	5%	197	3%	264
Administrators	$3.89 (3.57)	1%	32	0%	16
Managers and Directors	$3.70 (3.03)	3%	106	0%	36
Unemployed/Unknown	$3.19 (2.44)	8%	339	3%	275
Direct Services and Merchants	$2.75 (2.11)	23%	940	28%	2,111
Industry/Artisans	$2.47 (1.83)	15%	587	12%	1,026
Military Professions	$2.45 (1.25)	0%	17	1%	26
Elementary Professions	$2.21 (1.83)	2%	65	3%	249
Factory Workers	$2.17 (1.44)	7%	267	2%	165
Agricultural Professions	$1.53 (0.94)	29%	1,744	41%	4,170

Supplementary Table 5 | Occupation categories. Average daily per capita consumption per occupation category, with counts by category, separately for the 2018-2019 field survey and 2020 phone survey. Occupation categories for the 2018-2019 survey are for the household head, for the 2020 survey are for the individual respondent.

	2018-2019 National Household Survey					2020 Phone Survey
	Full Survey	Phone Number	No Phone Number	Phone Number, Matched	Phone Number, Unmatched	Full Survey
Consumption	2.39<br>(2.41)	2.56<br>(2.38)	1.75<br>(2.41)	2.59<br>(2.42)	2.21<br>(1.78)	*[data not available]*
PMT	2.10<br>(1.43)	2.22<br>(1.47)	1.65<br>(1.16)	2.23<br>(1.47)	2.03<br>(1.38)	1.62<br>(0.72)
Occupation<br>(% Formal)	56.42%<br>(49.59%)	51.98%<br>(49.96%)	72.94%<br>(44.43%)	51.28%<br>(49.99%)	59.63%<br>(49.08%)	66.54%<br>(47.19%)
% Rural	51.93%<br>(49.96%)	45.17%<br>(49.77%)	77.12%<br>(42.01%)	43.79%<br>(49.61%)	60.17%<br>(48.97%)	96.19%<br>(19.15%)
% Women	28.15%<br>(44.98%)	23.61%<br>(42.47%)	45.07%<br>(49.76%)	23.43%<br>*42.36%)	25.63%<br>(43.68%)	23.27%<br>(42.25%)
Age	43.97<br>(14.43)	41.96<br>(13.19)	51.26<br>(16.28%)	41.97<br>(13.15%)	41.84<br>*(13.71%)	33.20<br>(11.90)
N	6,089	4,571	1,518	4,171	400	8,915

Supplementary Table 6 | Summary statistics for two survey datasets. Means and standard deviations for key outcomes in the 2018-2019 national household survey (*N* = 6,089) and 2020 phone survey concentrated in the 100 poorest cantons (*N* = 8,915). For the 2018-2019 national household survey, we break down the sample into two groups: households that provided enumerators with a phone numbers (*N* = 4,571) and those that do not (*N* = 1,518). We further break down the sample providing a phone number into two groups: households for which the phone number appears in data obtained from the mobile network operators (*N* = 4,171) and those for which it does not (*N* = 400). For the 2018-19 phone survey, occupation, gender, and age are assigned based on the head of household; for the 2020 phone survey they are assigned based on the respondent.

Targeting a hypothetical nationwide program – with PMT as ground truth
Based on 2018-2019 National Household Survey (*N* = 4,171)

	Spearman	AUC	Accuracy	Precision & Recall
Panel A: Targeting methods considered by the Government of Togo in 2020				
Prefecture	0.50	0.73	72%	51%
(Admin-2 regions)	(0.014)	(0.006)	(0.73%)	(1.25%)
Canton	0.54	0.73	74%	55%
(Admin-3 regions)	(0.013)	(0.006)	(0.70%)	(1.22%)
Phone	0.31	0.64	66%	41%
(Expenditures)	(0.017)	(0.008)	(0.75%)	(1.30%)
Phone	0.56	0.78	73%	54%
(Machine Learning)	(0.014)	(0.006)	(0.73%)	(1.25%)
Panel B: Common alternative targeting methods that could not be implemented in Togo in 2020				
Asset Index	0.68	0.82	77%	60%
	(0.010)	(0.005)	(0.73%)	(1.26%)
PPI	0.74	0.86	81%	67%
	(0.009)	(0.005)	(0.68%)	(1.18%)
Panel C: Additional counterfactual targeting methods that were feasible in Togo in 2020				
Random	0.00	0.50	59%	30%
	(0.020)	(0.011)	(0.78%)	(1.34%%)
Occupation	-0.13	0.44	54%	21%
(Novissi)	(0.019)	(0.009)	(0.51%)	(0.88%%)
Occupation	0.50	0.73	76%	59%
(Optimal)	(0.015)	(0.006)	(0.71%)	(1.22%)

Supplementary Table 7 | Performance of targeting the hypothetical national program, with PMT as ground truth. Analysis is similar to that presented in the last four columns of Table 1, but with the PMT as ground truth instead of consumption.

Targeting Novissi in rural Togo – with rural PMT as ground truth
Based on 2020 Phone Survey (*N* = 8,915)

	Spearman	AUC	Accuracy	Precision & Recall
Panel A: Targeting methods considered by the Government of Togo in 2020				
Prefecture<br>(Admin-2 regions)	0.31<br>(0.023)	0.65<br>(0.011)	65%<br>(1.02%)	40%<br>(1.76%)
Canton<br>(Admin-3 regions)	0.19<br>(0.025)	0.60<br>(0.012)	62%<br>(1.03%)	34%<br>(1.78%)
Phone<br>(Expenditures)	0.16<br>(0.023)	0.58<br>(0.012)	61%<br>(1.02%)	33%<br>(1.76%)
Phone<br>(Machine Learning)	0.41<br>(0.022)	0.69<br>(0.012)	68%<br>(0.99%)	46%<br>(1.70%)
Panel B: Common alternative targeting methods that could not be implemented in Togo in 2020				
Asset Index	0.46<br>(0.021)	0.71<br>(0.011)	68%<br>(0.99%)	46%<br>(1.71%)
Panel C: Additional counterfactual targeting methods that were feasible in Togo in 2020				
Random	0.00<br>(0.023)	0.50<br>(0.012)	59%<br>(0.99%)	29%<br>(1.70%)
Occupation<br>(Novissi)	-0.12<br>(0.024)	0.45<br>(0.011)	55%<br>(0.93%)	23%<br>(1.60%)
Occupation<br>(Optimal)	0.26<br>(0.022)	0.61<br>(0.010)	65%<br>(0.66%)	40%<br>(1.14%)

Supplementary Table 8 | Performance of targeting Novissi in rural Togo, when using the rural-specific PMT as ground truth. Analysis is similar to that presented in the first four columns of Table 1, but with the rural-specific PMT (as described in Methods, 'Survey Data') as ground truth.

	Targeting Novissi in rural Togo<br>Based on 2020 Phone Survey (N = 8,915)				Hypothetical nationwide program<br>Based on 2018-2019 Field Survey (N = 4,171)			
	Spearman	AUC	Accuracy	Precision & Recall	Spearman	AUC	Accuracy	Precision & Recall
Prefecture<br>(Survey-recorded)	0.30<br>(0.017)	0.64<br>(0.008)	65%<br>(0.87%)	39%<br>(1.51%)	0.34<br>(0.017)	0.66<br>(0.008)	68%<br>(0.74%)	45%<br>(1.27%)
Canton<br>(Survey-recorded)	0.19<br>(0.019)	0.59<br>(0.009)	61%<br>(0.78%)	33%<br>(1.35%)	0.39<br>(0.016)	0.68<br>(0.008)	70%<br>(0.71%)	48%<br>(1.23%)
CDR Prefecture<br>(Phone-inferred)	0.23<br>(0.016)	0.61<br>(0.008)	63%<br>(0.76%)	36%<br>(1.32%)	0.27<br>(0.017)	0.63<br>(0.008)	67%<br>(0.74%)	44%<br>(1.40%)
CDR Canton<br>(Phone-inferred)	0.12<br>(0.021)	0.56<br>(0.011)	58%<br>(0.83%)	28%<br>(1.43%)	0.31<br>(0.017)	0.65<br>(0.008)	69%<br>(0.73%)	47%<br>(1.27%)
Phone<br>(Machine Learning)	0.38<br>(0.017)	0.70<br>(0.009)	69%<br>(0.87%)	47%<br>(1.18%)	0.45<br>(0.015)	0.73<br>(0.007)	71%<br>(0.74%)	50%<br>(1.26%)

Supplementary Table 9 | Geographic targeting with phone-inferred location. First two rows and final row replicate the results shown in Table 1. We add two additional counterfactual geographic targeting approaches based on location information derived from mobile phone data: targeting based on the average wealth of their home prefecture (row 3) or of their home canton (row 4). Home prefectures and cantons are inferred from outgoing mobile phone transactions as described in Supplementary Methods section 4; the poverty of associated with each prefecture and canton is taken from the poverty maps shown in Supplementary Figure 5.

	Prefecture-level	Canton-level
Survey ← → Voter	90.08%	69.77%
Survey ← → Phone Data	70.08%	46.56%
Voter ← → Phone Data	67.48%	44.89%

Supplementary Table 10 | Correlation between sources of location data in 2020 phone survey. Correlation between the three sources of home location data available for observations in the 2020 phone survey: self-reported location collected in a survey, voter location recorded at the time of voter registration, and home location inferred from phone data. Each entry represents the percentage of observations (without sample weights applied) for which the two datasets agree on the respondent's location. Percentages are taken among the population (N = 4,515) for whom all three data sources are available (that is, individuals who were surveyed, whose phone numbers were registered for the rural Novissi program so that the canton and prefecture associated with their voter ID are included in Novissi administrative data, and who place at least one outgoing call between March to September 2020 so that their phone number is tied to a home prefecture and canton). This analysis cannot be carried out for the 2018-2019 field survey as fewer than 15% of the phone numbers collected in the survey registered for the rural Novissi program.

	Share of phone transactions made from home prefecture (inferred from CDR)	Share of phone transactions made from home prefecture (self-reported in survey)
Panel A: 2018-2019 national household survey and April-June 2019 CDR		
Mean (and standard deviation)	75.46% (31.90%)	62.00% (40.05%)
Median	91.18%	81.84%
Mode	100.00%	100.00%
N	3,459,308	3,992
Panel A: 2020 phone survey and March-September 2020 CDR		
Mean (and standard deviation)	85.32% (18.78%)	68.00% (36.79%)
Median	94.00%	87.16%
Mode	100.00%	100.00%
N	5,615,393	8,183

Supplementary Table 11 | Percentage of mobile phone activity initiated from a subscriber's home prefecture. Table indicates the fraction of outgoing calls and text messages that are routed through a cell tower in the subscriber's home prefecture. In the first column, "home location" is inferred from the subscriber's CDR as described in Appendix B; in the second column, "home location" is recorded during a survey with the respondent. Panel A: results based on analysis from 2019, using CDR from three months in 2019 in the first column (N = 3,459,308), and survey respondents with known GPS coordinates from the 2018-2019 field survey in the second column (N = 3,992). Panel B: results based on analysis from 2020, using CDR from 7 months in 2020 in the left column (N = 5,615,393), and survey respondents with self-reported prefectures in the 2020 phone survey in the right column (N = 8,183).

Group	Response Rate	*N*
Panel A: Previous Novissi registration		
Registered	37.82%	15,402
Unregistered	25.61%	14,085
Panel B: Phone-inferred region		
Lomé Commune	35.45%	189
Maritime	40.83%	1,254
Plateaux	30.17%	3,627
Centrale	31.91%	702
Kara	35.31%	6,582
Savanes	30.42%	17,034
Panel C: Phone-predicted poverty (USD/day)		
<$1.32	33.50%	7,372
$1.32-$1.42	33.55%	7,372
$1.42-$1.57	30.10%	7,371
$1.57+	30.79%	7,372
Panel D: Phone expenditures (USD/day)		
<$0.03	22.56%	7,372
$0.03-$0.08	28.15%	7,372
$0.08-$0.21	34.82%	7,371
$0.21+	42.66%	7,372

Supplementary Table 12 | Response rates for 2020 phone survey. Response rate disaggregated by four dimensions: registration to a previous Novissi program (Panel A), region of Togo inferred from location of mobile phone transactions (Panel B), daily consumption inferred from mobile phone activity and machine learning (Panel C), and daily phone expenditures (Panel D).

Feature	Importance
Registered to previous Novissi program	15
Togocom subscriber	13
% nocturnal calls	10
% in Kpendjal	9
Active days	9
Mean balance of contacts	8
Median interactions per contact	7
Median time between calls (weekdays)	7
Active days (weekend)	6
Minimum time between calls	6

Table S13 | Feature importances for response reweighting model for 2020 phone survey. As described in Supplementary Methods section 5, the gradient boosting ensemble model is trained to predict the probability of response for a phone number drawn for the 2020 phone survey on the basis of pre-survey observable covariates (from CDR and previous Novissi registrations). Feature importance is calculated based on the total number of times a feature is split upon in the prediction ensemble.
