# Supplementary material for: Machine learning and phone data can improve targeting of humanitarian aid
Source: Nature. 2022 Mar 16;603(7903):864–70. doi: 10.1038/s41586-022-04484-9 (PMC8967719; doi:10.1038/s41586-022-04484-9)
Supplement: Supplementary file 2 — Reporting Summary. [file 41586_2022_4484_MOESM2_ESM.pdf]

nature portfolio

Corresponding author(s): Joshua Blumenstock

Last updated by author(s): Jan 5, 2022

Reporting Summary

Nature Portfolio wishes to improve the reproducibility of the work that we publish. This form provides structure for consistency and transparency in reporting. For further information on Nature Portfolio policies, see our Editorial Policies and the Editorial Policy Checklist.

Statistics

For all statistical analyses, confirm that the following items are present in the figure legend, table legend, main text, or Methods section.

n/a	Confirmed	
☐	☒	The exact sample size (*n*) for each experimental group/condition, given as a discrete number and unit of measurement
☐	☒	A statement on whether measurements were taken from distinct samples or whether the same sample was measured repeatedly
☒	☐	The statistical test(s) used AND whether they are one- or two-sided<br>*Only common tests should be described solely by name; describe more complex techniques in the Methods section.*
☐	☒	A description of all covariates tested
☒	☐	A description of any assumptions or corrections, such as tests of normality and adjustment for multiple comparisons
☒	☐	A full description of the statistical parameters including central tendency (e.g. means) or other basic estimates (e.g. regression coefficient) AND variation (e.g. standard deviation) or associated estimates of uncertainty (e.g. confidence intervals)
☒	☐	For null hypothesis testing, the test statistic (e.g. *F*, *t*, *r*) with confidence intervals, effect sizes, degrees of freedom and *P* value noted<br>*Give P values as exact values whenever suitable.*
☒	☐	For Bayesian analysis, information on the choice of priors and Markov chain Monte Carlo settings
☒	☐	For hierarchical and complex designs, identification of the appropriate level for tests and full reporting of outcomes
☒	☐	Estimates of effect sizes (e.g. Cohen's *d*, Pearson's *r*), indicating how they were calculated

Our web collection on statistics for biologists contains articles on many of the points above.

Software and code

Policy information about availability of computer code

Data collection	SurveyCTO version 2.7 was used to collect survey data.
Data analysis	Data were processed and analyzed using Python version 3.6. We used one specialized software package for processing mobile phone metadata, bandicoot version 0.6 (https://github.com/computationalprivacy/bandicoot). The code used for these analyses is publicly available in the GitHub repository located at https://github.com/emilylaiken/togo-targeting-replication/.

For manuscripts utilizing custom algorithms or software that are central to the research but not yet described in published literature, software must be made available to editors and reviewers. We strongly encourage code deposition in a community repository (e.g. GitHub). See the Nature Portfolio guidelines for submitting code & software for further information.

Data

Policy information about availability of data

All manuscripts must include a data availability statement. This statement should provide the following information, where applicable:

- Accession codes, unique identifiers, or web links for publicly available datasets
- A description of any restrictions on data availability
- For clinical datasets or third party data, please ensure that the statement adheres to our policy

The data used in this analysis include data that are available from public online repositories, data that are available upon request of the data provider, and data that are not publicly available because of restrictions by the data provider. The micro-estimates of wealth and population density used to derive satellite-based poverty maps are available from the Humanitarian Data Exchange (https://data.humdata.org/dataset/relative-wealth-index and https://data.humdata.org/dataset/highresolutionpopulationdensitymaps-tgo). The survey datasets are available upon request from the Institut National de la Statistique et des Études Economiques et Démographiques (https://inseed.tg/ and inseed@inseed.tg). The mobile phone data and administrative data from the Novissi program contain proprietary and

sensitive information, and cannot be publicly released. Upon request, we can provide information to researchers on how to contact mobile network operators and the Togolese government to request these datasets.

Field-specific reporting

Please select the one below that is the best fit for your research. If you are not sure, read the appropriate sections before making your selection.

☐ Life sciences ☒ Behavioural & social sciences ☐ Ecological, evolutionary & environmental sciences

For a reference copy of the document with all sections, see nature.com/documents/nr-reporting-summary-flat.pdf

Behavioural & social sciences study design

All studies must disclose on these points even when the disclosure is negative.

Study description	This study is a quantitative analysis that compares targeting outcomes - including exclusion errors, total social welfare, and measures of fairness - under different targeting regimes.
Research sample	Our study involves several distinct samples:<br>(a) Respondents to a 2018-19 field survey, which is nationally representative.<br>(b) Respondents to a 2020 phone survey, a sample that is representative of mobile network subscribers inferred to be living in aid-eligible areas of Togo.<br>(c) Data from the population of mobile phone users in Togo, which is a comprehensive dataset of all mobile phone subscribers in the country.<br><br>We perform heterogeneity analyses by urban/rural location and by gender.
Sampling strategy	2018-19 field survey: Stratified random sample of households across Togo (stratified by region and urban vs. rural).<br>2020 phone survey: Random sample of phone subscribers in Togo's 100 poorest cantons. (see Methods Section 3 and Supplementary Materials Section 5.iii for full details).<br><br>The sample size for both the surveys was determined on the basis of a budget constraint for survey data collection.
Data collection	2018-19 field survey: Data was collected in-person at the sampled household by enumerators from Togo's INSEED. The full survey instrument is available at https://phmecv.uemoa.int/nada/index.php/catalog/50/related-materials.<br>2020 phone survey: Data was collected via mobile phone calls with enumerators from Togo's INSEED. The full survey instrument is available at https://jblumenstock.com/files/papers/TogoInstrument2020.pdf
Timing	2018-19 field survey: The data collection took place in two waves. The first wave lasted from September 25, 2018 to December 10, 2018. The second wave lasted from April 4, 2019 to June 24, 2019.<br>2020 phone survey: The data collection took place between September 24 and October 12, 2020.
Data exclusions	2018-19 field survey: 1 observation is excluded from analysis. This respondent did not answer the consumption aggregate question that is used as the ground-truth poverty measure for targeting simulations.<br>2020 phone survey: A total of 1,786 observations are excluded from analysis. 1,180 surveys are removed due to low data quality (details on calculations of data quality are included in Supplementary Materials Section 5.iii). A further 606 surveys are dropped due to missing data. These respondents either (a) did not respond to all questions that make up the components of the PMT, or (b) did not respond to one or more of the questions that were used in counterfactual targeting simulations (including occupation, prefecture, canton, and asset index components).
Non-participation	2018 field survey: As the data was collected by INSEED prior to our participation in the project, we do not have information about rates of non-participation in the 2018 field survey.<br>2020 phone survey: Enumerators conducted 10,701 interviews out of 30,244 phone numbers that were called, for an overall response rate of 35.38%.
Randomization	Participants were not randomized into groups for this study

Reporting for specific materials, systems and methods

We require information from authors about some types of materials, experimental systems and methods used in many studies. Here, indicate whether each material, system or method listed is relevant to your study. If you are not sure if a list item applies to your research, read the appropriate section before selecting a response.

Materials & experimental systems

n/a	Involved in the study
☒	☐ Antibodies
☒	☐ Eukaryotic cell lines
☒	☐ Palaeontology and archaeology
☒	☐ Animals and other organisms
☐	☒ Human research participants
☒	☐ Clinical data
☒	☐ Dual use research of concern

Methods

n/a	Involved in the study
☒	☐ ChIP-seq
☒	☐ Flow cytometry
☒	☐ MRI-based neuroimaging

Human research participants

Policy information about studies involving human research participants

Population characteristics	The 2018-19 field survey was a nationally-representative household survey. The 2020 phone survey was a random sample of phone subscribers inferred to be living in Togo's 100 poorest cantons. Summary statistics of these two populations are provided in Table S11.
Recruitment	For the 2018-19 survey, respondents were approached at their place of residence by Togo's Institut National de la Statistique et des Études Economiques et Démographiques (INSEED). For the 2020 phone survey, respondents were called from a call center run by INSEED. Nonresponse bias for the 2018-19 field survey could arise from sampled households who were unavailable at the time of the survey or preferred not to talk with enumerators from the governmental statistical institute. Nonresponse bias for the 2020 phone survey could arise from mobile network subscribers whose phones were broken or off, those who did not respond to calls from unknown numbers, and those who did not wish to speak with enumerators from the governmental statistical institute. Importantly, the 2020 phone survey also does not cover people who do not own phones.
Ethics oversight	Human subjects research was reviewed and approved by U.C. Berkeley's Committee for the Protection of Human Subjects under protocol 2020-05-13281.

Note that full information on the approval of the study protocol must also be provided in the manuscript.
